# Fluorescent carbazole-derived α-amino acids: structural mimics of tryptophan†

**DOI:** 10.1039/d4sc01173b

**Published:** 2024-03-21

**Authors:** Rebecca Clarke, Liyao Zeng, Bethany C. Atkinson, Malcolm Kadodwala, Andrew R. Thomson, Andrew Sutherland

**Affiliations:** a School of Chemistry, University of Glasgow Joseph Black Building, University Avenue Glasgow G12 8QQ UK Drew.Thomson@glasgow.ac.uk Andrew.Sutherland@glasgow.ac.uk

## Abstract

Fluorescent tags are commonly used for imaging of proteins and peptides during biological events; however, the large size of dyes can disrupt protein structure and function, and typically require the use of a chemical spacer. Herein, we report the synthesis of a new class of fluorescent unnatural α-amino acid, containing carbazole side-chains designed to mimic l-tryptophan and thus, readily incorporated into peptides. The amino acids were constructed using a Negishi cross-coupling reaction as the key step and exhibited strong fluorescent emission, with high quantum yields in both organic solvents and water. Compatible with solid phase peptide synthesis, the carbazole amino acids were used to replace tryptophan in a β-hairpin model peptide and shown to be a close structural mimic with retention of conformation. They were also found to be effective fluorescent molecular reporters for biological events. Incorporation into a proline-rich ligand of the WW domain protein demonstrated that the fluorescent properties of a carbazole amino acid could be used to measure the protein–protein binding interaction of this important biological signalling process.

## Abstract 2

Unnatural α-amino acids bearing carbazole side-chains have been shown to be effective structural mimics of tryptophan in peptides and valuable fluorescent probes for the analysis of protein–protein interactions.

## Graphical abstract

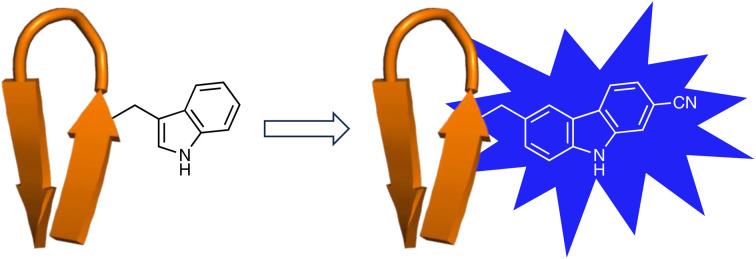

## Introduction

α-Amino acids are the basic building blocks of life, acting as the key structural components of peptides and proteins.^1^ They are essential for all enzyme mechanisms and are involved in many biological processes, such as cellular metabolism and signal induction pathways. The fundamental biological role of α-amino acids has resulted in their general use in biomedical and life sciences research^2^ and in a wide range of industrial applications in the food, pharmaceutical and agrochemical sectors.

**Fig. 1:**
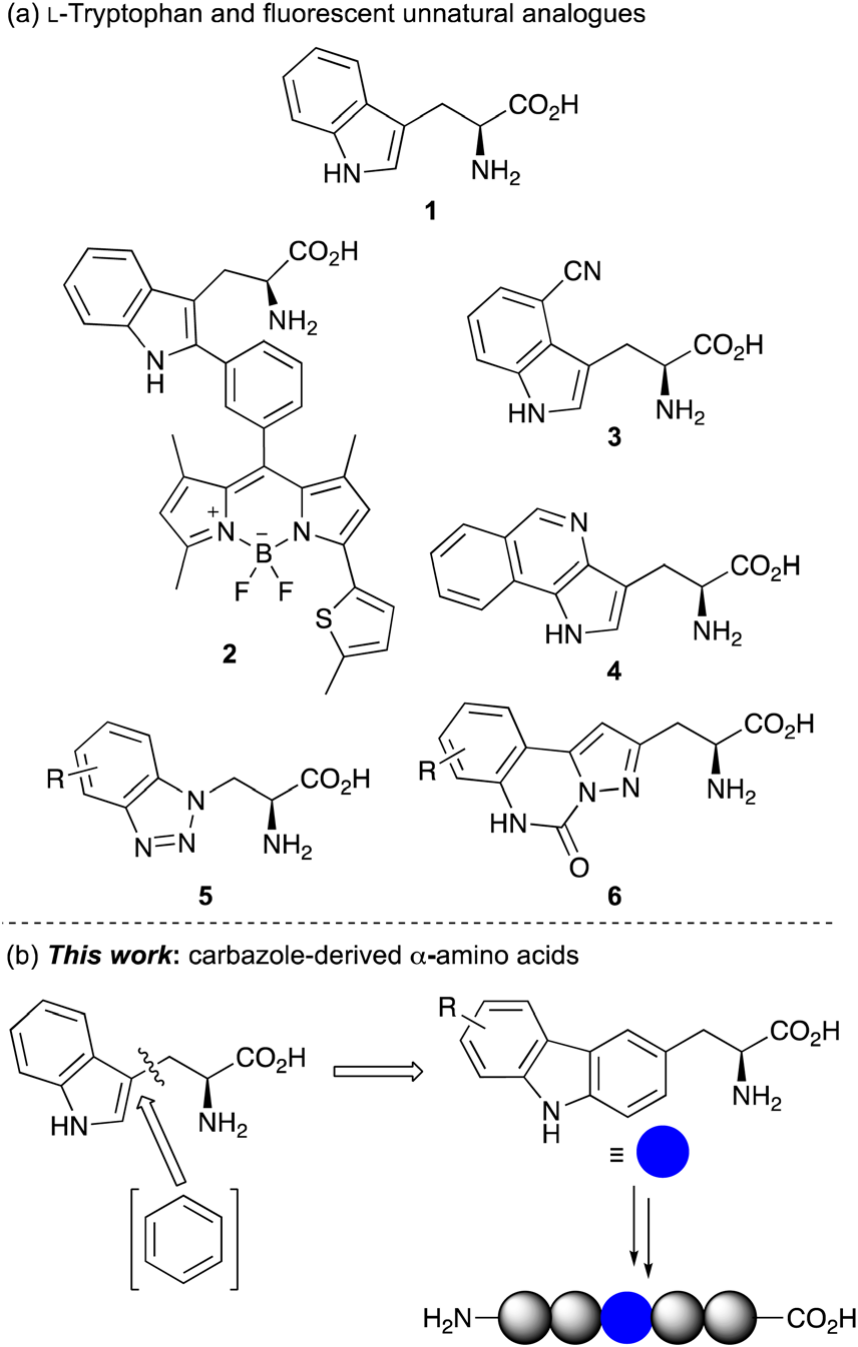
(a) l-Tryptophan (1) and selected fluorescent unnatural analogues. (b) Design of carbazole-derived α-amino acids for incorporation into peptides.

In combination with fluorescent spectroscopy, α-amino acids have also been used for molecular imaging, providing a method to study and visualise cellular processes in real time.^3^ However, applications of fluorescent, naturally occurring α-amino acids l-phenylalanine, l-tyrosine and l-tryptophan (1) (Fig. 1a) are limited due to their poor photoluminescent properties.^3*a*^ Furthermore, their occurrence at multiple sites within a protein can complicate spectroscopic analysis. To overcome these issues, many studies utilise extrinsic fluorescent labels.^4^ However, these are typically large chromophores, requiring a chemical spacer to minimise disruption of protein structure and function. Furthermore, these fluorescent dyes are generally positioned at the terminus of a protein limiting applications. For these reasons, there has been significant focus on the development of fluorescent unnatural α-amino acids.^3,5^ As well as the facile modification of side-chain chromophores that can be tuned for a particular fluorescent application, the fact that these have an amino acid core means that they can be selectively embedded within proteins using solid phase peptide synthesis (SPPS) or genetic encoding, for a range of spectroscopic experiments. As l-tryptophan has the strongest fluorescent properties of the proteinogenic amino acids, a key strategy in developing fluorescent unnatural analogues is the structural modification of the indole side chain.^6,7^ This approach has been exemplified by the Vendrell group who incorporated BODIPY chromophores at C-2 of the tryptophan indole ring using palladium-catalyzed C–H activation methods.^8^ Adducts such as 2 have been used for the optical imaging of chemotherapy-induced cancer cell death.^8*e*^ Other fluorescent analogues of l-tryptophan include l-4-cyanotryptophan (3),^9^ which has a long fluorescence lifetime and was used to measure peptide-membrane binding constants, and (pyrrolo)isoquinoline 4,^10^ that replaced tryptophan in *E. coli* dihydrofolate reductase while retaining enzyme activity.

Inspired by this work, we have sought to develop fluorescent analogues of l-tryptophan for various biological applications and have reported amino acids with benzotriazole 5 and pyrazolo-quinazoline 6 chromophores (Fig. 1a).^11^ While these have useful fluorescent properties compared to l-tryptophan, unnatural amino acids that act as a close structural analogue of l-tryptophan in proteins but with brighter fluorescence for more general biology applications are still required. In designing a novel amino acid side chain, we proposed that the insertion of an aromatic ring between the amino acid core and indole ring to give a carbazole moiety (Fig. 1b), would result in a structure, which could be used as a close structural mimic of l-tryptophan in peptides. Furthermore, the extended and conformationally rigid conjugation of the carbazole moiety would generate bright chromophores that could find general application as fluorescent probes. Based on our interests, we were particularly interested in developing fluorescent amino acid probes that could be used for investigating protein–protein interactions of important signalling processes. We now report the synthesis of novel carbazole-derived α-amino acids and demonstrate the use of these compounds as structural mimics of l-tryptophan by successful incorporation into a tryptophan zipper peptide. We also describe the photophysical properties of these amino acids and establish their role as fluorescent reporters of molecular binding events, such as the measurement of protein–protein interactions.

## Results and discussion

**Scheme 1:**
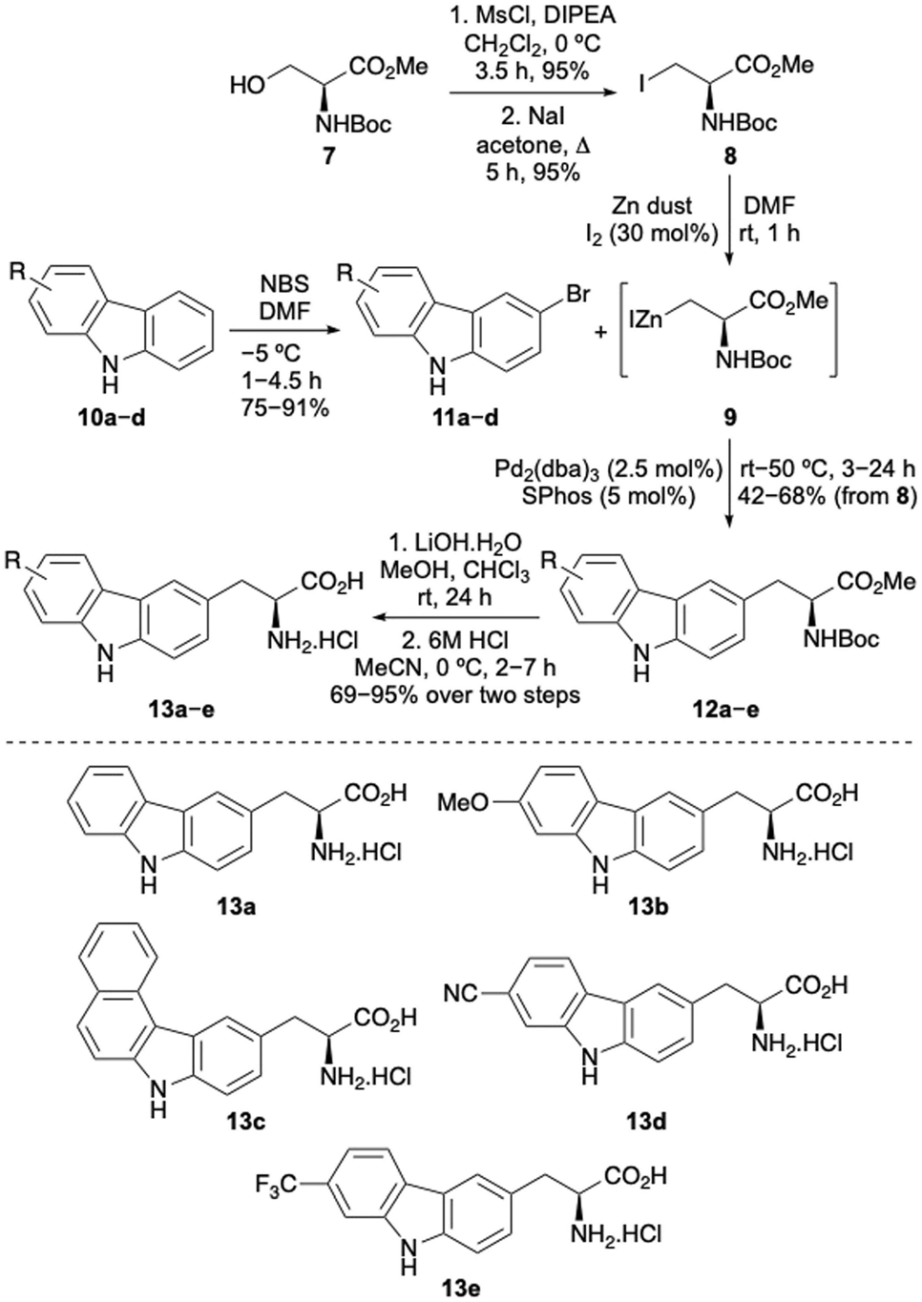
Chemical synthesis of carbazole-derived α-amino acids.

The first stage of this project investigated the development of a concise route to access a focused library of carbazole α-amino acids.^12^ Various strategies were considered and it was proposed that the Negishi coupling of halo-substituted carbazoles with an organozinc reagent derived from a β-iodoalanine derivative would allow rapid access to the amino acid targets.^13^ Precedent for the use of this type of β-alanine anion reagent, developed by Jackson and co-workers, has been shown with coupling to various halogenated N-heterocycles such as pyridines and *N*-protected indoles.^13,14^ Initially, a series of carbazoles (10) were prepared in a two-step approach by Suzuki–Miyaura coupling of 1-bromo-2-nitrobenzenes with various arylboronic acids,^15^ followed by Cadogan cyclisation performed using triphenylphosphine (see ESI†).^16^ Regioselective bromination using *N*-bromosuccinimide (NBS) then gave bromocarbazoles 11a–d in 75–91% yields (Scheme 1). Organozinc reagent 9 was prepared in three steps from commercially available serine derivative 7 by activation of the primary alcohol as a mesylate, displacement with iodide and then treatment with iodine-activated zinc dust.^13,17^ Using SPhos as a ligand,^18^ which Jackson and co-workers propose forms a monoligated palladium species, the Negishi cross-coupling of bromocarbazoles with organozinc reagent 9 was investigated. Surprisingly, *N*-protection of the carbazoles was not required and with a Pd_2_(dba)_3_ loading of 2.5 mol%, successful coupling was observed to give adducts 12a–e in moderate to good yields from β-iodoalanine 8.^19,20^ Ester hydrolysis using lithium hydroxide and acid-mediated removal of the Boc-protecting group, under mild conditions, completed the synthesis of carbazole amino acids 13a–e. This general approach permitted the straightforward preparation of a novel class of unnatural α-amino acid incorporating a carbazole side chain, with either electron-rich or electron-deficient substituents.

**Fig. 2:**
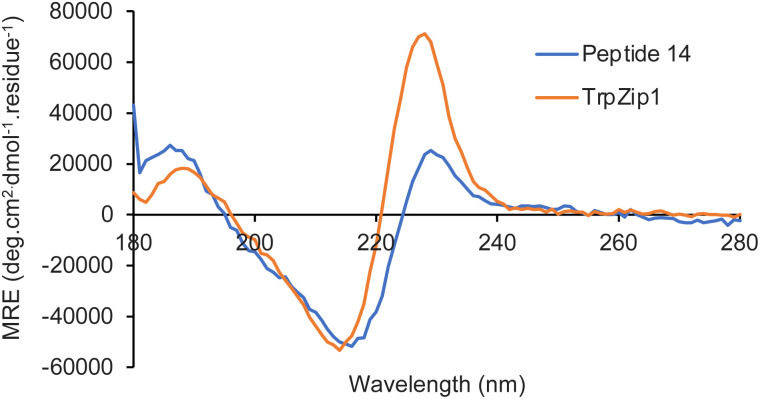
CD spectra for TrpZip1 and modified peptide 14.

Following successful preparation of the carbazole α-amino acids, these were investigated as structural mimics of tryptophan. The effect of substituting amino acid 13e for a tryptophan residue in a well-characterised designed peptide was studied. The beta hairpin TrpZip1 was chosen as a model of beta structure.^21^ An Fmoc-derivative of amino acid 13e was prepared under standard conditions and incorporated into the peptide using SPPS protocols. The resulting peptide, 14 (Table 1) was purified by reverse phase HPLC and characterised using high resolution electrospray ionisation mass spectrometry. In the peptide, the substitution preserved the original secondary structure, as demonstrated by circular dichroism (CD) spectroscopy. The CD spectrum for the TrpZip1 mimic, peptide 14 exhibited a characteristic minimum at 214 nm and maximum at 228 nm, indicative of a beta hairpin conformation and ordered aromatic side chains (Fig. 2). Peptide 14 also exhibited a near-UV CD signal (*ca.* 280–310 nm), again indicative of ordered aromatic side chains. These spectroscopic features are consistent with the formation of a stable beta hairpin structure, as in the parent TrpZip1 peptide.^21^ Thus, carbazole α-amino acid 13e was found to be highly compatible with SPPS, and could be selectively embedded within a peptide, replacing tryptophan, while retaining secondary structure.

**Table 1:** Peptide sequences of TrpZip1 and TrpZip1 modified with amino acid 13e

Peptide	Sequence
TrpZip1	H-Ser-Trp-Thr-Trp-Glu-Gly-Asn-Lys-Trp-Thr-Trp-Lys-NH_2_
14	H-Ser-Trp-Thr-Trp-Glu-Gly-Asn-Lys-13e-Thr-Trp-Lys-NH_2_

**Fig. 3:**
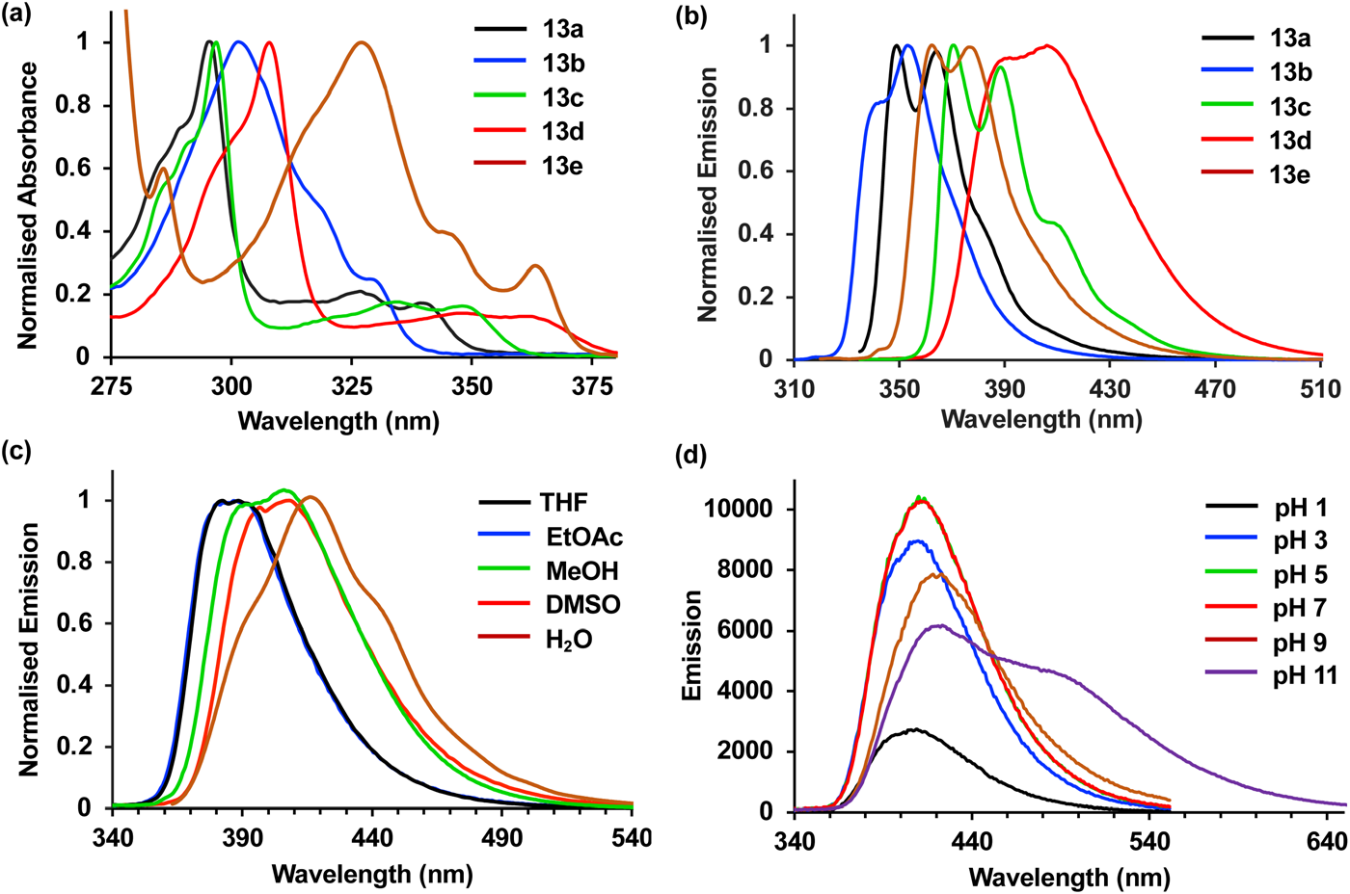
(a) Absorption spectra of amino acids 13a–13e (5 μM) in methanol. (b) Normalised emission spectra of amino acids 13a–13e (5 μM) in methanol. (c) Normalised emission spectra of 13d (5 μM) in various solvents. (d) Emission spectra of 13d (5 μM) at various pH.

Next, the photophysical properties of amino acids 13a–13e were measured.^22,23^ As it was important to identify an amino acid that could be used as a probe for different applications, the photophysical properties were assessed in both an organic solvent (methanol) and water (Table 2). The absorption spectra of amino acids 13a–13e showed well-resolved vibrational bands, indicative of rigid chromophores at wavelengths more red-shifted compared to proteinogenic amino acids (Fig. 3a). Similarly, strong emission spectra at wavelengths from 350–419 nm and with mirrored vibrational bands were observed, with 13d the exception, which displayed broadened emission from a combination of locally excited and internal charge transfer states (Fig. 3b). For amino acids 13a–13d, the quantum yields ranged from 0.28 to 0.76 and while lower values were observed in water, good levels of brightness were maintained. Trifluoromethyl analogue 13e was the only amino acid that displayed a low quantum yield (0.06) in methanol. Interestingly, both the molar attenuation coefficient (*ε*) and quantum yield of 13e were enhanced in water, resulting in a near 5-fold increase in brightness. These results suggest this amino acid has the potential to be used as an environment sensitive on-off probe.

**Table 2:** Photophysical data of amino acids 13a–e

Amino acid	Solvent	*λ*_Abs_ a (nm)	*ε* (cm^−1^ M^−1^)	*λ*_Em_ a (nm)	*Φ*_F_ b	Brightness (cm^−1^ M^−1^)
13a	MeOH	342	16 500	350, 366	0.31	5120
	Water	341	8800	352, 366	0.28	2460
13b	MeOH	329	12 700	354	0.35	4450
	Water	331	12 100	357	0.43	5200
13c	MeOH	351	16 100	371, 388	0.76	12 240
	Water	362	10 900	377, 390	0.59	6430
13d	MeOH	363	22 300	393, 409	0.39	7250
	Water	361	19 600	419	0.37	4450
13e	MeOH	366	11 600	365, 378	0.06	700
	Water	348	14 000	380	0.24	3360

a Spectra were recorded at 5 μM.

b Quantum yields (*Φ*_F_) were determined using anthracene and l-tryptophan as standards.

The solvatochromism and pH sensitivity of three of the amino acids, 13c–13e were further analysed (see ESI†).^24^ Although amino acids 13c and 13e displayed minimal solvatochromism with emission independent of solvent polarity, cyano analogue 13d was found to be sensitive to the solvent used, with a bathochromic shift of emission in more polar solvents (Fig. 3c). For example, an emission maximum was observed at 382 nm in THF, while this shifted to 419 nm in water. These results confirm the charge transfer character of the excited state of 13d, which is stabilised in more polar solvents. Cyano analogue 13d also displayed greatest sensitivity to pH change. While the absorption spectra for 13d was remarkably consistent from pH 1 to 11, the emission spectra showed major differences (Fig. 3d). At pH 1, protonation of the carbazole amine and inhibition of charge transfer resulted in a 5-fold suppression in emission compared to pH 7. In contrast, at pH 11, the generation of a more anionic species with increased charge transfer character led to a new, red-shifted band at ∼500 nm. Overall, these photophysical studies identified cyano analogue 13d as the lead fluorescent amino acid. With the most red-shifted absorption and emission wavelengths, largest Stokes shifts, consistent quantum yields in methanol and water, and both solvatochromic and pH sensitivity, this amino acid was deemed suitable for further development as a fluorescent probe.

**Fig. 4:**
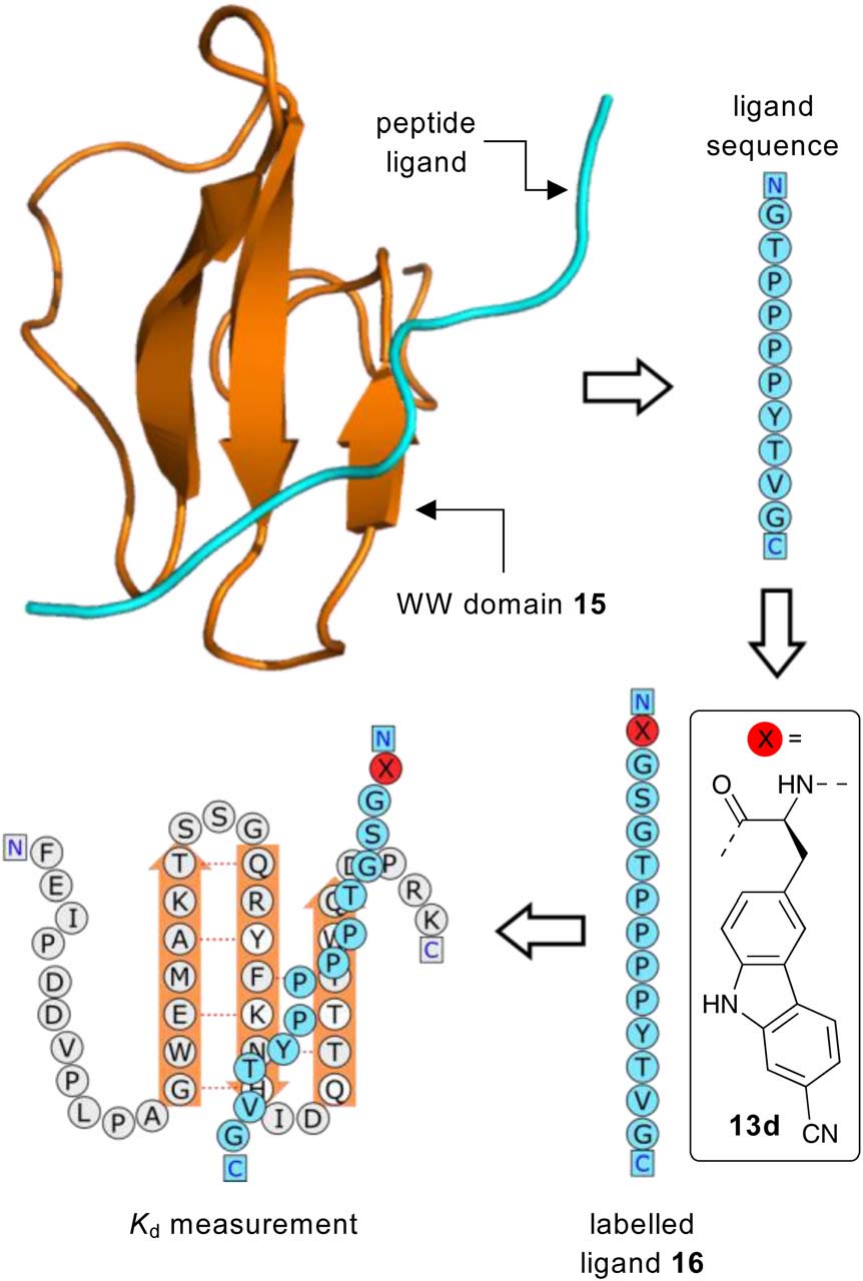
Proposed strategy for the use of amino acid 13d as a fluorescent reporter group for the binding of a WW domain with its peptide ligand.

**Fig. 5:**
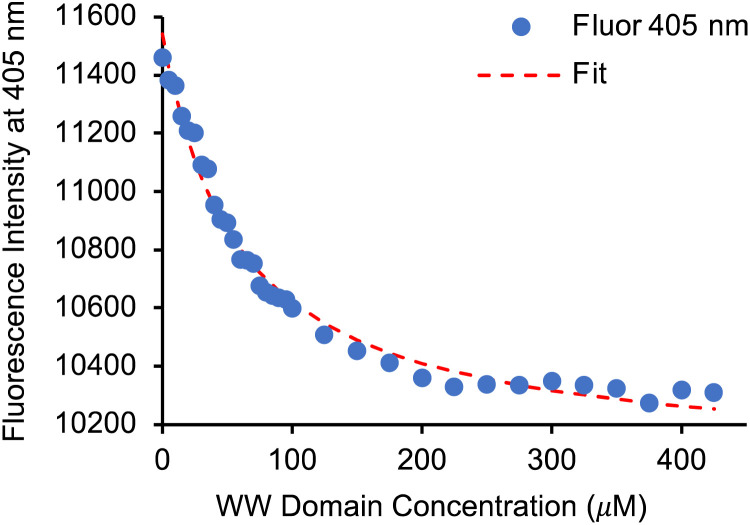
Fluorescence titration data of binding of modified peptide ligand 16 with WW domain 15.

Having identified 13d as the amino acid with the best overall photophysical properties, the potential of this amino acid to act as a reporter of molecular binding events was explored. WW domains are small (*ca.* 50 residue) proteins that are known to form transient complexes with proline rich peptide sequences as part of important biological signaling processes, including ubiquitination.^25^ It was proposed that incorporation of amino acid 13d at the N-terminus of a proline-rich peptide ligand would generate a probe that could be used determine binding with its WW domain (Fig. 4). To this end, a known WW domain protein sequence 15 and its proline-rich ligand,^26^ with amino acid 13d incorporated at the N-terminus (16) were synthesised. Both the WW domain protein 15 and the modified peptide ligand 16 were prepared using standard SPPS protocols, purified using reverse phase HPLC and characterised using mass spectrometry (see ESI†). A fluorescence titration was performed in which the concentration of ligand 16 was maintained at a constant 10 μmol L^−1^, and the concentration of WW domain protein 15 was varied from 0–400 μmol L^−1^ (Fig. 5). Changes to the fluorescence signal from the ligand were monitored at 405 nm and were found to vary as a function of WW domain concentration. Fitting the titration data to a Hill–Langmuir model gave a *K*_d_ value of 59 μM, which is consistent with the literature result of 52 ± 5 μM,^26^ indicating the inclusion of amino acid 13d did not perturb the binding event. This study demonstrates the application of these carbazole α-amino acids as fluorescent molecular reporters for biological events such as the measurement of protein–protein interactions.

## Conclusions

In conclusion, a new class of fluorescent unnatural α-amino acid bearing carbazole side-chains has been prepared using the Negishi cross-coupling reaction of halogenated carbazoles and an organozinc reagent derived from a β-iodoalanine derivative as the key step. The role of these amino acids as effective mimics of tryptophan was verified by replacement of a Trp residue in a beta hairpin TrpZip1 peptide. The application of these unnatural amino acids as fluorescent probes for analysis and measurement of biological processes was also established. The rigid, extended conjugation of the substituted carbazole moieties in relation to the indole side-chain of the natural analogue displayed red-shifted absorption and emission spectra, as well as high quantum yields in both organic and aqueous environments. Cyano analogue 13d, which displayed charge transfer-based fluorescence and environment sensitivity was incorporated into the proline-rich ligand of the WW domain protein and shown to be an effective fluorescent probe for measurement of this binding interaction. As well as tryptophan mimics and fluorescent reporters of biological processes, this work has also shown that these readily accessible unnatural amino acids are highly compatible with SPPS, easily incorporated into designed peptides for various applications. Future work will investigate this feature of the carbazole-derived α-amino acids, with the SPPS preparation of modified peptides for fluorescent spectroscopic analysis of other chemical biology events.

## Data availability

All experimental and characterisation data, as well as photophysical and NMR spectra are available in the ESI.†

## Author contributions

M. K., A. R. T., and A. S. conceived the project. R. C. performed the synthesis of amino acids 13a–13e, prepared peptide 14, conducted photophysical analysis and CD spectroscopy. L. Z. performed synthesis of 13e and S2, prepared peptide 16, conducted CD spectroscopy and binding assays. B. C. A. prepared WW domain peptide 15. A. R. T. and A. S. wrote the manuscript with contributions from all authors.

## Conflicts of interest

There are no conflicts to declare.

## Supplementary Material

SC-015-D4SC01173B-s001
