# Supplementary material for: Fluorescent carbazole-derived α-amino acids: structural mimics of tryptophan
Source: Chem Sci. 2024 Mar 21;15(16):5944–9. doi: 10.1039/d4sc01173b (PMC11040653; doi:10.1039/d4sc01173b)

Electronic Supplementary Material (ESI) for Chemical Science.
This journal is © The Royal Society of Chemistry 2024

Supporting Information for:

Fluorescent Carbazole-Derived α-Amino Acids; Structural Mimics of Tryptophan

Rebecca Clarke, Liyao Zeng, Bethany C. Atkinson, Malcolm Kadodwala, Andrew R. Thomson, and Andrew Sutherland**

School of Chemistry, The Joseph Black Building, University of Glasgow,

Glasgow G12 8QQ, United Kingdom.

Table of Contents

1. General Experimental S2
2. Experimental Procedures and Spectroscopic Data for all Compounds S2–S18
3. Peptide Synthesis S19–S22
4. Fluorescence Titration S23
5. Photophysical Data for α-Amino Acids **13a**–**e** S24–S35
6. References S36
7. ^{1}H and ^{13}C{^{1}H} NMR Spectra for all Compounds S37–S86

1. General Experimental

All reagents and starting materials were obtained from commercial sources and used as received. Dry solvents were purified using a PureSolv 500 MD solvent purification system. All reactions were performed under an atmosphere of argon unless otherwise mentioned. Brine refers to a saturated solution of sodium chloride. Flash column chromatography was carried out using Fisher matrix silica 60. Macherey-Nagel aluminium-backed plates pre-coated with silica gel 60 (UV_{254}) were used for thin layer chromatography and visualised by staining with $KMnO_4$, vanillin or ninhydrin. ^{1}H NMR and ^{13}C NMR spectra were recorded on a Bruker DPX 400 or 500 spectrometer with chemical shift values in ppm relative to TMS (δ_H 0.00 and δ_C 0.0), or residual $CDCl_3$ (δ_H 7.26 and δ_C 77.2), DMSO-d_6 (δ_H 2.50 and δ_C 39.5) or CD_3OD (δ_H 3.31 and δ_C 49.0) as standard. ^{1}H and ^{13}C assignments are based on two-dimensional COSY and DEPT experiments, respectively. Mass spectra were obtained using a JEOL JMS-700 spectrometer for EI and CI or Bruker Microtof-q for ESI. Infrared spectra were obtained neat using a Shimadzu IR Prestige-21 spectrometer. Melting points were determined on a Reichert platform melting point apparatus. Optical rotations were determined as solutions irradiating with the sodium D line (λ= 589 nm) using an Autopol V polarimeter. $[\alpha]_D$ values are given in units 10^{-1} deg cm^2 g^{-1}.

2. Experimental Procedures and Spectroscopic Data for all Compounds

Synthesis of Carbazoles:

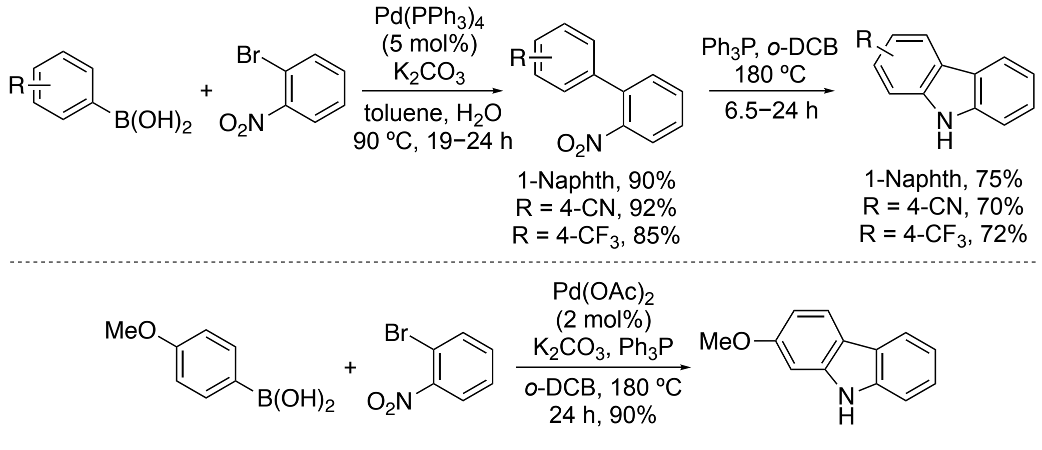

1’-(2-Nitrophenyl)naphthalene[1]

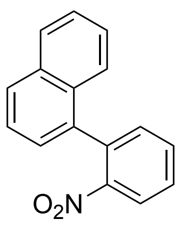

To a round bottomed flask was added 1-bromo-2-nitrobenzene (100 mg, 0.500 mmol), naphthalene-1-boronic acid (93.0 mg, 0.540 mmol), potassium carbonate (138 mg, 1.00 mmol), toluene (0.6 mL) and water (0.4 mL). The reaction mixture was degassed for 15 minutes with argon. Tetrakis(triphenylphosphine)palladium(0) (6.00 mg, 0.00495 mmol) was added and degassing continued for a further 15 minutes. The reaction mixture was then heated to 90 °C and stirred for 24 h. The reaction mixture was cooled to room temperature, diluted with dichloromethane (50 ml) and washed with water (30 mL) and brine (30 mL). The organic layers were combined, dried over magnesium sulfate and concentrated under vacuum. Purification by silica gel column chromatography (hexane:diethyl ether, 4:1) gave 1’-(2-nitrophenyl)naphthalene as a white solid (112 mg, 0.450 mmol, 90%). R_f = 0.33 (hexane:diethyl ether, 4:1); Mp 90–92 °C (lit.[1] 92–93 °C); ^{1}H NMR (400 MHz, $CDCl_3$) δ 8.09 (dd, J = 8.1, 1.2 Hz, 1H, 3-H), 7.94–7.90 (m, 2H, 2’-H and 8’-H), 7.71 (td, J = 7.0, 1.2 Hz, 1H, 5-H), 7.63–7.58 (m, 1H, 4-H), 7.56–7.39 (m, 5H, 3’-H, 4’-H, 5’-H, 6’-H and 7’-H), 7.36 (dd, J = 7.0, 1.1 Hz, 1H, 6-H); ^{13}C NMR (101 MHz, $CDCl_3$) δ 149.9 (C), 135.6 (C), 135.4 (C), 133.6 (C), 133.2 (CH), 132.7 (CH), 131.6 (C), 128.8 (CH), 128.7 (CH), 128.6 (CH), 126.7 (CH), 126.20 (CH), 126.19 (CH), 125.4 (CH), 125.0 (CH), 124.4 (CH); MS (ESI) *m/z* 272 (M + Na^+, 100).

2-Nitro-[1,1'-biphenyl]-4’-carbonitrile[2]

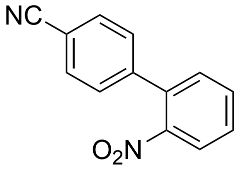

To a round bottomed flask was added 1-bromo-2-nitrobenzene (2.00 g, 9.90 mmol), 4-cyanophenylboronic acid (1.60 g, 10.9 mmol), potassium carbonate (2.74 g, 19.8 mmol), toluene (12 mL) and water (8 mL). The reaction mixture was degassed for 15 minutes with argon. Tetrakis(triphenylphosphine)palladium(0) (114 mg, 0.0990 mmol) was added and degassing continued for a further 15 minutes. The reaction mixture was then heated to 90 °C and stirred for 19 h. The reaction mixture was cooled to room temperature, diluted with dichloromethane (50 mL) and washed with water (50 mL) and brine (50 mL). The organic layers were combined, dried over magnesium sulfate and concentrated under vacuum. Purification by silica gel column chromatography (dichloromethane:hexane, 1.5:1) gave 2-nitro-[1,1'-biphenyl]-4’-carbonitrile as a yellow solid (2.05 g,

9.12 mmol, 92%). R$_f$ = 0.21 (CH_2Cl_2:hexane, 1.5:1); Mp 137–139 °C (lit.[2] 140–142 °C); ^{1}H NMR (400 MHz, $CDCl_3$) δ 7.98 (dd, J = 8.1, 1.3 Hz, 1H, 3-H), 7.72 (d, J = 8.6 Hz, 2H, 3'-H and 5'-H), 7.69 (dd, J = 7.6, 1.3 Hz, 1H, 6-H), 7.61–7.56 (m, 1H, 5-H), 7.45–7.40 (m, 3H, 4-H, 2'-H and 6'-H); ^{13}C NMR (101 MHz, $CDCl_3$) δ 148.7 (C), 142.5 (C), 134.9 (C), 133.0 (CH), 132.4 (2 × CH), 131.7 (CH), 129.5 (CH), 128.9 (2 × CH), 124.7 (CH), 118.5 (C), 112.2 (C); MS (ESI) *m/z* 247 (M + Na^+, 100).

2-Nitro-4'-(trifluoromethyl)-1,1'-biphenyl

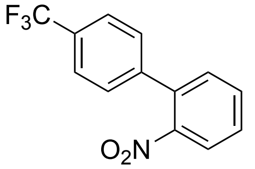

To a round bottomed flask was added 1-bromo-2-nitrobenzene (100 mg, 0.500 mmol), 4-(trifluoromethyl)phenylboronic acid (104 mg, 0.550 mmol), potassium carbonate (138 mg, 1.00 mmol), toluene (0.6 mL) and water (0.4 mL). The reaction mixture was degassed for 15 minutes with argon. Tetrakis(triphenylphosphine)palladium(0) (6.00 mg, 0.00495 mmol) was added and degassing continued for a further 15 minutes. The reaction mixture was then heated to 90 °C and stirred for 23 h. The reaction mixture was cooled to room temperature, diluted with dichloromethane (30 mL) and washed with water (30 mL) and brine (30 mL). The organic layers were combined, dried over magnesium sulfate and concentrated under vacuum. Purification by silica gel column chromatography (hexane:diethyl ether, 2:1) gave 2-nitro-4'-(trifluoromethyl)-1,1'-biphenyl as a yellow solid (113 mg, 0.42 mmol, 85%). R$_f$ = 0.34 (hexane:diethyl ether, 2:1); Mp 34–35 °C; IR (neat) 1525, 1323, 1122, 1109, 1068 cm^{-1}; ^{1}H NMR (400 MHz, $CDCl_3$) δ 7.94 (dd, J = 8.2, 1.2 Hz, 1H, 3-H), 7.70 (d, J = 8.2 Hz, 3'-H and 5'-H), 7.66 (dd, J = 7.5, 1.3 Hz, 1H, 6-H), 7.58–7.52 (m, 1H, 5-H), 7.48–7.41 (m, 3H, 4-H, 2'-H and 6'-H); ^{13}C NMR (101 MHz, $CDCl_3$) δ 148.9 (C), 141.5 (C), 135.2 (C), 132.9 (CH), 131.9 (CH), 130.3 (q, $^2J_{C\text{-}F}$ = 32.6 Hz, C), 129.1 (CH), 128.5 (2 × CH), 125.6 (q, $^3J_{C\text{-}F}$ = 3.8 Hz, 2 × CH), 124.5 (CH), 124.2 (q, $^1J_{C\text{-}F}$ = 281.8 Hz, C); ^{19}F NMR (377 MHz, $CDCl_3$) δ –62.5 (s); MS (ESI) *m/z* 290 (M + Na^+, 100); HRMS (ESI) *m/z*: [M + Na]$^+$ Calcd for $C_{13}H_8F_3NNaO_2$ 290.0399; Found 290.0399.

2-Methoxy-9*H*-carbazole (10a)[3]

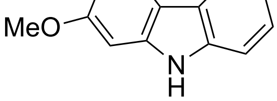

To an oven-dried microwave vial was added 1-bromo-2-nitrobenzene (1.00 g, 4.95 mmol), 4-methoxyphenylboronic acid (0.979 g, 6.44 mmol), palladium(II) diacetate (0.0220 g, 0.0990 mmol), triphenylphosphine (3.25 g, 12.4 mmol), potassium carbonate (1.37 g, 9.90 mmol) and *o*-dichlorobenzene (8 mL). The reaction vessel was sealed and heated under reflux for 24 h. The reaction

mixture was cooled to room temperature and applied directly to silica gel column chromatography (dichloromethane:hexane, 2:1) to give 2-methoxy-9*H*-carbazole (**10a**) as a white solid (0.875 g, 4.44 mmol, 90%). R_f = 0.34 (dichloromethane:hexane, 2:1); Mp 223–233 °C (lit.[3] 227–228 °C); ^{1}H NMR (400 MHz, $CDCl_3$) δ 7.97 (br d, J = 7.9 Hz, 1H, 5-H), 7.94 (d, J = 8.5 Hz, 1H, 4-H), 7.39 (dt, J = 9.1, 1.0 Hz, 1H, 8-H), 7.33 (ddd, J = 9.1, 6.3, 2.2 Hz, 1H, 7-H), 7.21 (ddd, J = 7.9, 6.3, 1.0 Hz, 1H, 6-H), 6.92 (d, J = 2.3 Hz, 1H, 1-H), 6.86 (dd, J = 8.5, 2.3 Hz, 1H, 3-H), 3.91 (s, 3H, OMe); ^{13}C NMR (101 MHz, $CDCl_3$) δ 159.3 (C), 141.0 (C), 139.7 (C), 124.7 (CH), 123.7 (C), 121.2 (CH), 119.7 (CH), 119.6 (CH), 117.4 (C), 110.4 (CH), 108.3 (CH), 94.9 (CH), 55.8 (CH_3); MS (ESI) *m/z* 220 (M + Na^+, 100).

7*H*-Benzo[c]carbazole (10b)[4]

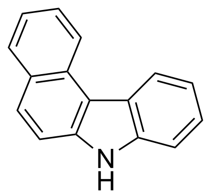

To a round bottomed flask fitted with a reflux condenser was added 1'-(2-nitrophenyl)naphthalene (1.02 g, 4.09 mmol), triphenylphosphine (2.68 g, 10.2 mmol) and *o*-dichlorobenzene (9 mL). The reaction mixture was heated under reflux for 24 h. The reaction mixture was cooled to room temperature and applied directly to silica gel column chromatography (hexane:diethyl ether, 4:1) to give 7*H*-benzo[c]carbazole (**10b**) as a white solid (0.663 g, 3.05 mmol, 75%). R_f = 0.14 (hexane:diethyl ether, 4:1); Mp 132–133 °C (lit.[4] 133–135 °C); ^{1}H NMR (400 MHz, $CDCl_3$) δ 8.82 (d, J = 8.3 Hz, 1H, 11-H), 8.61 (d, J = 8.2 Hz, 1H, 1-H), 8.24 (br s, 1H, NH), 8.04 (d, J = 8.2 Hz, 1H, 4-H), 7.86 (d, J = 8.4 Hz, 1H, 8-H), 7.76 (ddd, J = 8.4, 6.9, 1.3 Hz, 1H, 9-H), 7.55–7.40 (m, 5H, 2-H, 3-H, 5-H, 6-H and 10-H); ^{13}C NMR (101 MHz, $CDCl_3$) δ 138.5 (C), 137.1 (C), 130.0 (C), 129.32 (C), 129.29 (CH), 127.5 (CH), 127.0 (CH), 124.4 (CH), 124.0 (C), 123.4 (CH), 123.1 (CH), 122.1 (CH), 120.3 (CH), 115.5 (C), 112.7 (CH), 111.3 (CH); MS (ESI) *m/z* 240 (M + Na^+, 100).

2-Cyano-9*H*-carbazole (10c)[5]

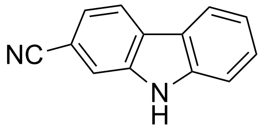

To a round bottomed flask fitted with a reflux condenser was added 2-nitro-[1,1'-biphenyl]-4'-carbonitrile (2.05 g, 9.12 mmol), triphenylphosphine (5.98 g, 22.8 mmol) and *o*-dichlorobenzene (18 mL). The reaction mixture was heated under reflux for 24 h. The reaction mixture was cooled to room temperature and applied directly to silica gel column chromatography (dichloromethane:hexane, 3:1) to give 2-cyano-9*H*-carbazole (**10c**) as a white solid (1.22 g, 6.34 mmol, 70%). R_f = 0.17

(dichloromethane:hexane, 3:1); Mp 115–117 °C (lit.[5] 120–122 °C); ^{1}H NMR (400 MHz, $CDCl_3$) δ 8.40 (1H, br s, NH), 8.15–8.08 (m, 2H, 4-H and 5-H), 7.77–7.74 (m, 1H, 1-H), 7.56–7.47 (m, 3H, 3-H, 7-H and 8-H), 7.31 (ddd, J = 8.0, 6.5, 1.6 Hz, 1H, 6-H); ^{13}C NMR (101 MHz, $CDCl_3$) δ 140.8 (C), 138.5 (C), 128.0 (CH), 127.0 (C), 122.8 (CH), 122.3 (C), 121.3 (CH), 121.1 (CH), 120.6 (CH), 120.3 (C), 115.0 (CH), 111.3 (CH), 108.2 (C); MS (ESI) *m/z* 215 (M + Na^+, 100).

2-Trifluoromethyl-9*H*-carbazole (10d)[4]

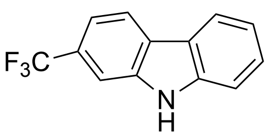

To a round bottomed flask fitted with a reflux condenser was added 2-nitro-4'-(trifluoromethyl)-1,1'-biphenyl (1.97 g, 7.38 mmol), triphenylphosphine (4.84 g, 18.5 mmol) and *o*-dichlorobenzene (15 mL). The reaction mixture was heated under reflux for 6.5 h. The reaction mixture was cooled to room temperature and applied directly to silica gel column chromatography (hexane:dichloromethane, 3:1) to give 2-trifluoromethyl-9*H*-carbazole (**10d**) as a white solid (1.25 g, 5.32 mmol, 72%). R_f = 0.17 (hexane:dichloromethane, 3:1); Mp 206–208 °C (lit.[4] 208–210 °C); ^{1}H NMR (400 MHz, $CDCl_3$) δ 8.24 (br s, 1H, NH), 8.16 (d, J = 7.9 Hz, 1H, 4-H), 8.12 (d, J = 8.2 Hz, 1H, 5-H), 7.70 (br s, 1H, 1-H), 7.53–7.46 (m, 3H, 3-H, 7-H and 8-H), 7.32–7.27 (m, 1H, 6-H); ^{13}C NMR (101 MHz, $CDCl_3$) δ 140.5 (C), 138.7 (C), 127.8 (q, $^{2}J_{C\text{-}F}$ = 30.8 Hz, C), 127.3 (CH), 126.1 (C), 125.5 (q, $^{1}J_{C\text{-}F}$ = 178.1 Hz, C), 122.6 (C), 121.1 (CH), 120.8 (CH), 120.3 (CH), 116.4 (q, $^{3}J_{C\text{-}F}$ = 3.7 Hz, CH), 111.1 (CH), 108.4 (q, $^{3}J_{C\text{-}F}$ = 4.3 Hz, CH); ^{19}F NMR (377 MHz, $CDCl_3$): δ = –61.0 (s); MS (EI) *m/z* 235 (M^+, 100), 216 (9), 173 (10).

2-Methoxy-6-bromo-9*H*-carbazole (11a)[6]

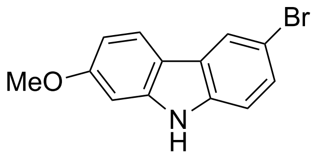

To a round bottomed flask was added 2-methoxy-9*H*-carbazole (**10a**) (875 mg, 4.44 mmol) and *N,N'*-dimethylformamide (10 mL). The solution was cooled to –5 °C in an ice/brine bath, and *N*-bromosuccinimide (790 mg, 4.44 mmol) was added. The reaction mixture was stirred for 2 h and quenched with a 5% aqueous lithium chloride solution (50 mL). The product was extracted with diethyl ether (2 × 50 mL). The organic layers were combined, dried over magnesium sulfate and concentrated under reduced pressure. Purification by recrystallisation from chloroform gave 2-methoxy-6-bromo-9*H*-carbazole (**11a**) as a colourless solid (976 mg, 3.53 mmol, 80%). Mp 216–218 °C (lit.[6] 218–220 °C); ^{1}H NMR (400 MHz, $CDCl_3$) δ 8.20 (br s, 1H, 5-H), 8.00 (br s, 1H, NH), 7.94 (d, J = 7.8 Hz, 1H, 4-H), 7.42–7.21 (m, 3H, 3-H, 7-H and 8-H), 6.95 (br s, 1H, 1-H), 3.98 (s, 3H, OMe); ^{13}C NMR (101

MHz, $CDCl_3$) δ 154.8 (C), 139.8 (C), 139.7 (C), 125.3 (CH), 124.8 (CH), 122.8 (C), 120.1 (CH), 119.8 (CH), 118.2 (C), 110.7 (CH), 103.8 (C), 94.4 (CH), 56.6 (CH_3); MS (ESI) *m/z* 298 (M + Na^+, 100).

10-Bromo-7*H*-benzo[c]carbazole (11b)

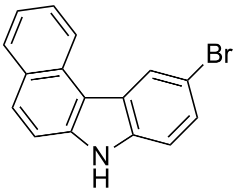

To a round bottomed flask was added 7*H*-benzo[c]carbazole (**10b**) (50 mg, 0.23 mmol) and *N,N'*-dimethylformamide (1 mL). The solution was cooled to –5 °C in an ice/brine bath, and *N*-bromosuccinimide (41 mg, 0.23 mmol) was added. The reaction mixture was stirred for 1 h and quenched with a 5% aqueous lithium chloride solution (30 mL). The product was extracted with diethyl ether (2 × 30 mL). The organic layers were combined, dried over magnesium sulfate and concentrated under reduced pressure. Purification by silica gel column chromatography (hexane:diethyl ether, 2:1) gave 10-bromo-7*H*-benzo[c]carbazole (**11b**) as a colourless solid (51 mg, 0.17 mmol, 75%). R_f = 0.13 (hexane:diethyl ether, 2:1); Mp 84–85 °C; IR (neat) 3413 (NH), 3046 (CH), 1533, 1470, 1293, 801, 742 cm^{-1}; ^1H NMR (400 MHz, $CDCl_3$) δ 8.64 (br s, 1H, 11-H), 8.62 (br d, *J* = 7.0 Hz, 1H, 1-H), 8.24 (br s, 1H, NH), 7.99 (d, *J* = 8.2 Hz, 1H, 4-H), 7.84 (d, *J* = 8.8 Hz, 1H, 6-H), 7.72 (ddd, *J* = 8.2, 7.0, 1.3 Hz, 1H, 3-H), 7.53–7.46 (m, 3H, 2-H, 5-H and 9-H), 7.32 (d, *J* = 8.6 Hz, 1H, 8-H); ^{13}C NMR (101 MHz, $CDCl_3$) δ 137.7 (C), 137.0 (C), 129.7 (C), 129.4 (CH), 129.3 (C), 128.3 (CH), 127.3 (CH), 127.1 (CH), 125.6 (C), 124.6 (CH), 123.5 (CH), 123.1 (CH), 114.6 (C), 113.2 (C), 112.55 (CH), 112.49 (CH); MS (ESI) *m/z* 318 (M + Na^+, 100); HRMS (ESI) *m/z*: $[M + Na]^+$ Calcd for $C_{16}H_{10}{}^{79}BrNNa$ 317.9889; Found 317.9884.

2-Cyano-6-bromo-9*H*-carbazole (11c)

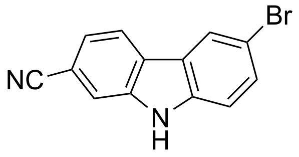

To a round bottomed flask was added 2-cyano-9*H*-carbazole (**10c**) (1.22 g, 6.34 mmol) and *N,N'*-dimethylformamide (20 mL). The solution was cooled to –5 °C in an ice/brine bath and *N*-bromosuccinimide (1.24 g, 6.98 mmol) was added. The reaction mixture was stirred for 6 h and quenched with a 5% aqueous lithium chloride solution (50 mL). The product was extracted with diethyl ether (2 × 50 mL). The organic layers were combined, dried over magnesium sulfate and concentrated under reduced pressure. Purification by silica gel column chromatography (hexane:diethyl ether, 1.5:1) gave 2-cyano-6-bromo-9*H*-carbazole (**11c**) as a colourless solid (1.59 g, 5.74 mmol, 91%). R_f = 0.10

(hexane:diethyl ether, 1.5:1); Mp 115–116 °C; IR (neat) 3289 (NH), 3067 (CH), 2970 (CH), 2224 (CN), 1738, 1476, 1271, 810 cm^{-1}; ^{1}H NMR (400 MHz, DMSO-d$_6$) δ 11.88 (s, 1H, NH), 8.52 (d, *J* = 1.9 Hz, 1H, 5-H), 8.38 (d, *J* = 8.1 Hz, 1H, 4-H), 8.01 (dd, *J* = 1.3, 0.7 Hz, 1H, 1-H), 7.62 (dd, *J* = 8.7, 1.9 Hz, 1H, 7-H), 7.56 (dd, *J* = 8.1, 1.3 Hz, 1H, 3-H), 7.55 (d, *J* = 8.7 Hz, 1H, 8-H); ^{13}C NMR (101 MHz, DMSO-d$_6$) δ 139.7 (C), 138.9 (C), 129.9 (CH), 124.9 (C), 123.9 (CH), 123.3 (C), 121.9 (CH), 121.8 (CH), 119.9 (C), 115.6 (CH), 113.6 (CH), 111.6 (C), 107.6 (C); MS (ESI) *m/z* 293 (M + Na$^+$, 100); HRMS (ESI) *m/z*: [M + Na]$^+$ Calcd for $C_{13}H_7{}^{79}BrN_2Na$ 292.9685; Found 292.9683.

2-Trifluoromethyl-6-bromo-9*H*-carbazole (11d)

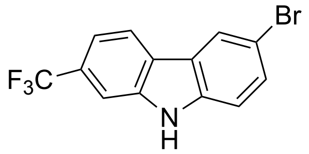

To a round bottomed flask was added 2-trifluoromethyl-9*H*-carbazole (**10d**) (1.15 g, 4.87 mmol) and *N,N'*-dimethylformamide (23 mL). The solution was cooled to –5 °C in an ice/brine bath and *N*-bromosuccinimide (0.866 g, 4.87 mmol) was added. The reaction mixture was stirred for 4.5 h and quenched with a 5% aqueous lithium chloride solution (50 mL). The product was extracted with diethyl ether (2 × 50 mL). The organic layers were combined, dried over magnesium sulfate and concentrated under reduced pressure. Purification by silica gel column chromatography (hexane:diethyl ether, 3:1) gave 2-trifluoromethyl-6-bromo-9*H*-carbazole (**11d**) as a colourless solid (1.23 g, 3.90 mmol, 80%). R$_f$ = 0.17 (hexane:diethyl ether, 3:1); Mp 112–113 °C; IR (neat) 3406 (NH), 1159, 1138, 904, 828, 730 cm^{-1}; ^{1}H NMR (400 MHz, CDCl$_3$) δ 8.22 (br s, 1H, NH), 8.21 (d, *J* = 1.9 Hz, 1H, 5-H), 8.08 (d, *J* = 8.2 Hz, 1H, 4-H), 7.68 (br s, 1H, 1-H), 7.57 (dd, *J* = 8.6, 1.9 Hz, 1H, 7-H), 7.49 (dd, *J* = 8.2, 0.8 Hz, 1H, 3-H), 7.35 (d, *J* = 8.6 Hz, 1H, 8-H); ^{13}C NMR (101 MHz, CDCl$_3$) δ 139.0 (C), 138.9 (C), 130.0 (CH), 128.5 (q, $^{2}J_{C\text{-}F}$ = 32.5 Hz, C), 125.0 (C), 124.8 (q, $^{1}J_{C\text{-}F}$ = 264.6 Hz, C), 124.3 (C), 123.8 (CH), 121.0 (CH), 116.8 (q, $^{3}J_{C\text{-}F}$ = 3.6 Hz, CH), 113.1 (C), 112.6 (CH), 108.3 (q, $^{3}J_{C\text{-}F}$ = 4.3 Hz, CH); ^{19}F NMR (377 MHz, CDCl$_3$) δ –61.1 (s); MS (ESI) *m/z* 314 (M + H$^+$, 100); HRMS (ESI) *m/z*: [M + H]$^+$ Calcd for $C_{13}H_8{}^{79}BrF_3N$ 313.9787; Found 313.9788.

Methyl (2*S*)-2-[(*tert*-butoxycarbonyl)amino]-3-(methylsulfonyloxy)propanoate[7]

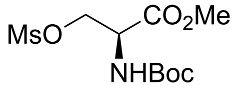

To a round bottomed flask was added methyl (2*S*)-2-[(*tert*-butoxycarbonyl)amino]-3-hydroxypropanoate (**7**) (5.00 g, 22.8 mmol) and dry dichloromethane (100 mL). The solution was cooled to 0 °C and diisopropylethylamine (4.37 mL, 25.1 mmol) and methanesulfonyl chloride (1.94 mL, 25.1 mmol) were added. The reaction mixture was stirred for 3.5 h at 0 °C. The reaction was

quenched with water (100 mL). The product was extracted with dichloromethane (2 × 100 mL) and washed with brine (100 mL). The solvent was removed under reduced pressure to give methyl (2*S*)-2-[(*tert*-butoxycarbonyl)amino]-3-(methylsulfonyloxy)propanoate as a colourless oil (6.43 g, 21.7 mmol, 95%). R_f = 0.25 (hexane:ethyl acetate, 1:1); $[\alpha]_D^{24}$ +28.2 (*c* 1.0, $CHCl_3$) [lit.[7] +22.0 (*c* 1.0, $CHCl_3$)]; ^{1}H NMR (500 MHz, $CDCl_3$) δ 5.45 (br d, *J* = 7.2 Hz, 1H, NH), 4.59–4.51 (m, 2H, 2-H and 3-H*H*), 4.46 (dd, *J* = 10.4, 3.4 Hz, 1H, 3-*H*H), 3.77 (s, 3H, OMe), 2.99 (s, 3H, SO_2Me), 1.41 (s, 9H, 3 × CH_3); ^{13}C NMR (101 MHz, $CDCl_3$) δ 169.2 (C), 155.1 (C), 80.7 (C), 69.0 (CH_2), 53.2 (CH), 53.1 (CH_3), 37.4 (CH_3), 28.3 (3 × CH_3); MS (ESI) *m/z* 320 (M + Na^+, 100).

Methyl (2*R*)-2-[(*tert*-butoxycarbonyl)amino]-3-iodopropanoate (8)[8]

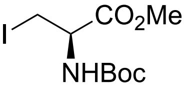

To a round bottomed flask was added methyl (2*S*)-2-[(*tert*-butoxycarbonyl)amino]-3-(methylsulfonyloxy)propanoate (6.77 g, 22.8 mmol) and acetone (150 mL). To the solution was added sodium iodide (6.83 g, 45.6 mmol). The reaction mixture was stirred under reflux for 5 h. The reaction mixture was filtered and the solvent was removed under reduced pressure to give methyl (2*R*)-2-[(*tert*-butoxycarbonyl)amino]-3-iodopropanoate (**8**) as a yellow solid (7.16 g, 21.8 mmol, 95%). R_f = 0.45 (hexane:ethyl acetate, 2:1); Mp 43–45 °C; $[\alpha]_D^{24}$ +37.4 (*c* 1.0, $CHCl_3$) [lit.[8] +40.3 (*c* 1.0, $CHCl_3$)]; ^{1}H NMR (400 MHz, $CDCl_3$) δ 5.35 (br d, *J* = 7.0 Hz, 1H, NH), 4.54–4.48 (m, 1H, 2-H), 3.79 (s, 3H, OMe), 3.58 (dd, *J* = 10.4, 3.8 Hz, 3-H*H*), 3.54 (dd, *J* = 10.4, 4.1 Hz, 1H, 3-*H*H), 1.45 (s, 9H, 3 × CH_3); ^{13}C NMR (101 MHz, $CDCl_3$) δ 170.2 (C), 155.0 (C), 80.6 (C), 53.8 (CH_3), 53.1 (CH), 28.4 (3 × CH_3), 7.9 (CH_2); MS (ESI) *m/z* 352 (M + Na^+, 100).

Methyl (2*S*)-2-[(*tert*-butoxycarbonyl)amino]-3-(9*H*-carbazol-3'-yl)propanoate (12a)

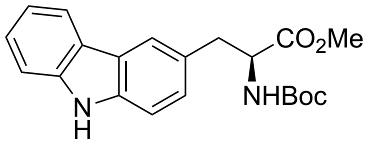

To an oven-dried, two-neck round bottomed flask under an inert atmosphere was added zinc dust (179 mg, 2.73 mmol), dry *N,N*'-dimethylformamide (1 mL) and iodine (35.0 mg, 0.137 mmol). Methyl (2*R*)-2-[(*tert*-butoxycarbonyl)amino]-3-iodopropanoate (**8**) (300 mg, 0.911 mmol) was added as a solution in *N,N*'-dimethylformamide (0.5 mL). A further portion of iodine was added (35.0 mg, 0.137 mmol) and a colour change from yellow to colourless was observed, along with a noticeable exotherm. The reaction mixture was stirred at room temperature for 1 h. Tris(dibenzylideneacetone)dipalladium(0) (21.0 mg, 0.0228 mmol) and 2-dicyclohexylphosphino-2′,6′-dimethoxybiphenyl (19.0 mg, 0.0456 mmol) and 3-iodo-9*H*-carbazole (347 mg, 1.18 mmol) were then added to the reaction mixture and

stirring was continued for 3 h. The reaction mixture was diluted with ethyl acetate (50 mL) and washed with an aqueous lithium chloride solution (5% w/w, 2 × 50 mL). The organic layer was dried over magnesium sulfate and concentrated under reduced pressure. Purification by silica gel column chromatography (hexane:ethyl acetate, 2:1) gave methyl (2*S*)-2-[(*tert*-butoxycarbonyl)amino]-3-(9*H*-carbazol-3'-yl)propanoate (**12a**) as a pale yellow solid (158 mg, 0.429 mmol, 47%). R_f = 0.25 (hexane:ethyl acetate, 2:1); Mp 136–137 °C; $[\alpha]_D^{24}$ +38.4 (*c* 0.1, $CHCl_3$); IR (neat) 3375 (NH), 2976 (CH), 1738 (CO), 1694 (CO), 1495, 1366, 1242, 1163 cm^{-1}; ^1H NMR (400 MHz, $CDCl_3$) δ 8.17 (br s, 1H, 9'-H), 8.03 (dd, *J* = 7.8, 0.7 Hz, 1H, 5'-H), 7.82 (br s, 1H, 4'-H), 7.43–7.39 (m, 2H, 2'-H and 7'-H), 7.32 (d, *J* = 8.2 Hz, 1H, 1'-H), 7.25–7.20 (m, 1H, 6'-H), 7.15 (dd, *J* = 8.2, 1.7 Hz, 1H, 8'-H), 5.04 (br d, *J* = 8.0 Hz, 1H, 2-NH), 4.62–4.59 (m, 1H, 2-H), 3.72 (s, 3H, OMe), 3.28 (dd, *J* = 13.9, 5.4 Hz, 1H, 3-H*H*), 3.23 (dd, *J* = 13.9, 6.2 Hz, 1H, 3-*H*H), 1.42 (s, 9H, 3 × CH_3); ^{13}C NMR (101 MHz, $CDCl_3$) δ 172.8 (C), 155.4 (C), 140.0 (C), 138.8 (C), 127.1 (CH), 126.8 (C), 126.1 (CH), 123.7 (C), 123.2 (C), 121.0 (CH), 120.3 (CH), 119.5 (CH), 110.83 (CH), 110.82 (CH), 80.1 (C), 55.2 (CH), 52.3 (CH_3), 38.5 (CH_2), 28.4 (3 × CH_3); MS (ESI) *m/z* 391 (M + Na^+, 100); HRMS (ESI) *m/z*: $[M + Na]^+$ Calcd for $C_{21}H_{24}N_2NaO_4$ 391.1628; Found 391.1627.

Methyl (2*S*)-2-[(*tert*-butoxycarbonyl)amino]-3-(7-methoxy-9*H*-carbazol-3'-yl)propanoate (12b)

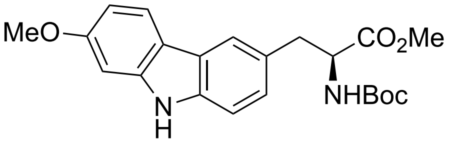

To an oven-dried, two-neck round bottomed flask under an inert atmosphere was added zinc dust (147 mg, 2.25 mmol), dry *N,N*'-dimethylformamide (0.8 mL) and iodine (29.0 mg, 0.115 mmol). Methyl (2*R*)-2-[(*tert*-butoxycarbonyl)amino]-3-iodopropanoate (**8**) (250 mg, 0.763 mmol) was added as a solution in *N,N*'-dimethylformamide (0.4 mL). A further portion of iodine was added (29.0 mg, 0.115 mmol) and a colour change from yellow to colourless was observed, along with a noticeable exotherm. The reaction mixture was stirred at room temperature for 1 h. Tris(dibenzylideneacetone)dipalladium(0) (17.0 mg, 0.0191 mmol) and 2-dicyclohexylphosphino-2′,6′-dimethoxybiphenyl (16.0 mg, 0.0382 mmol) and 2-methoxy-6-bromo-9*H*-carbazole (**11a**) (274 mg, 0.992 mmol) were then added to the reaction mixture. The reaction mixture was heated to 50 °C and stirred for 3 h. The reaction mixture was diluted with ethyl acetate (50 mL) and washed with an aqueous lithium chloride solution (5% w/w, 50 mL). The organic layer was dried over magnesium sulfate and concentrated under reduced pressure. Purification by silica gel column chromatography (hexane:ethyl acetate, 2:1) gave methyl (2*S*)-2-[(*tert*-butoxycarbonyl)amino]-3-(7-methoxy-9*H*-carbazol-3'-yl)propanoate (**12b**) as a colourless solid (88.0 mg, 0.239 mmol, 31%). R_f = 0.12 (hexane:ethyl acetate, 2:1); Mp 63–64 °C; $[\alpha]_D^{13}$ +19.0 (*c* 0.1, $CHCl_3$); IR (neat) 3381 (NH), 2978 (CH), 1738 (CO), 1694 (CO), 1497, 1456, 1366, 1310, 1196, 1153, 1138,

1036, 733 cm^{-1}; ^{1}H NMR (400 MHz, $CDCl_3$) δ 8.41 (br s, 1H, 9'-H), 7.93 (d, J = 7.7 Hz, 1H, 5'-H), 7.73 (br s, 1H, 4'-H), 7.38–7.30 (m, 2H, 1'-H and 2'-H), 7.22–7.17 (m, 1H, 6'-H), 6.67 (br s, 1H, 8'-H), 5.33 (br d, J = 7.7 Hz, 1H, 2-NH), 4.67–4.59 (m, 1H, 2-H), 3.71 (s, 3H, OMe), 3.66 (s, 3H, OMe), 3.26 (dd, J 13.4, 5.8 Hz, 1H, 3-H*H*), 3.17 (dd, J = 13.4, 7.7 Hz, 1H, 3-*H*H), 1.40 (s, 9H, 3 × CH_3); ^{13}C NMR (101 MHz, $CDCl_3$) δ 173.4 (C), 157.1 (C), 155.6 (C), 140.3 (C), 139.6 (C), 124.5 (CH), 123.3 (C), 122.4 (CH), 119.42 (CH), 119.36 (CH), 117.0 (C), 116.5 (C), 110.6 (CH), 93.0 (CH), 79.9 (C), 55.4 (CH_3), 54.6 (CH), 52.1 (CH_3), 33.7 (CH_2), 28.4 (3 × CH_3); MS (ESI) *m/z* 421 (M + Na^+, 100); HRMS (ESI) *m/z*: $[M + Na]^+$ Calcd for $C_{22}H_{26}N_2NaO_5$ 421.1734; Found 421.1733.

Methyl (2*S*)-3-(7*H*-benzo[c]carbazol-10-yl)-2-[(*tert*-butoxycarbonyl)amino]propanoate (12c)

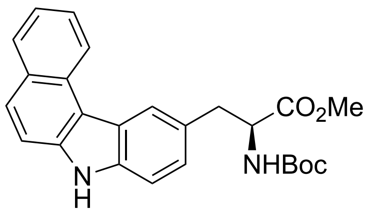

To an oven-dried, two-neck round bottomed flask under an inert atmosphere was added zinc dust (208 mg, 3.18 mmol), dry *N,N*'-dimethylformamide (4 mL) and a solution of iodine in dimethylformamide (0.250 mL, 0.640 M, 0.159 mmol). Methyl (2*R*)-2-[(*tert*-butoxycarbonyl)amino]-3-iodopropanoate (**8**) (350 mg, 1.06 mmol) was added as a solution in *N,N*'-dimethylformamide (0.5 mL). A further portion of iodine in *N,N*'-dimethylformamide was added (0.250 mL, 0.640 M, 0.159 mmol) and a colour change from yellow to colourless was observed, along with a noticeable exotherm. The reaction mixture was stirred at room temperature for 1 h. Tris(dibenzylideneacetone)dipalladium(0) (27.0 mg, 0.0265 mmol) and 2-dicyclohexylphosphino-2′,6′-dimethoxybiphenyl (22.0 mg, 0.0532 mmol) and 10-bromo-7*H*-benzo[c]carbazole (**11b**) (410 mg, 1.38 mmol) were added to the reaction mixture. The reaction mixture was heated to 50 °C and stirred for 22 h. The reaction mixture was cooled to room temperature and diluted with ethyl acetate (30 mL) and washed with an aqueous lithium chloride solution (5% w/w, 30 mL). The organic layer was dried over magnesium sulfate and concentrated under reduced pressure. Purification by silica gel column chromatography (hexane:diethyl ether, 1:1) gave methyl (2*S*)-3-(7*H*-benzo[c]carbazol-10-yl)-2-[(*tert*-butoxycarbonyl)amino]propanoate (**12c**) as a colourless solid (193 mg, 0.46 mmol, 44%). R_f = 0.12 (hexane:diethyl ether, 1:1); Mp 151–152 °C; $[\alpha]_D^{14}$ +53.8 (*c* 0.1, $CHCl_3$); IR (neat) 3365 (NH), 2977 (CH), 1689 (CO), 1502, 1483, 1366, 1164, 753 cm^{-1}; ^{1}H NMR (400 MHz, $CDCl_3$) δ 8.71 (d, J = 8.3 Hz, 1H, 1'-H), 8.58 (br s, 1H, 7'-H), 8.27 (br s, 1H, 11'-H), 7.98 (d, J = 7.9 Hz, 1H, 4'-H), 7.81 (d, J = 8.8 Hz, 1H, 5'-H), 7.73–7.68 (m, 1H, 3'-H), 7.56 (d, J = 8.8 Hz, 1H, 6'-H), 7.51–7.45 (m, 1H, 2'-H), 7.41 (d, J = 8.3 Hz, 1H, 9'-H), 7.16 (d, J = 8.3 Hz, 1H, 8'-H), 5.14 (br d, J = 8.1 Hz, 1H, 2-NH), 4.78–4.71 (m, 1H, 2-H), 3.75 (s, 3H, OMe), 3.39 (dd, J = 13.5, 5.3 Hz, 1H, 3-H*H*), 3.34 (dd, J = 13.5, 5.5 Hz, 1H, 3-*H*H), 1.43 (s, 9H, 3 × CH_3); ^{13}C NMR (101 MHz, $CDCl_3$) δ

172.8 (C), 155.4 (C), 137.7 (C), 137.6 (C), 130.0 (C), 129.4 (C), 129.3 (CH), 127.6 (CH), 127.5 (C), 127.0 (CH), 125.5 (CH), 124.3 (C), 123.2 (CH), 123.1 (CH), 122.7 (CH), 115.1 (C), 112.8 (CH), 111.4 (CH), 80.1 (C), 55.2 (CH), 52.4 (CH_3), 38.8 (CH_2), 28.5 (3 × CH_3); MS (ESI) *m/z* 441 (M + Na^+, 100); HRMS (ESI) *m/z*: $[M + Na]^+$ Calcd for $C_{25}H_{26}N_2NaO_4$ 441.1785; Found 441.1778.

Methyl (2*S*)-2-[(*tert*-butoxycarbonyl)amino]-3-(7-cyano-9*H*-carbazol-3'-yl)propanoate (12d)

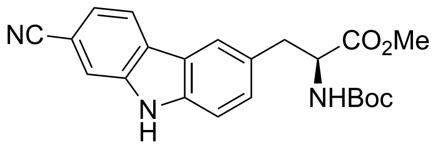

To an oven-dried, two-neck round bottomed flask under an inert atmosphere was added zinc dust (149 mg, 2.28 mmol), dry *N,N*'-dimethylformamide (0.8 mL) and iodine (29.0 mg, 0.115 mmol). Methyl (2*R*)-2-[(*tert*-butoxycarbonyl)amino]-3-iodopropanoate (**8**) (250 mg, 0.759 mmol) was added as a solution in *N,N*'-dimethylformamide (0.4 mL). A further portion of iodine was added (29.0 mg, 0.115 mmol) and a colour change from yellow to colourless was observed, along with a noticeable exotherm. The reaction mixture was stirred at room temperature for 1 h. Tris(dibenzylideneacetone)dipalladium(0) (16.0 mg, 0.0190 mmol) and 2-dicyclohexylphosphino-2′,6′-dimethoxybiphenyl (16.0 mg, 0.0380 mmol) and 2-cyano-6-bromo-9*H*-carbazole (**11c**) (268 mg, 0.987 mmol) were added to the reaction mixture. The reaction mixture was heated to 50 °C and stirred for 24 h. The reaction mixture was cooled to room temperature and then applied directly to silica gel column chromatography (hexane:diethyl ether, 1:1) to give methyl (2*S*)-2-[(*tert*-butoxycarbonyl)amino]-3-(7-cyano-9*H*-carbazol-3'-yl)propanoate (**12d**) as a colourless solid (121 mg, 0.308 mmol, 41%). R_f = 0.06 (hexane:diethyl ether, 1:1); Mp 87–88 °C; $[\alpha]_D^{13}$ +52.0 (*c* 0.1, $CHCl_3$); IR (neat) 3339 (NH), 2977 (CH), 2221 (CN), 1695 (CO), 1494, 1366, 1250, 1165 cm^{-1}; ^1H NMR (400 MHz, $CDCl_3$) δ 8.96 (br s, 1H, 9'-H), 7.94 (d, *J* = 8.1 Hz, 1H, 5'-H), 7.79 (s, 1H, 4'-H), 7.67 (s, 1H, 8'-H), 7.39 (d, *J* = 8.1 Hz, 1H, 6'-H), 7.29 (d, *J* = 8.3 Hz, 1H, 1'-H), 7.20 (dd, *J* = 8.3, 1.6 Hz, 1H, 2'-H), 5.15 (br d, *J* = 8.2 Hz, 1H, 2-NH), 4.71–4.63 (m, 1H, 2-H), 3.76 (s, 3H, OMe), 3.29 (dd, *J* = 13.9, 5.6 Hz, 1H, 3-H*H*), 3.20 (dd, *J* = 13.9, 6.4 Hz, 1H, 3-*H*H), 1.41 (s, 9H, 3 × CH_3); ^{13}C NMR (101 MHz, $CDCl_3$) δ 172.8 (C), 155.4 (C), 139.9 (C), 138.8 (C), 128.9 (CH), 127.8 (C), 126.3 (C), 122.4 (CH), 122.3 (C), 121.5 (CH), 120.8 (CH), 120.4 (C), 115.0 (CH), 111.5 (CH), 107.9 (C), 80.4 (C), 55.2 (CH), 52.5 (CH_3), 38.6 (CH_2), 28.4 (3 × CH_3); MS (ESI) *m/z* 416 (M + Na^+, 100); HRMS (ESI) *m/z*: $[M + Na]^+$ Calcd for $C_{22}H_{23}N_3NaO_4$ 416.1581; Found 416.1577.

Methyl (2*S*)-2-[(*tert*-butoxycarbonyl)amino]-3-(7-trifluoromethyl-9*H*-carbazol-3-yl)propanoate (12e)

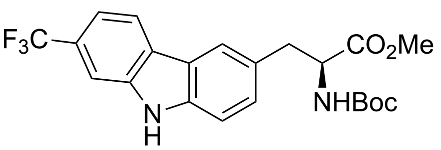

To an oven-dried, two-neck round bottomed flask under an inert atmosphere was added zinc dust (208 mg, 3.18 mmol), dry *N,N*'-dimethylformamide (4 mL) and a solution of iodine (40.5 mg, 0.159 mmol) in *N,N*'-dimethylformamide (0.25 mL). Methyl (2*R*)-2-[(*tert*-butoxycarbonyl)amino]-3-iodopropanoate (**8**) (350 mg, 1.06 mmol) was added as a solution in *N,N*'-dimethylformamide (0.5 mL). A further solution of iodine (40.5 mg, 0.159 mmol) in *N,N*'-dimethylformamide (0.25 mL) was added, and a colour change from yellow to colourless was observed, along with a noticeable exotherm. The reaction mixture was stirred at room temperature for 1 h. Tris(dibenzylideneacetone)dipalladium(0) (27.0 mg, 0.0265 mmol) and 2-dicyclohexylphosphino-2′,6′-dimethoxybiphenyl (22.0 mg, 0.053 mmol) and 2-trifluoromethyl-6-bromo-9*H*-carbazole (**11d**) (433 mg, 1.38 mmol) were added to the reaction mixture. The reaction mixture was heated to 50 °C and stirred for 22 h. The reaction mixture was cooled to room temperature and then applied directly to silica gel column chromatography (hexane:ethyl acetate, 3:1) to give methyl (2*S*)-2-[(*tert*-butoxycarbonyl)amino]-3-(7-trifluoromethyl-9*H*-carbazol-3-yl)propanoate (**12e**) as a colourless solid (224 mg, 0.514 mmol, 48%). R_f = 0.15 (hexane:ethyl acetate, 3:1); Mp 131–133 °C; $[\alpha]_D^{13}$ +47.2 (*c* 0.1, $CHCl_3$); IR (neat) 3343 (NH), 2979 (CH), 1694 (CO), 1490, 1332, 1157, 1115, 1055, 754 cm^{-1}; ^{1}H NMR (400 MHz, $CDCl_3$) δ 8.59 (br s, 1H, 9'-H), 7.98 (d, J = 8.2 Hz, 1H, 5'-H), 7.80 (s, 1H, 4'-H), 7.64 (s, 1H, 8'-H), 7.40 (d, J = 8.2 Hz, 1H, 6'-H), 7.26 (d, J = 8.3 Hz, 1H, 2'-H), 7.18 (d, J = 8.3 Hz, 1H, 1'-H), 5.10 (br d, J = 8.0 Hz, 1H, 2-NH), 4.72–4.64 (m, 1H, 2-H), 3.75 (s, 3H, OMe), 3.28 (dd, J = 13.8, 5.7 Hz, 1H, 3-H*H*), 3.21 (dd, J = 13.8, 6.1 Hz, 1H, 3-*H*H), 1.42 (s, 9H, 3 × CH_3); ^{13}C NMR (101 MHz, $CDCl_3$) δ 172.8 (C), 155.5 (C), 139.7 (C), 139.0 (C), 128.3 (CH), 127.7 (q, $^2J_{C\text{-}F}$ = 32.1 Hz, C), 127.5 (C), 125.5 (C), 125.0 (q, $^1J_{C\text{-}F}$ = 271.9 Hz, C), 122.7 (C), 121.4 (CH), 120.5 (CH), 116.1 (q, $^3J_{C\text{-}F}$ = 3.7 Hz CH), 111.3 (CH), 108.1 (q, $^3J_{C\text{-}F}$ = 4.4 Hz, CH), 80.3 (C), 55.2 (CH), 52.4 (CH_3), 38.6 (CH_2), 28.4 (3 × CH_3); ^{19}F NMR (377 MHz, $CDCl_3$) δ −60.9 (s); MS (ESI) *m/z* 459 (M + Na^+, 100); HRMS (ESI) *m/z*: $[M + Na]^+$ Calcd for $C_{22}H_{23}F_3N_2NaO_4$ 459.1502; Found 459.1505.

(2*S*)-2-Amino-3-(9*H*-carbazol-3'-yl)propanoic acid hydrochloride (13a)

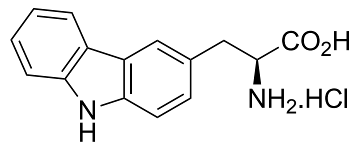

To a round bottomed flask was added methyl (2*S*)-2-[(*tert*-butoxycarbonyl)amino]-3-(9*H*-carbazol-3'-yl)propanoate (**12a**) (158 mg, 0.430 mmol), methanol (8 mL) and chloroform (2 mL). A solution of lithium hydroxide monohydrate (45.0 mg, 1.07 mmol) in water (0.5 mL) was added dropwise. The reaction mixture was stirred at room temperature for 24 h. The solvent was removed under reduced pressure and the resulting residue redissolved in 1 M hydrochloric acid (10 mL). The product was extracted with ethyl acetate (2 × 20 mL). The organic layers were combined, dried over magnesium sulfate and concentrated under reduced pressure. The resulting residue was redissolved in acetonitrile (1 mL) and water (3.5 mL) and cooled to 0 °C. To the solution was added 6 M hydrochloric acid (3.5 mL) and the reaction mixture stirred for 1.75 h at 0 °C. The solvent was removed under reduced pressure to give (2*S*)-2-amino-3-(9*H*-carbazol-3'-yl)propanoic acid hydrochloride (**13a**) as a pale yellow solid (121 mg, 0.409 mmol, 95%). Mp 231 °C (decomp.); $[\alpha]_D^{13}$ –10.8 (*c* 0.1, MeOH); IR (neat) 3418 (NH), 3323 (OH), 2928 (CH), 1736 (CO), 1492, 1452, 1244, 1211, 768 cm^{-1}; ^1H NMR (400 MHz, CD_3OD) δ 8.05 (br d, J = 7.6 Hz, 1H, 5'-H), 8.01 (d, J = 1.4 Hz, 1H, 4'-H), 7.50–7.44 (m, 2H, 1'-H and 8'-H), 7.37 (td, J = 7.6, 1.0 Hz, 1H, 7'-H), 7.31 (dd, J = 8.0, 1.4 Hz, 1H, 2'-H), 7.15 (td, J = 7.6, 0.8 Hz, 1H, 6'-H), 4.35–4.28 (m, 1H, 2-H), 3.50 (dd, J = 14.8, 5.9 Hz, 1H, 3-H*H*), 3.32 (dd, J = 14.8, 5.9 Hz, 1H, 3-*H*H); ^{13}C NMR (101 MHz, CD_3OD) δ 171.5 (C), 141.9 (C), 141.0 (C), 127.7 (CH), 126.8 (CH), 125.2 (C), 124.9 (C), 123.9 (C), 121.9 (CH), 121.0 (CH), 119.8 (CH), 112.4 (CH), 111.9 (CH), 55.8 (CH), 37.4 (CH_2); MS (ESI) *m/z* 277 (M + Na^+, 100); HRMS (ESI) *m/z*: $[M + Na]^+$ Calcd for $C_{15}H_{14}N_2NaO_2$ 277.0947; Found 277.0944.

(2*S*)-2-Amino-3-(7-methoxy-9*H*-carbazol-3'-yl)propanoic acid hydrochloride (13b)

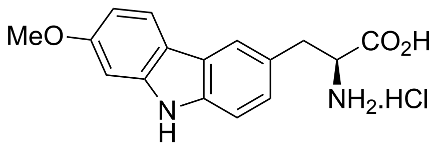

To a round bottomed flask was added methyl (2*S*)-2-[(*tert*-butoxycarbonyl)amino]-3-(7-methoxy-9*H*-carbazol-3'-yl)propanoate (**12b**) (108 mg, 0.271 mmol), methanol (5 mL) and chloroform (1 mL). A solution of lithium hydroxide monohydrate (28.0 mg, 0.678 mmol) in water (0.5 mL) was then added dropwise. The reaction mixture was stirred at room temperature for 24 h. The solvent was removed under reduced pressure and the resulting residue redissolved in 1 M hydrochloric acid (10 mL). The product was extracted with ethyl acetate (2 × 20 mL). The organic layers were combined, dried over magnesium sulfate and concentrated under reduced pressure. The resulting residue was redissolved in

acetonitrile (2 mL) and cooled to 0 °C. To the solution was added 6 M hydrochloric acid (2 mL) and the reaction mixture stirred for 6.5 h at 0 °C. The solvent was removed under reduced pressure to give (2*S*)-2-amino-3-(7-methoxy-9*H*-carbazol-3'-yl)propanoic acid hydrochloride (**13b**) as a colourless solid (75.0 mg, 0.234 mmol, 86%). Mp 222 °C (decomp.); $[\alpha]_D^{14}$ +35.4 (*c* 0.1, MeOH); IR (neat) 3406 (NH), 2940 (CH), 1737 (CO), 1635, 1610, 1494, 1483, 1234, 1142, 750 cm^{-1}; ^1H NMR (400 MHz, CD_3OD) δ 7.93 (d, J = 7.6 Hz, 1H, 5'-H), 7.85 (s, 1H, 4'-H), 7.41 (d, J = 7.7 Hz, 1H, 1'-H), 7.31–7.25 (m, 1H, 2'-H), 7.14–7.09 (m, 1H, 6'-H), 7.07 (s, 1H, 8'-H), 4.32–4.25 (m, 1H, 2-H), 3.94 (s, 3H, OMe), 3.54 (dd, J = 14.5, 5.9 Hz, 1H, 3-H*H*), 3.21 (dd, J = 14.5, 8.0 Hz, 1H, 3-*H*H); ^{13}C NMR (101 MHz, CD_3OD) δ 171.7 (C), 158.3 (C), 142.6 (C), 141.4 (C), 125.4 (CH), 124.2 (C), 123.6 (CH), 120.1 (CH), 119.9 (CH), 117.7 (C), 115.2 (C), 111.6 (CH), 94.2 (CH), 56.0 (CH_3), 54.7 (CH), 33.4 (CH_2); MS (ESI) *m/z* 285 (M + H^+, 100); HRMS (ESI) *m/z*: $[M + H]^+$ Calcd for $C_{16}H_{17}N_2O_3$ 285.1234; Found 285.1237.

(2*S*)-2-Amino-3-(7*H*-benzo[c]carbazol-10-yl)propanoic acid hydrochloride (13c)

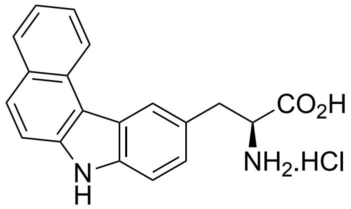

To a round bottomed flask was added methyl (2*S*)-3-(7*H*-benzo[c]carbazol-10-yl)-2-[(*tert*-butoxycarbonyl)amino]propanoate (**12c**) (86.0 mg, 0.206 mmol), methanol (4 mL) and acetone (0.5 mL). A solution of lithium hydroxide monohydrate (22.0 mg, 0.515 mmol) in water (0.7 mL) was added dropwise. The reaction mixture was stirred at room temperature for 22 h. The solvent was removed under reduced pressure and the resulting residue redissolved in 1 M hydrochloric acid (10 mL). The product was extracted with ethyl acetate (2 × 20 mL). The organic layers were combined, dried over magnesium sulfate and concentrated under reduced pressure. The resulting residue was redissolved in acetonitrile (1.4 mL) and cooled to 0 °C. To the solution was added 6 M hydrochloric acid (1.4 mL) and the reaction mixture stirred for 4 h at 0 °C. The solvent was then removed under reduced pressure to give (2*S*)-2-amino-3-(7*H*-benzo[c]carbazol-10-yl)propanoic acid hydrochloride (**13c**) as a colourless solid (66.5 mg, 0.196 mmol, 95%). Mp 222 °C (decomp.); $[\alpha]_D^{15}$ +14.8 (*c* 0.1, MeOH); IR (neat) 3400 (NH), 2920 (CH), 1738 (CO), 1482, 1324, 805 cm^{-1}; ^1H NMR (400 MHz, CD_3OD) δ 8.77 (d, J = 8.2 Hz, 1H, 1'-H), 8.47 (s, 1H, 11'-H), 7.96 (d, J = 8.0 Hz, 1H, 4'-H), 7.82 (d, J = 8.7 Hz, 1H, 6'-H), 7.71–7.65 (m, 2H, 3'-H and 5'-H), 7.61 (d, J = 8.2 Hz, 1H, 8'-H), 7.45–7.39 (m, 1H, 2'-H), 7.34 (d, J = 8.2 Hz, 1H, 9'-H), 4.42–4.35 (m, 1H, 2-H), 3.57 (dd, J = 14.5, 4.7 Hz, 1H, 3-H*H*), 3.42 (dd, J = 14.5, 7.5 Hz, 1H, 3-*H*H); ^{13}C NMR (101 MHz, CD_3OD) δ 171.5 (C), 139.9 (C), 139.5 (C), 131.2 (C), 130.5 (C), 130.2 (CH), 128.4 (CH), 127.7 (CH), 126.1 (CH), 125.4 (C), 125.0 (C), 124.1 (CH), 123.7 (2 × CH),

115.6 (C), 114.1 (CH), 113.0 (CH), 55.8 (CH), 37.8 (CH_2); MS (ESI) *m/z* 305 (M + H^+, 100); HRMS (ESI) *m/z*: $[M + H]^+$ Calcd for $C_{19}H_{17}N_2O_2$ 305.1285; Found 305.1286.

(2*S*)-2-Amino-3-(7-cyano-9*H*-carbazol-3'-yl)propanoic acid hydrochloride (13d)

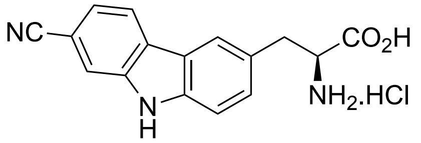

To a round bottomed flask was added methyl (2*S*)-2-[(*tert*-butoxycarbonyl)amino]-3-(7-cyano-9*H*-carbazol-3'-yl)propanoate (**12d**) (61.0 mg, 0.155 mmol), methanol (3 mL) and chloroform (0.5 mL). A solution of lithium hydroxide monohydrate (16.0 mg, 0.388 mmol) in water (0.5 mL) was added dropwise. The reaction mixture was stirred at room temperature for 24 h. The solvent was removed under reduced pressure and the resulting residue redissolved in 1 M hydrochloric acid (10 mL). The product was extracted with ethyl acetate (2 × 20 mL). The organic layers were combined, dried over magnesium sulfate and concentrated under reduced pressure. The resulting residue was redissolved in acetonitrile (1 mL) and cooled to 0 °C. To the solution was added 6 M hydrochloric acid (1 mL) and the reaction mixture stirred for 2 h at 0 °C. The solvent was then removed under reduced pressure to give (2*S*)-2-amino-3-(7-cyano-9*H*-carbazol-3'-yl)propanoic acid hydrochloride (**13d**) as a yellow solid (34.0 mg, 0.108 mmol, 69%). Mp 214 °C (decomp.); $[\alpha]_D^{14}$ –17.6 (*c* 0.1, MeOH); IR (neat) 2925 (CH), 2223 (CN), 1731 (CO), 1491, 1338, 1226, 813 cm^{-1}; ^1H NMR (400 MHz, DMSO-d_6) δ 11.90 (s, 1H, 9'-H), 8.24 (d, *J* = 8.2 Hz, 1H, 5'-H), 8.13 (br s, 1H, 4'-H), 7.99 (s, 1H, 8'-H), 7.56–7.52 (m, 2H, 1'-H and 6'-H), 7.42 (dd, *J* = 8.4, 1.3 Hz, 1H, 2'-H), 4.19 (t, *J* = 6.4 Hz, 1H, 2-H), 3.33 (dd, *J* = 14.2, 6.4 Hz, 1H, 3-H*H*), 3.28 (dd, *J* = 14.2, 6.4 Hz, 1H, 3-*H*H); ^{13}C NMR (101 MHz, DMSO-d_6) δ 170.5 (C), 140.3 (C), 138.9 (C), 129.2 (CH), 125.9 (C), 125.7 (C), 122.1 (CH), 121.6 (CH), 121.5 (C), 121.2 (CH), 120.1 (C), 115.4 (CH), 111.8 (CH), 106.9 (C), 53.7 (CH), 35.9 (CH_2); MS (ESI) *m/z* 280 (M + H^+, 100); HRMS (ESI) *m/z*: $[M + H]^+$ Calcd for $C_{16}H_{14}N_3O_2$ 280.1081; Found 280.1079.

(2*S*)-2-Amino-3-(7-trifluoromethyl-9*H*-carbazol-3'-yl)propanoic acid hydrochloride (13e)

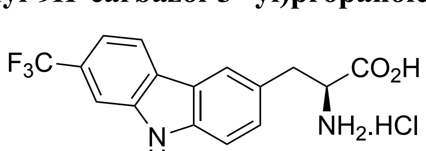

To a round bottomed flask was added methyl (2*S*)-2-[(*tert*-butoxycarbonyl)amino]-3-(7-(trifluoromethyl)-9*H*-carbazol-3'-yl)propanoate (**12e**) (112 mg, 0.257 mmol), methanol (5 mL) and acetone (1 mL). A solution of lithium hydroxide monohydrate (27.0 mg, 0.642 mmol) in water (0.5 mL) was added dropwise. The reaction mixture was stirred at room temperature for 19.5 h. The solvent was removed under reduced pressure and the resulting residue redissolved in 1 M hydrochloric acid (10

mL). The product was extracted with ethyl acetate (2 × 20 mL). The organic layers were combined, dried over magnesium sulfate and concentrated under reduced pressure. The resulting residue was redissolved in acetonitrile (2 mL) and cooled to 0 °C. To the solution was added 6 M hydrochloric acid (2 mL). The reaction mixture was stirred for 1 h at 0 °C and then allowed to reach room temperature for 1 h. The solvent was removed under reduced pressure to give (2*S*)-2-amino-3-(7-trifluoromethyl-9*H*-carbazol-3'-yl)propanoic acid hydrochloride (**13e**) as a colourless solid (86.0 mg, 0.240 mmol, 93%). Mp 186 °C (decomp.); $[\alpha]_D^{14}$ –24.4 (*c* 0.1, MeOH); IR (neat) 2926 (CH), 1728 (CO), 1488, 1438, 1330, 1252, 1111, 1055, 818 cm^{-1}; ^{1}H NMR (400 MHz, CD_3OD) δ 8.21 (d, *J* = 8.1 Hz, 1H, 5'-H), 8.09 (s, 1H, 4'-H), 7.76 (s, 1H, 8'-H), 7.54 (d, *J* = 8.1 Hz, 1H, 1'-H), 7.42 (m, 2H, 2'-H and 6'-H), 4.36–4.29 (m, 1H, 2-H), 3.52 (dd, *J* = 14.4, 3.7 Hz, 1H, 3-H*H*), 3.35 (dd, *J* = 14.4, 7.4 Hz, 1H, 3-*H*H); ^{13}C NMR (101 MHz, CD_3OD) δ 171.5 (C), 142.1 (C), 140.9 (C), 129.2 (CH), 128.5 (q, $^2J_{C\text{-}F}$ = 31.8 Hz, C), 126.7 (C), 126.5 (q, $^1J_{C\text{-}F}$ = 270.7 Hz, C), 126.3 (C), 123.9 (C), 122.7 (CH), 121.7 (CH), 116.2 (q, $^3J_{C\text{-}F}$ = 3.7 Hz, CH), 112.9 (CH), 109.1 (q, $^3J_{C\text{-}F}$ = 4.4 Hz, CH), 55.8 (CH), 37.5 (CH_2); ^{19}F NMR (377 MHz, CD_3OD) δ −62.4 (s); MS (ESI) *m/z* 323 (M + H^+, 100); HRMS (ESI) *m/z*: $[M + H]^+$ Calcd for $C_{16}H_{14}F_3N_2O_2$ 323.1002; Found 323.1008.

Methyl (2*S*)-2-[(9*H*-fluoren-9-ylmethoxycarbonyl)amino]-3-(7-trifluoromethyl-9*H*-carbazol-3-yl)propanoic acid (S1)

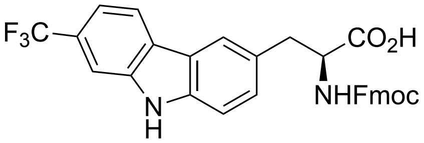

To a round bottomed flask was added (2*S*)-2-amino-3-(7-trifluoromethyl-9*H*-carbazol-3'-yl)propanoic acid hydrochloride (**13e**) (86.0 mg, 0.240 mmol), acetone (1 mL) and water (1 mL). Sodium bicarbonate (81.0 mg, 0.959 mmol) and *N*-(9-fluorenylmethoxycarbonyloxy)succinimide (79.0 mg, 0.235 mmol) were added. The reaction mixture was stirred at room temperature for 24 h. The solvent was removed under reduced pressure. The resulting residue was redissolved in ethyl acetate (10 mL) and acidified with 1 M hydrochloric acid to pH 2–3. The product was extracted with ethyl acetate (2 × 10 mL). The organic layers were combined, dried over magnesium sulfate and concentrated under reduced pressure to give methyl (2*S*)-2-[(9*H*-fluoren-9-ylmethoxycarbonyl)amino]-3-(7-(trifluoromethyl)-9*H*-carbazol-3-yl)propanoic acid (**S1**) as a colourless solid (83.0 mg, 0.152 mmol, 63%). R_f = 0.25 (acetone:hexane:acetic acid, 1:1:0.01); Mp 184–186 °C; $[\alpha]_D^{21}$ +27.0 (*c* 0.1, $CHCl_3$); IR (neat) 3372 (NH), 2859 (CH), 1703 (CO), 1452, 1252, 1120, 979 cm^{-1}; ^{1}H NMR (400 MHz, CD_3OD) δ 8.08 (d, *J* = 8.2 Hz, 1H, 5'-H), 8.00 (s, 1H, 4'-H), 7.69–7.64 (m, 3H, 8'-H and 2 × ArH), 7.47–7.24 (m, 7H, 1'-H, 2'-H, 6'-H and 4 × ArH), 7.11–7.06 (m, 2H, 2 × ArH), 4.54 (dd, *J* = 9.6, 4.8 Hz, 1H, 2-H), 4.24 (1H, dd, *J* = 10.4, 7.0 Hz, 1H, OCH*H*), 4.09 (dd, *J* = 10.4, 7.0 Hz, 1H, OC*H*H), 4.01 (t, *J* = 7.0 Hz, 1H,

OCH$_2$C*H*), 3.42 (dd, J = 13.9, 4.8 Hz, 1H, 3-H*H*), 3.11 (dd, J = 13.9, 9.6 Hz, 1H, 3-*H*H); ^{13}C NMR (101 MHz, CD$_3$OD) δ 175.5 (C), 158.4 (C), 145.2 (C), 145.0 (C), 142.4 (C), 141.5 (C), 140.8 (C), 129.6 (2 × C), 129.4 (CH), 128.6 (CH), 128.0 (2 × CH), 127.4 (q, $^1J_{C\text{-}F}$ = 167.3 Hz, C), 126.2 (C), 126.0 (CH), 123.5 (2 × C), 122.3 (2 × CH), 121.5 (CH), 120.7 (2 × CH), 116.0 (q, $^3J_{C\text{-}F}$ = 3.7 Hz, CH), 112.1 (2 × CH), 108.8 (q, $^3J_{C\text{-}F}$ = 4.4 Hz, CH), 68.0 (CH$_2$), 57.4 (CH), 48.3 (CH), 38.9 (CH$_2$); ^{19}F NMR (377 MHz, CD$_3$OD) δ −62.3 (s); MS (ESI) *m/z* 567 (M + H$^+$, 100); HRMS (ESI) *m/z*: [M + H]$^+$ Calcd for $C_{31}H_{23}F_3N_2NaO_4$ 567.1502; Found 567.1509.

(2*S*)-2-[(*tert*-Butoxycarbonyl)amino]-3-(7-cyano-9*H*-carbazol-3'-yl)propanoic acid (S2)

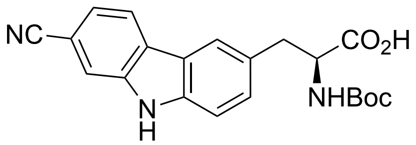

A solution of methyl (2*S*)-2-[(*tert*-butoxycarbonyl)amino]-3-(7-cyano-9*H*-carbazol-3'-yl)propanoate (**12d**) (235 mg, 0.600 mmol) and lithium hydroxide monohydrate (63.0 mg, 1.50 mmol) in methanol (10 mL) and water (4 mL) was stirred overnight at room temperature. On concentration to dryness, the residue was redissolved in water (50 mL), acidified to pH 1 with 1 M HCl and extracted with dichloromethane (2 × 30 mL). The combined organic layers were dried over MgSO$_4$ and evaporated to give (2*S*)-2-[(*tert*-butoxycarbonyl)amino]-3-(7-cyano-9*H*-carbazol-3'-yl)propanoic acid (**S2**) a colourless solid (221 mg, 97%). Mp 173−174 °C; $[\alpha]_D^{25}$ +16.4 (*c* 1.0, CHCl$_3$); IR (neat) 3328 (NH), 2978 (CH), 2360, 2221 (CN), 1688 (CO), 1635, 1491, 1248, 1156, 751 cm^{-1}; NMR spectroscopy showed a mixture of rotamers. Data is given for the major rotamer: ^{1}H NMR (400 MHz, CDCl$_3$) δ 9.19 (br s, 1H, OH), 8.92 (s, 1H, NH), 7.86–7.63 (m, 2H, 6'-H and 8'-H), 7.52 (s, 1H, 4'-H), 7.18–7.14 (m, 3H, 1'-H, 2'-H and 5'-H), 5.26 (d, J = 7.6 Hz, 1H, 2-NH), 4.67 (dt, J = 7.6, 6.8 Hz, 1H, 2-H), 3.14–3.31 (m, 2H, 3-H$_2$), 1.38 (s, 9H, 3 × CH$_3$); ^{13}C NMR (101 MHz, CDCl$_3$) δ 175.8 (C), 155.8 (C), 139.9 (CH), 138.5 (CH), 128.9 (C), 127.5 (C), 126.2 (C), 122.1 (2 × CH), 121.4 (C), 120.6 (C), 120.3 (C), 114.9 (CH), 111.4 (C), 107.4 (CH), 80.7 (C), 55.2 (CH), 38.0 (CH$_2$), 28.3 (3 × CH$_3$); MS (ESI) *m/z* 378 ([M – H]$^-$, 100); HRMS (ESI) *m/z*: [M − H]$^-$ Calcd for $C_{21}H_{20}N_3O_4$ 378.1459; Found: 378.1457.

3. Peptide Synthesis

Analysis: High resolution mass spectrometry (HRMS) was performed on either a Bruker microTOF-Q High Resolution Mass Spectrometer using ESI+ mode, or an Agilent 6200 series TOF/65000 series Q-TOF High Resolution Mass Spectrometer using ESI+ mode. Circular dichroism measurements were performed on a JASCO J-810 circular dichroism spectropolarimeter using a 0.1 cm pathlength cuvette. Far UV measurements were recorded at 5 °C and thermal melts were recorded from 5–80 °C at 216 nm.

Synthesis: Peptides were synthesised on a CEM Liberty Blue peptide synthesis instrument using the Fmoc/tBu protecting group strategy and standard DIC/OxymaPure activation, using Rink amide resin. Cleavage from the resin was performed using a cocktail of 94% TFA, 2.5% H_2O and 2.5% TIPS and 1% mercaptoethanol in sequences containing a cysteine, and using 95% TFA, 2.5% H_2O and 2.5% TIPS in those without cysteine. Purification was performed on a Dionex P680 semi-preparative HPLC system using either a Phenomenex, Gemini C18, 5 µm, 250 × 21.2 mm column or a Phenomenex Luna C18, 5 µm, 150 × 10 mm column at a flow rate of either 8 mL/min or 3 mL/min. Gradients were run using a binary solvent system consisting of solution A (H_2O + 0.1% TFA) and B (MeCN + 0.1% TFA). Reverse-phase HPLC analysis was performed on a Shimadzu system with a UV-Vis detector monitoring at 214 nm and 280 nm. The column used was a Phenomenex, Aeris, 5 µm, peptide XB-C18, 150 × 4.6 mm at a flow rate of 1 mL/min. Gradients were run using a binary solvent system consisting of solution A (5% MeCN in H_2O + 0.1% TFA) and B (5% H_2O in MeCN + 0.1% TFA). Peptide content was analysed on a Thermo Scientific NanoDrop One UV-Vis spectrophotometer.

TrpZip Peptide 14 Containing (2*S*)-2-Amino-3-(7-trifluoromethyl-9*H*-carbazol-3'-yl)propanoic acid hydrochloride (13e)

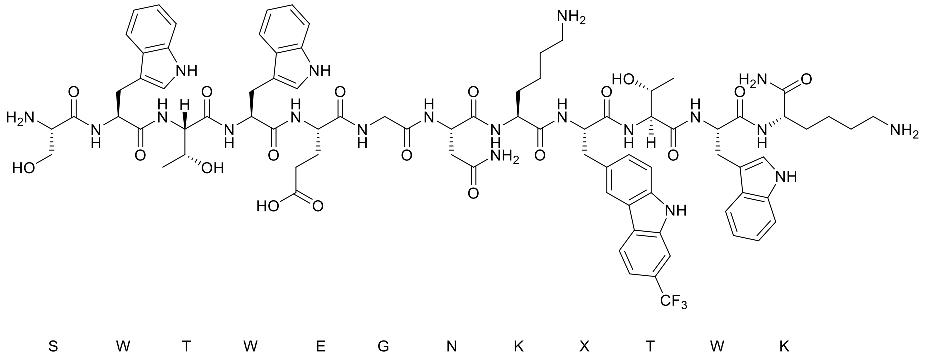

The peptide was synthesised through standard microwave-assisted SPPS, using Rink amide AM resin (0.58 mmol/g loading) on a 0.1 mmol scale. The automated synthesis was carried out in two parts, the first stopping after 3 couplings. The carbazole amino acid residue was then incorporated using a manual coupling. The manual coupling was carried out using methyl (2*S*)-2-[(9*H*-fluoren-9-ylmethoxycarbonyl)amino]-3-(7-(trifluoromethyl)-9H-carbazol-3-yl)propanoic acid (**S1**) (1.5 eq.,

0.152 mmol), (benzotriazol-1-yloxytripyrrolidinophosphonium hexafluorophosphate) (1.5 eq., 0.152 mmol) and diisopropylethylamine (4 eq., 0.4 mmol). The reaction mixture and resin were left to stir at room temperature for 1 h. The peptide synthesis was then completed using standard microwave-assisted SPPS. The crude material was purified using a gradient of 20 to 70% of solution B.

HRMS (ESI) *m/z*: $[M + H]^+$ Calcd for $C_{83}H_{103}F_3N_{20}O_{18}$ 1724.7695; Found 1725.7767.

HPLC trace for TrpZip peptide **14**, indicating retention time and purity:

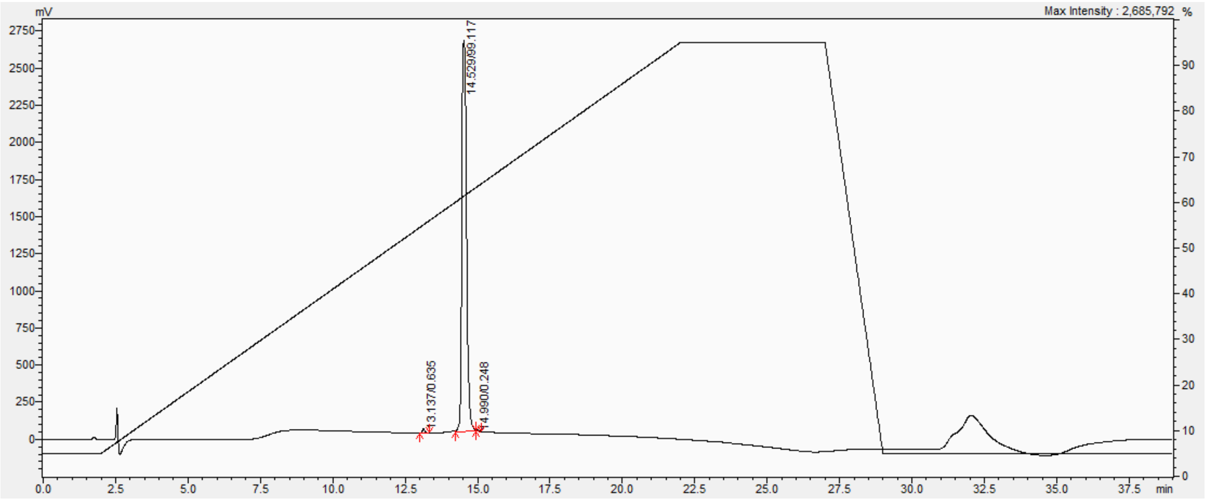

Retention time 14.5 min, purity 99% (20-minute gradient).

L30K WW Domain Peptide 15

H-FEIPDDVPLPAGWEMAKTSSGQRYFKNHIDQTTTWQDPRK-OH

WW domain peptide **15** was synthesised on a 0.1 mmol scale using microwave-assisted SPPS using H-Lys(Boc)-HMPB-ChemMatrix resin (0.4–0.65 mmol/g loading). The final 18 residues were double coupled. The peptide was cleaved from the resin using 2.5% TIPS, 2.5% water, 5% BME and 90% TFA for 4 h. Following purification with RP-HPLC the peptide was obtained as a white solid.

MS (ESI) *m/z*: $[M - 3H]^{3-}$ Calcd for $C_{210}H_{310}N_{57}O_{64}S$ 1562.08; Found 1562.08.

HPLC trace for peptide **15**, indicating retention time and purity:

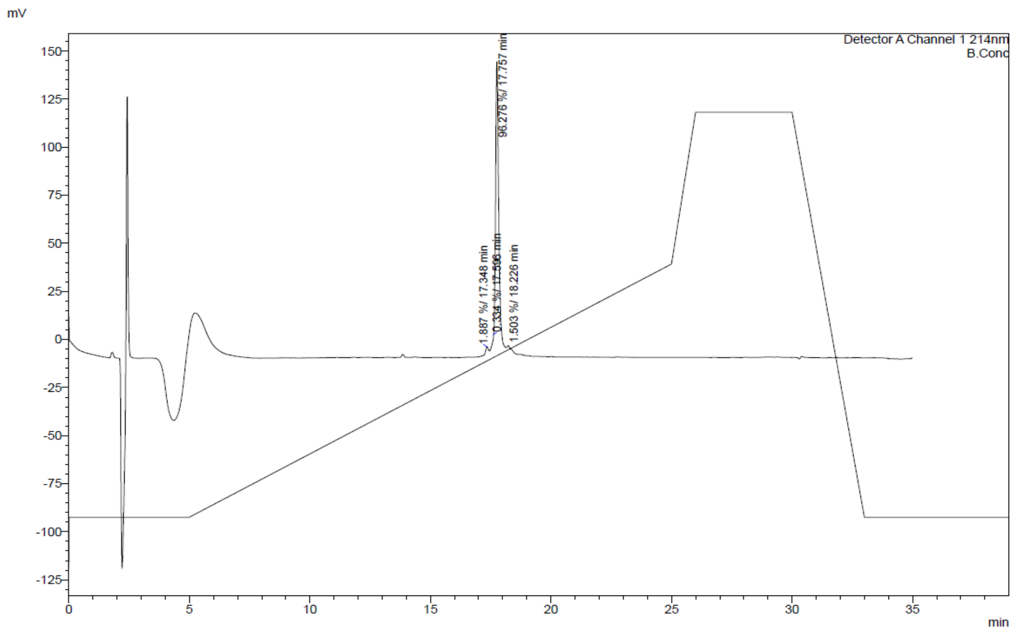

Retention time 17.8 min, purity 99% (20-minute gradient).

Fluorescent Peptide ligand 16 Containing (2*S*)-2-Amino-3-(7-cyano-9*H*-carbazol-3'-yl)propanoic acid hydrochloride (13d) for WW Protein Assay

H_2N-**13d**-Gly-Ser-Gly-Thr-Pro-Pro-Pro-Pro-Tyr-Thr-Val-Gly-OH

The peptide was synthesised through standard microwave-assisted SPPS, using Fmoc-Gly-Wang resin (0.31 mmol/g loading) on a 0.1 mmol scale. Following preparation of the undecapeptide, the carbazole amino acid residue was then incorporated using a manual coupling. The manual coupling was carried out using (2*S*)-2-((*tert*-butoxycarbonyl)amino)-3-(7-cyano-9*H*-carbazol-3-yl)propanoic acid (**S2**) (1.5 eq) was coupled using DIPEA (3 eq), PyBOP (2 eq) in DMF (1.5 mL) for 2 h. For deprotection and cleavage, 95% TFA, 2.5% water and 2.5% triisopropylsilane (2 mL total volume) were added to the resin, which was mixed gently for 2 h. The reaction mixture was evaporated under a flow of nitrogen gas followed by precipitation of the peptide in cold diethyl ether (50 mL). The precipitate was dissolved in 1:1 H_2O/MeCN and lyophilised to give an off-white powder. The crude material was purified using a gradient of 20 to 70% solution B.

MS (ESI) *m/z*: $[M + H]^+$ Calcd for $C_{67}H_{88}N_{15}O_{18}$ 1390.6; Found 1390.6.

HPLC trace for peptide **16**, indicating retention time and purity:

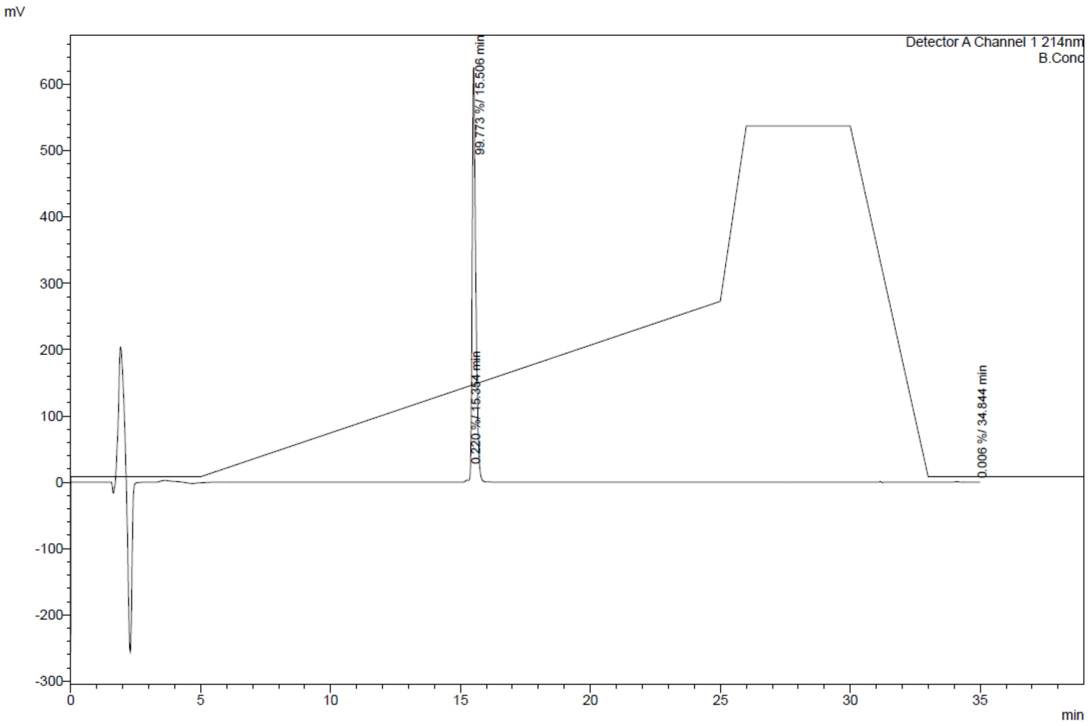

Retention time 15.5 min, purity >99% (20-minute gradient).

4. Fluorescence titration

Fluorescence titration was carried out using 10 μM of ligand peptide **16**. A 0.5 mM solution of WW domain **15** was titrated into the ligand peptide solution. Both solutions were made up using 20 mM of 3-morpholinopropanesulfonic acid (MOPS) buffer solution (pH 7.0). An excitation wavelength of 340 nm was used and the change in fluorescence upon addition of the WW domain measured at 405 nm. The fluorescence decreases as the WW domain is titrated into the ligand peptide solution. The K_d value for the curve is 59 μM.

Emission Spectra of peptide ligand **16** on addition of WW domain protein **15** (from 0 to 400 □mol l^{-1}):

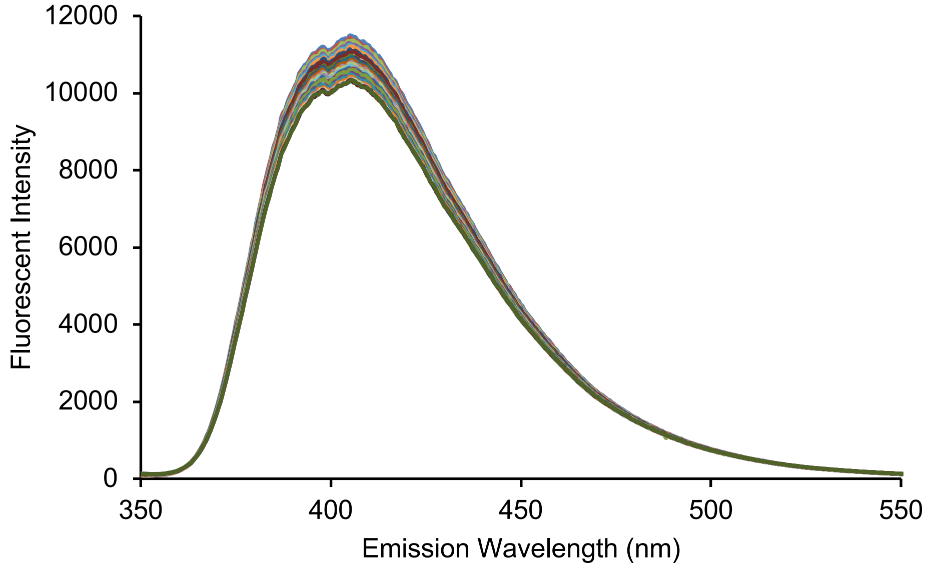

5. Photophysical Data for α-Amino Acids 13a–e

Absorption and emission data were recorded using the following instruments:

1. UV-Vis spectra were recorded on a Perkin Elmer Lamda 25 instrument. Fluorescence spectra were recorded on a Shimadzu RF-5301PC spectrofluorophotometer. Emission data were measured using excitation and emission bandpass filters of 3 nm.

2. Both UV-Vis spectra and fluorescence spectra were recorded on a Horiba Duetta Fluorescence and Absorbance spectrometer. Absorbance spectra were recorded with an integration time of 0.05 s, and a band pass of 5 nm. Fluorescence spectra were recorded with and excitation and emission band pass of 5 nm, an integration time of 2 s, and with detector accumulations set to 1.

Quantum yields were determined using a comparative method against two standards. Anthracene (Φ = 0.27, in ethanol) and L-tryptophan (Φ = 0.14 in water) were used as standard references.[9] The integrated fluorescence intensity of each compound was determined from the emission spectra given. Measurements were performed at five different concentrations. Concentrations were chosen to ensure the absorption value was below 0.1 to avoid re-absorption effects. Integrated fluorescence intensity was plotted as a function of the measured absorbance and a linear fit was calculated. The resultant gradient was then used to calculate the quantum yield, using the equation below:

$$\phi_x = \phi_{ST}\left(\frac{Grad_{ST}}{Grad_x}\right)\left(\frac{\eta_x^2}{\eta_{ST}^2}\right)$$

Subscript *ST* signifies the quantities associated with the quantum yield standard. Subscript X signifies the quantities associated with the novel compound. $Grad_X$ is the determined gradient associated with the novel compound. $Grad_{ST}$ is the determined gradient associated with quantum yield standard. η is the refractive index of the solvent used in the fluorescence measurements. η = 1.333 for water, 1.361 for ethanol and 1.331 for methanol.

Absorption and Emission Spectra for 13a.

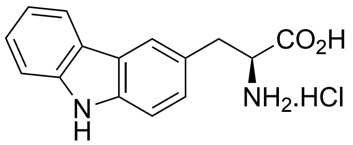

In methanol:

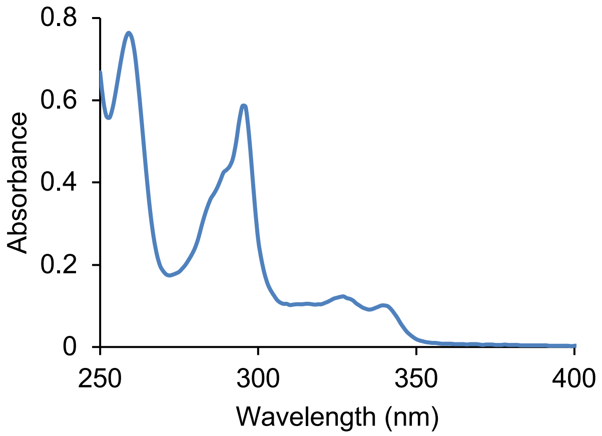

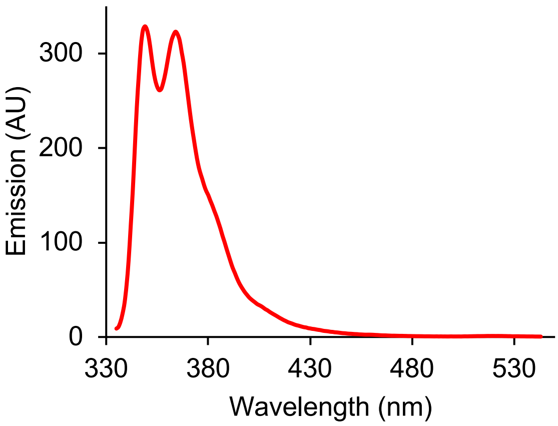

In water:

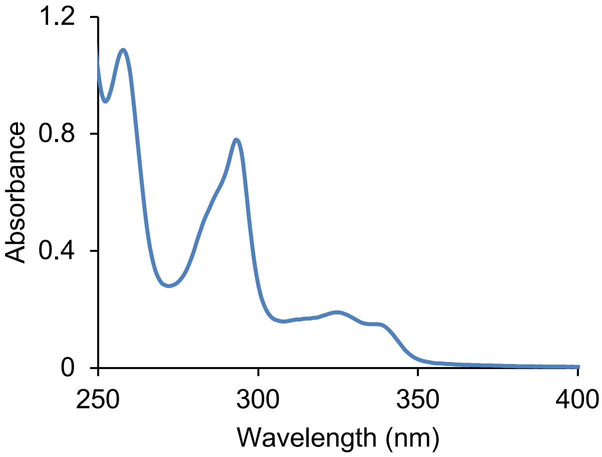

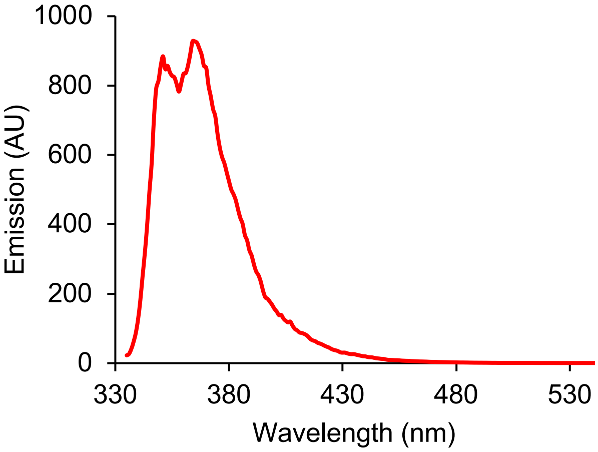

Absorption and Emission Spectra for 13b.

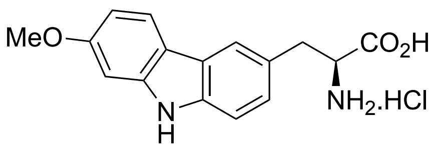

In methanol:

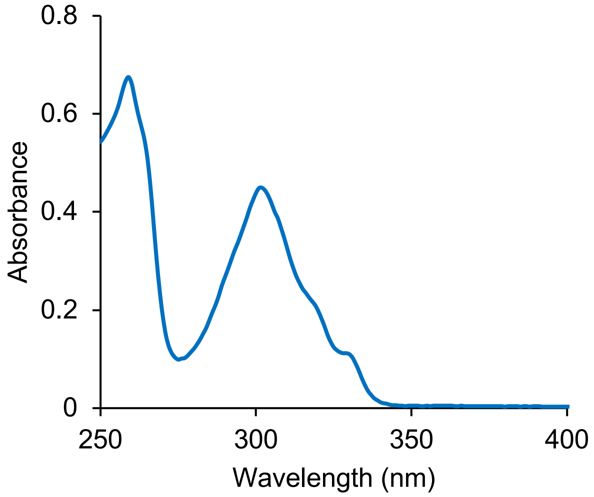

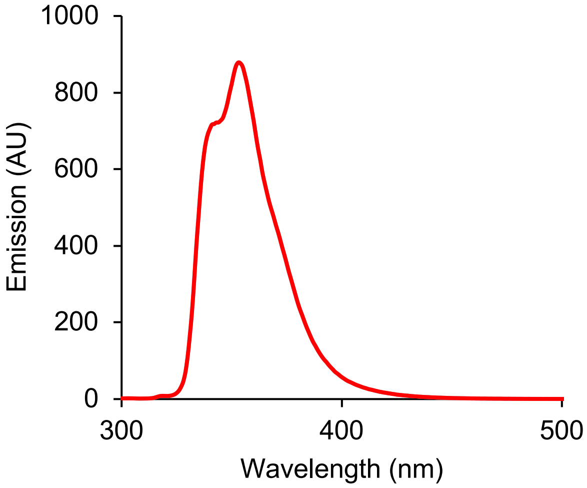

In water:

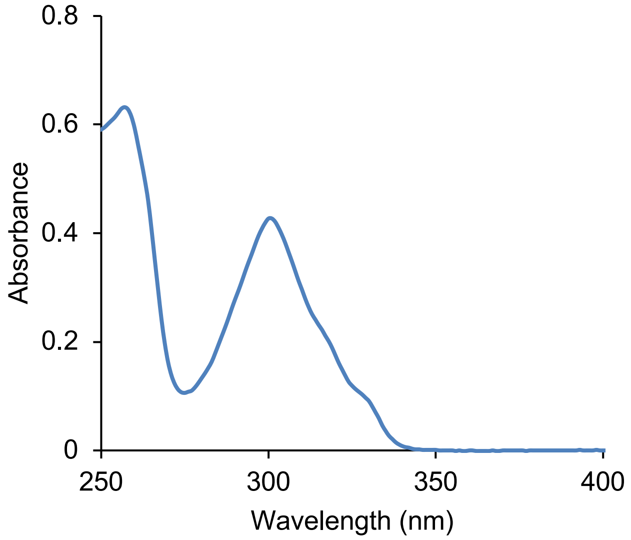

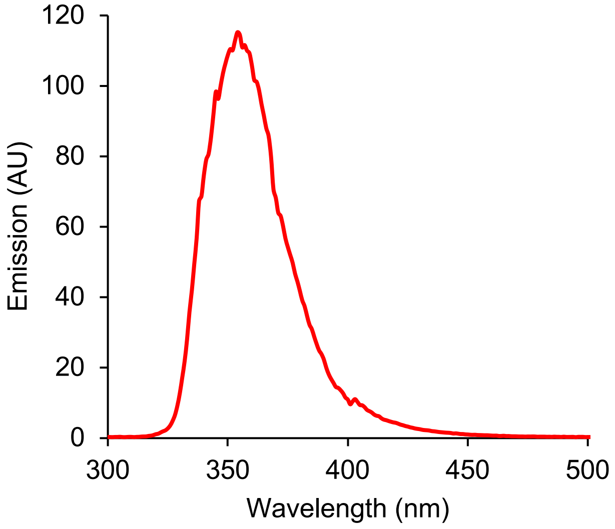

Absorption and Emission Spectra for 13c.

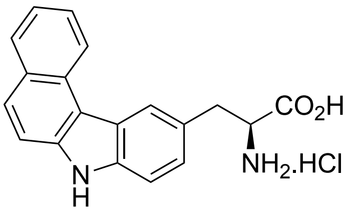

In methanol:

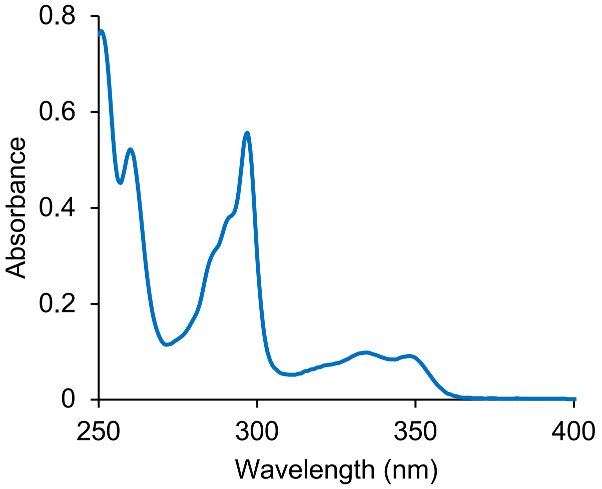

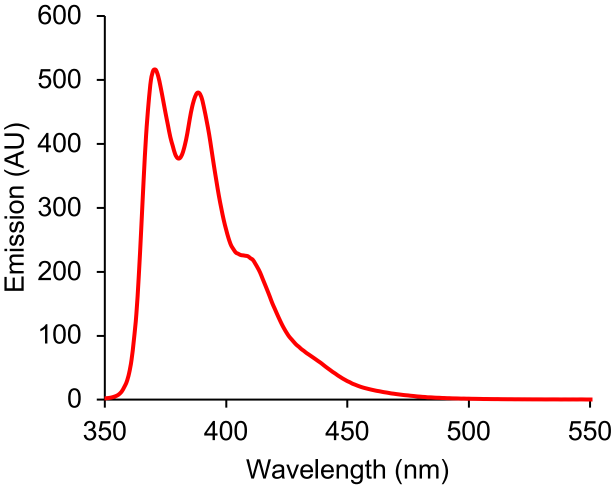

In water:

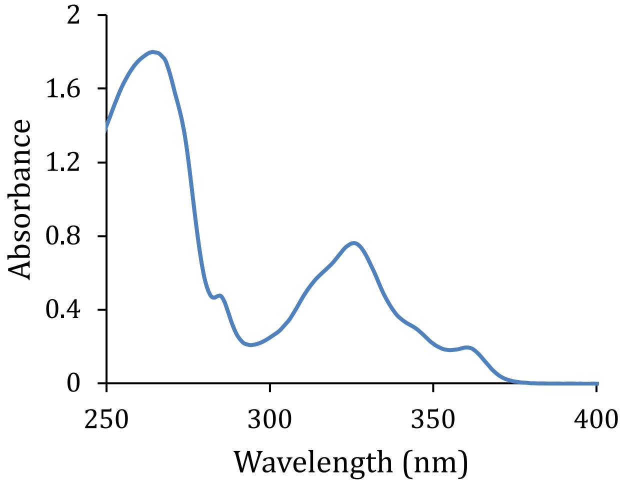

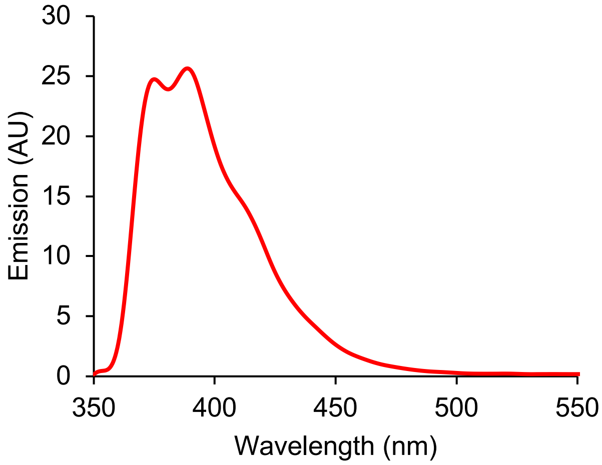

Absorption and Emission Spectra for 13d.

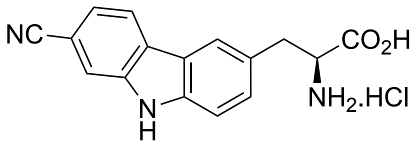

In methanol:

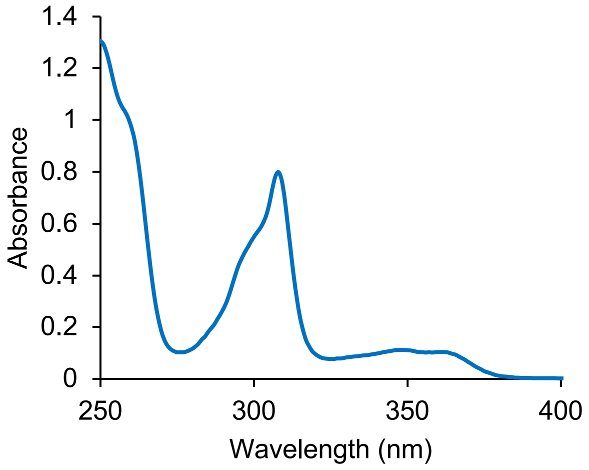

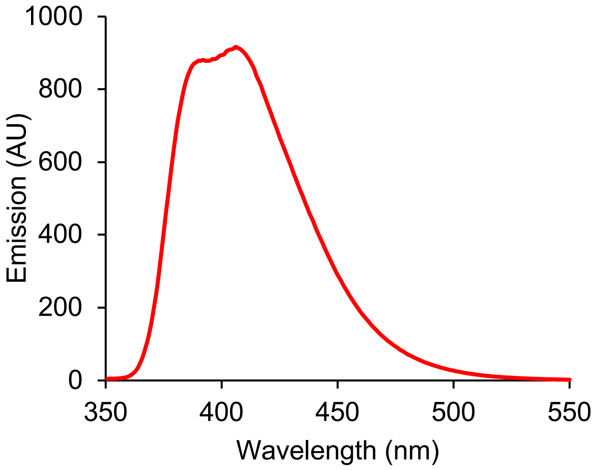

In water:

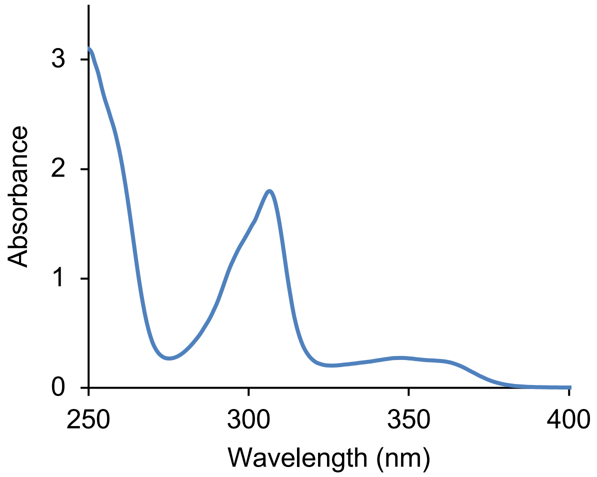

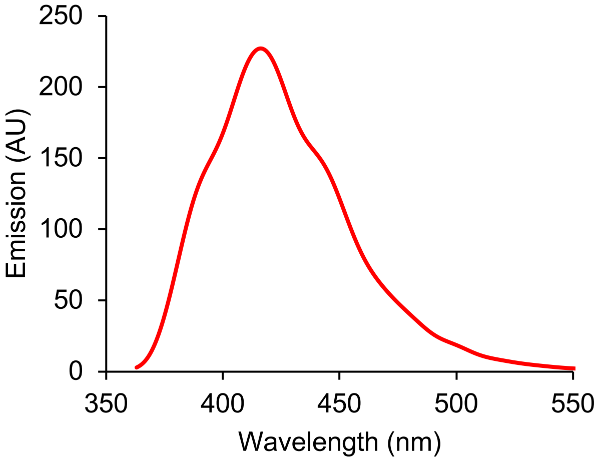

Absorption and Emission Spectra for 13e.

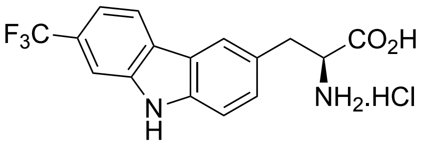

In methanol:

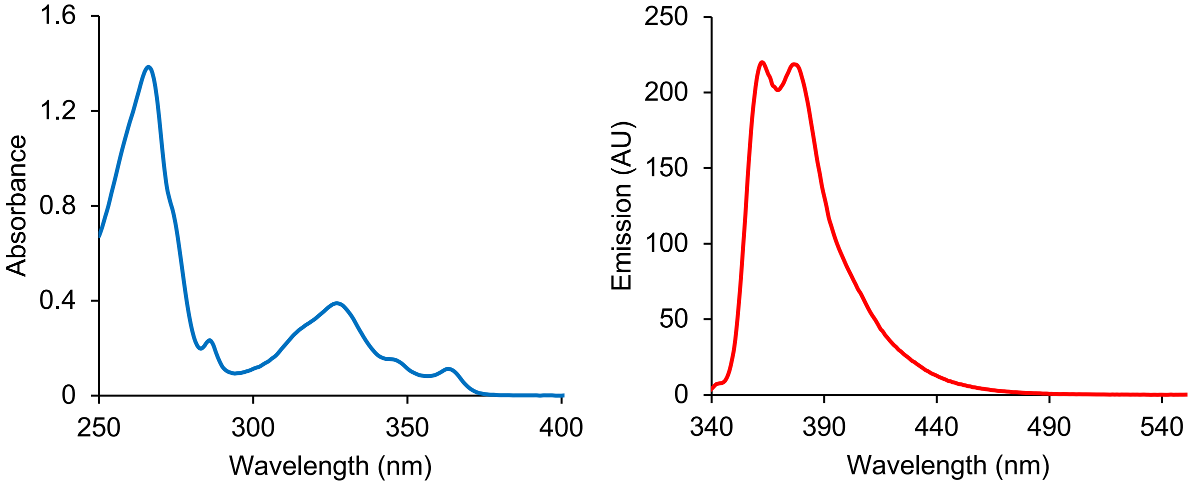

In water:

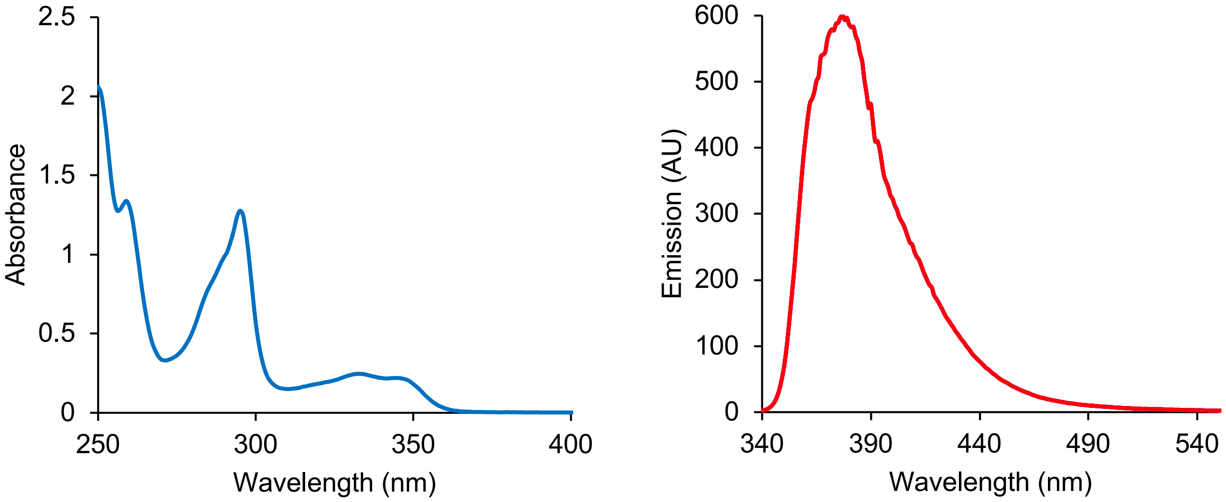

Solvatochromic Study

Absorption and Emission Spectra for 13c.

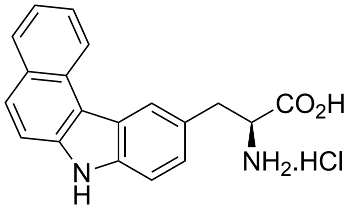

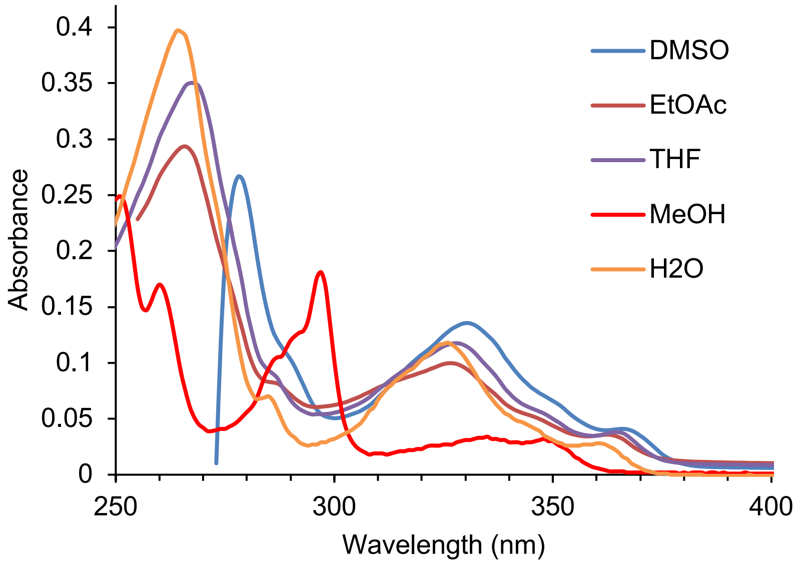

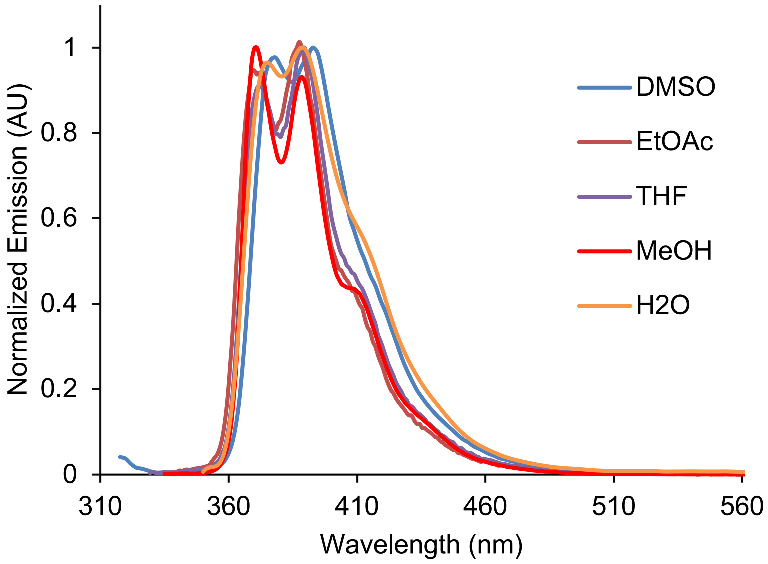

Absorption and Emission Spectra for 13d.

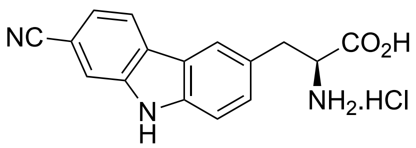

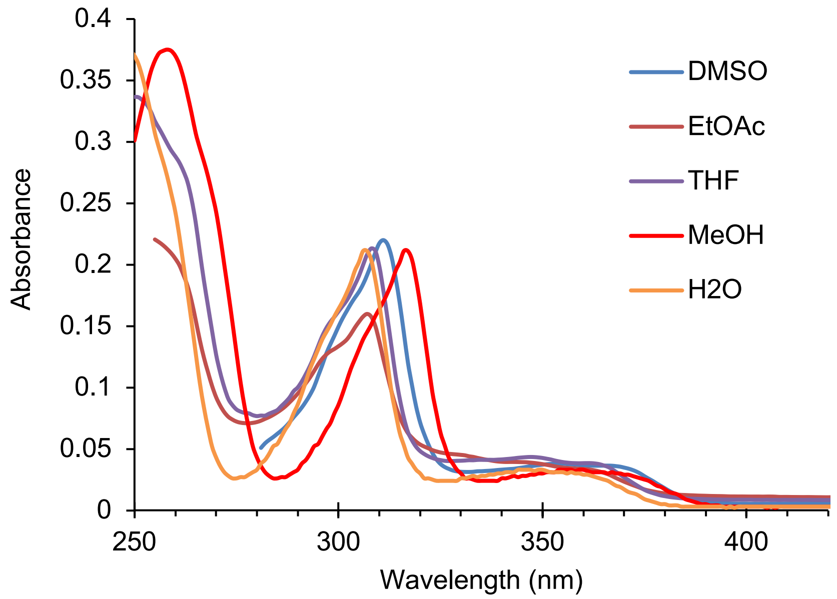

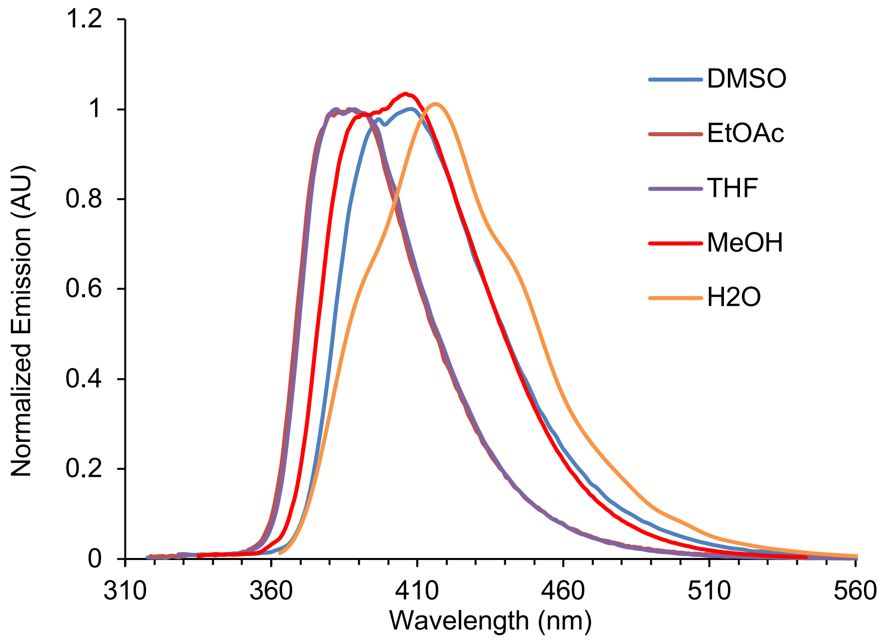

Absorption and Emission Spectra for 13e.

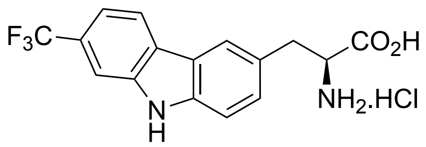

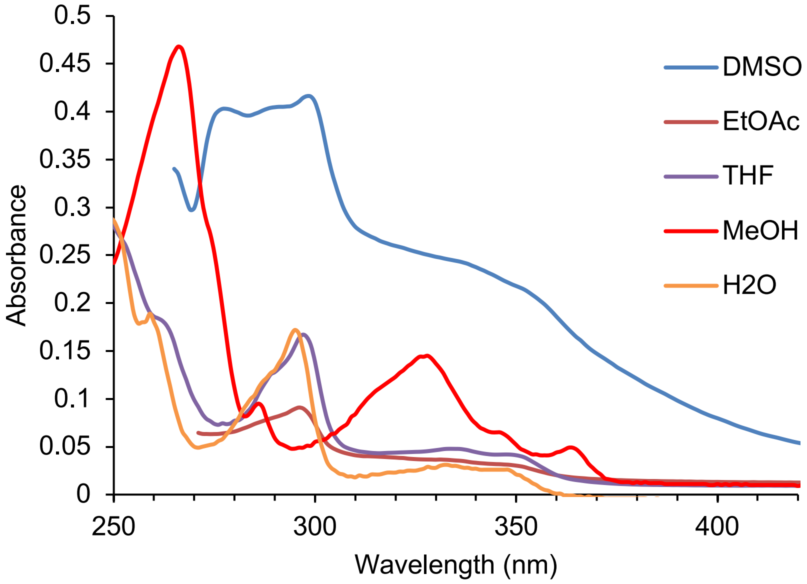

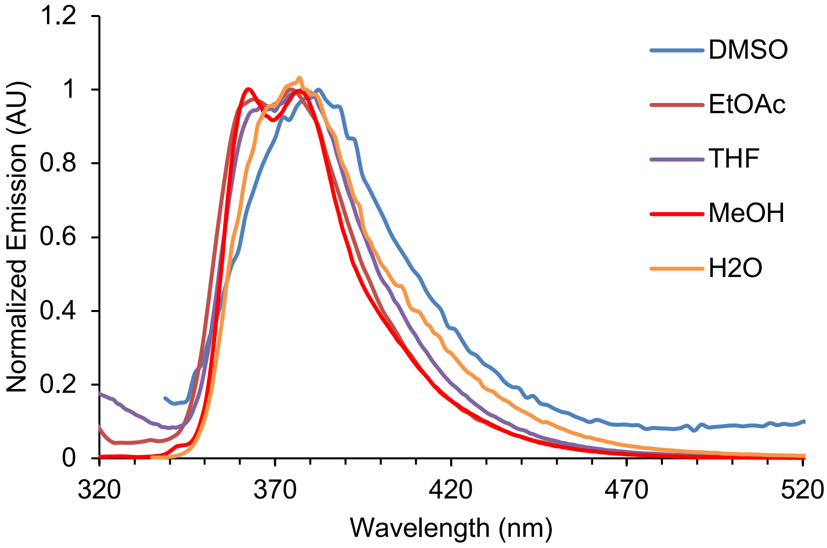

pH Study

Absorption and Emission Spectra for 13c.

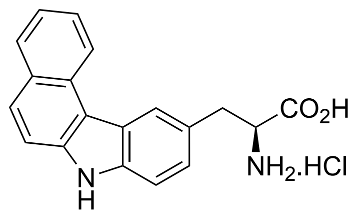

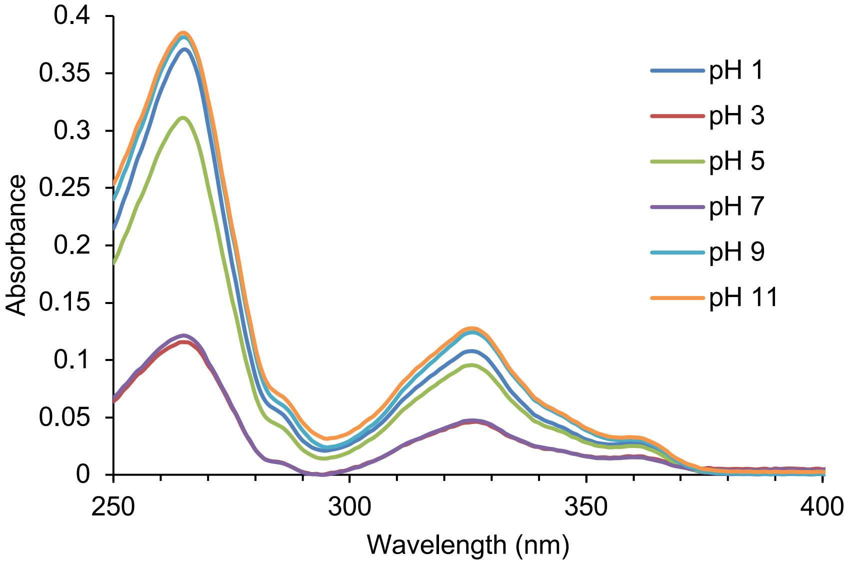

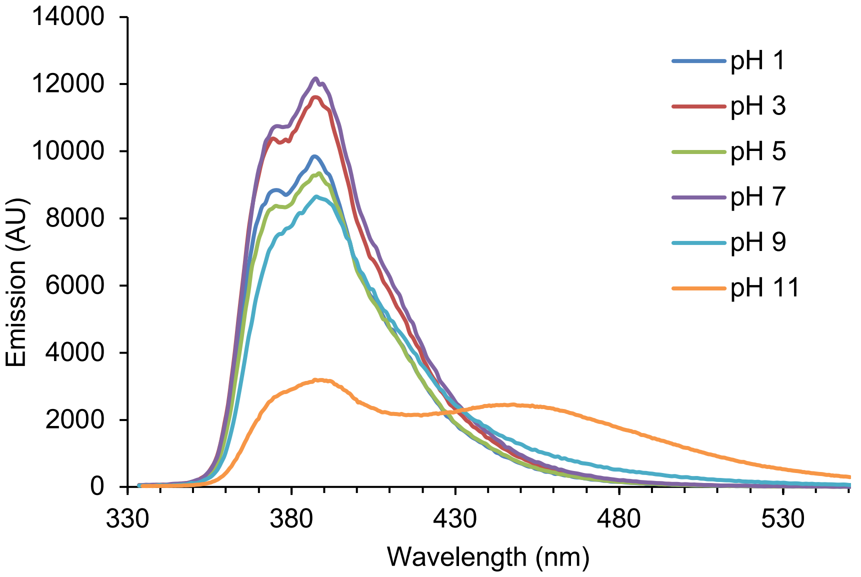

Absorption and Emission Spectra for 13d.

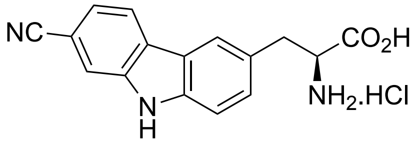

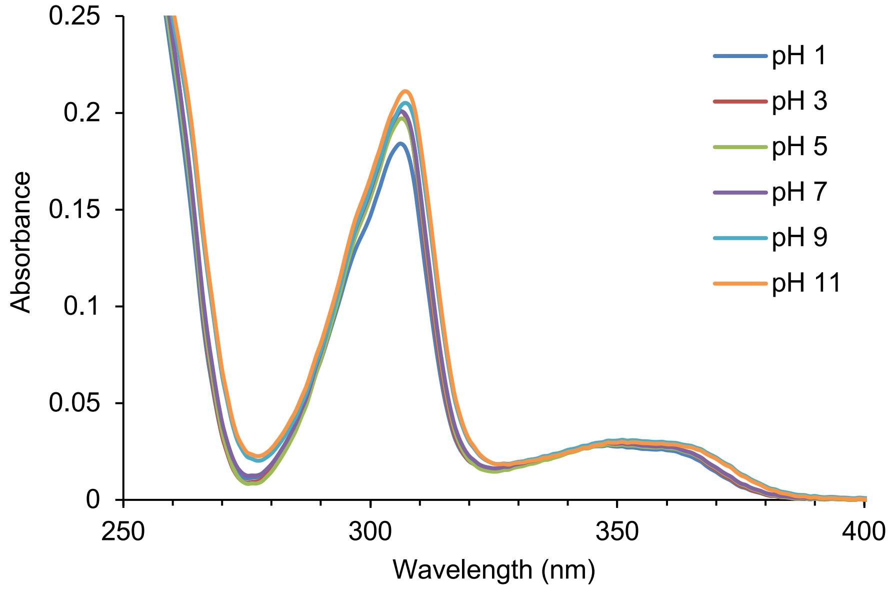

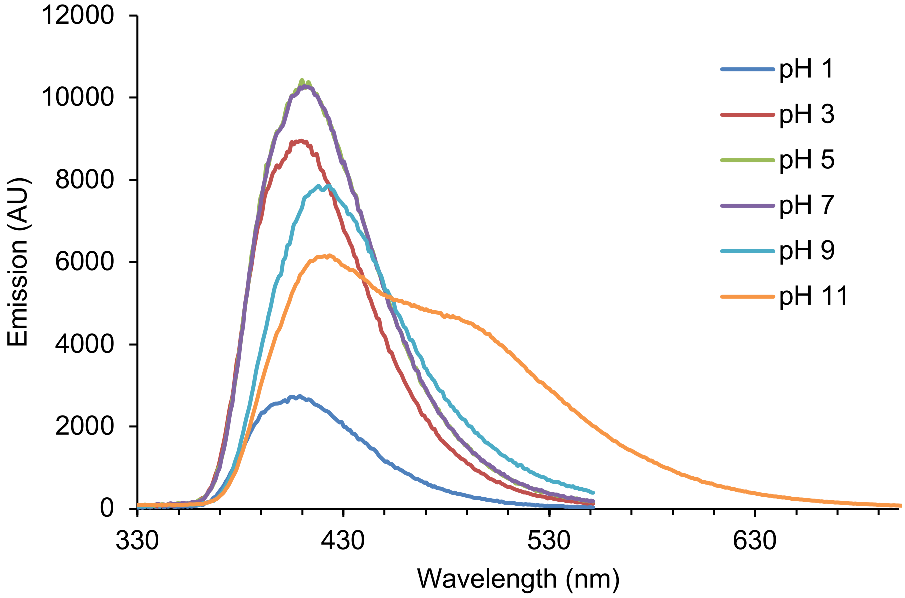

Absorption and Emission Spectra for 13e.

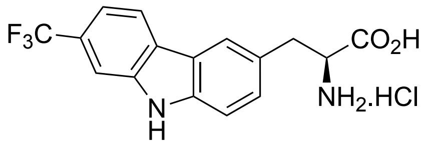

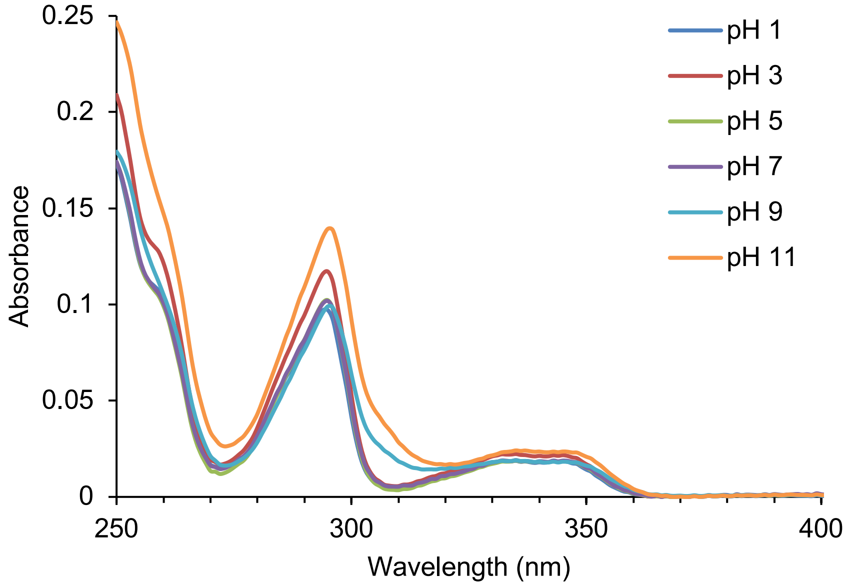

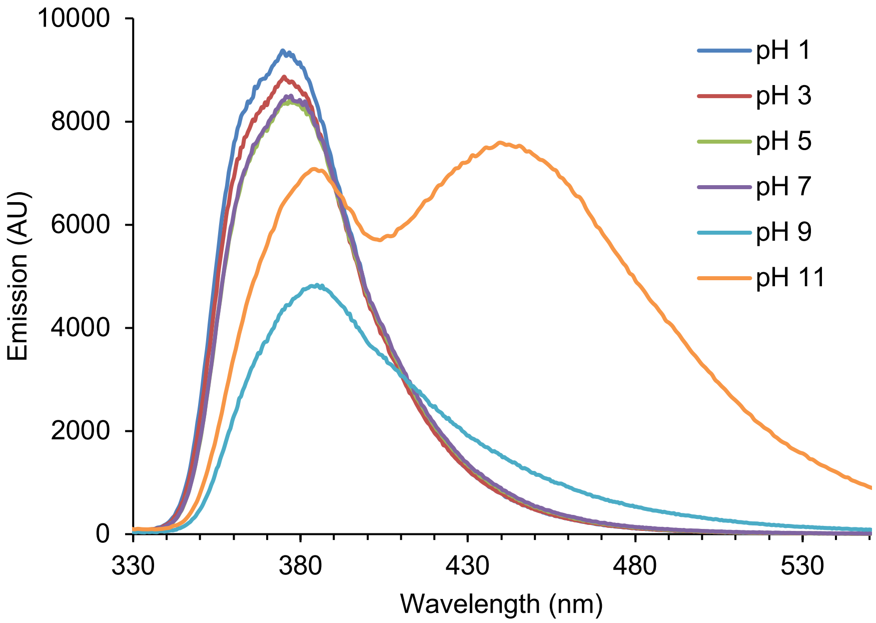

6. References

1. B. Song, T. Knauber and L. Gooβen, *Angew. Chem. Int. Ed.*, 2013, **52**, 2954−2958.

2. L. Caron, L.-C. Campeau and K. Fagnou, *Org. Lett.*, 2008, **10**, 4533−4536.

3. Q. Yan, E. Gin, M. Wasinska-Kalwa, M. G. Banwell and P. D. Carr, *J. Org. Chem.*, 2017, **82**, 4148−4159.

4. C. Suzuki, K. Hirano, T. Satoh and M. Miura, *Org. Lett.*, 2015, **17**, 1597−1600.

5. M. Pudlo, D. Csányi, F. Moreau, G. Hajós, Z. Riedl and J. Sapi, *Tetrahedron*, 2007, **63**, 10320−10329.

6. S. Maiti, T. K. Achar and P. Mal, *Org. Lett.*, 2017, **19**, 2006−2009.

7. D. Shetty, J. M. Jeong, C. H. Ju, Y. J. Kim, J.-Y. Lee, Y.-S. Lee, D. S. Lee, J.-K. Chung and M. C. Lee, *Bioorg. Med. Chem.*, 2010, **18**, 7338−7347.

8. E. Stempel, R. F.-X. Kaml, N. Budisa and M. Kalesse, *Bioorg. Med. Chem.*, 2018, **26**, 5259−5269.

9. A. T. R. Williams, S. A. Winfield and J. N. Miller, *Analyst*, 1983, **108**, 1067–1071.

7. ^{1}H and $^{13}C\{^{1}H\}$ NMR Spectra for all Compounds

^{1}H NMR (400 MHz, $CDCl_3$)

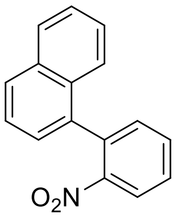

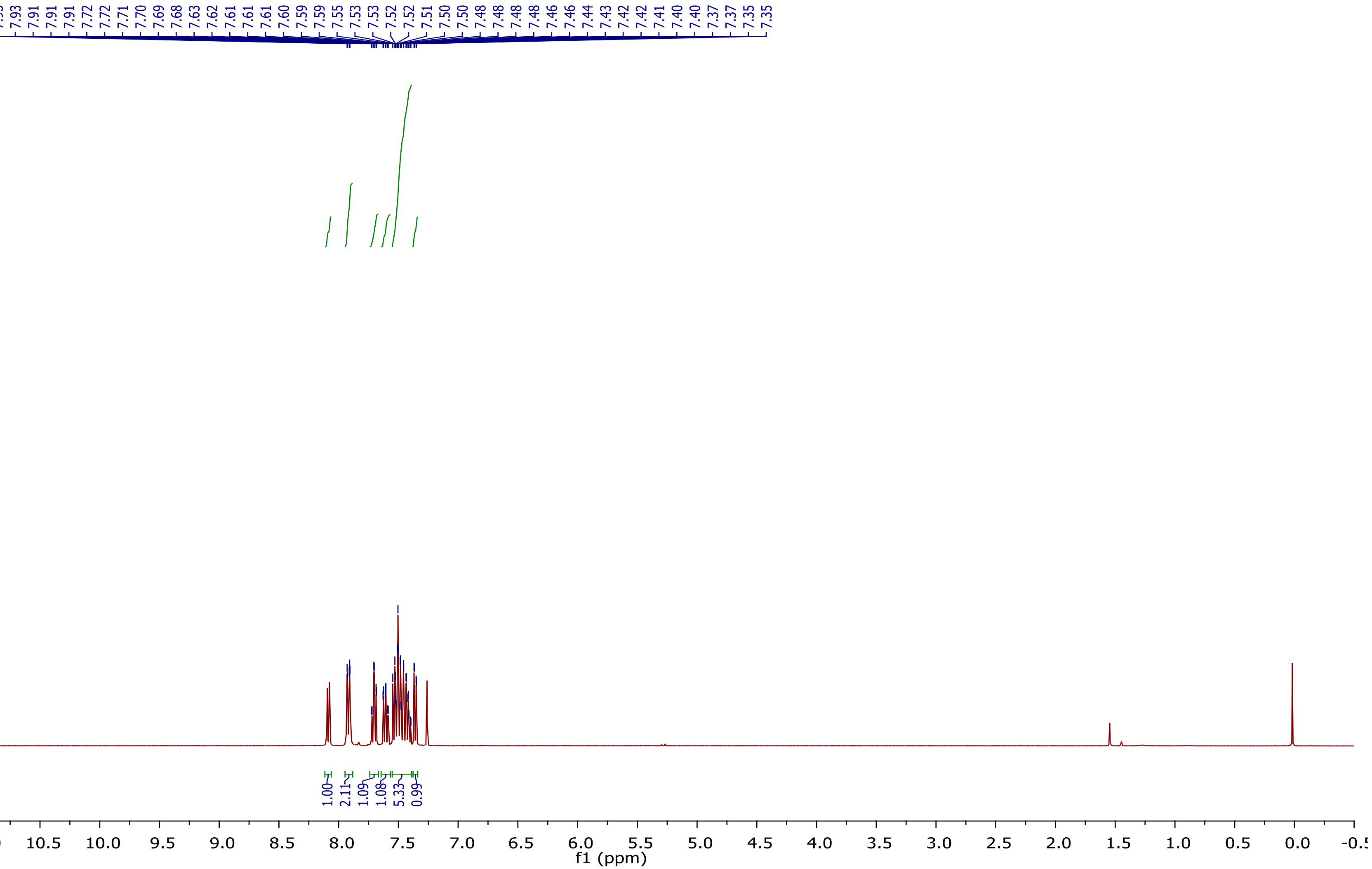

$^{13}C\{^{1}H\}$ NMR (101 MHz, $CDCl_3$)

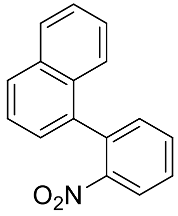

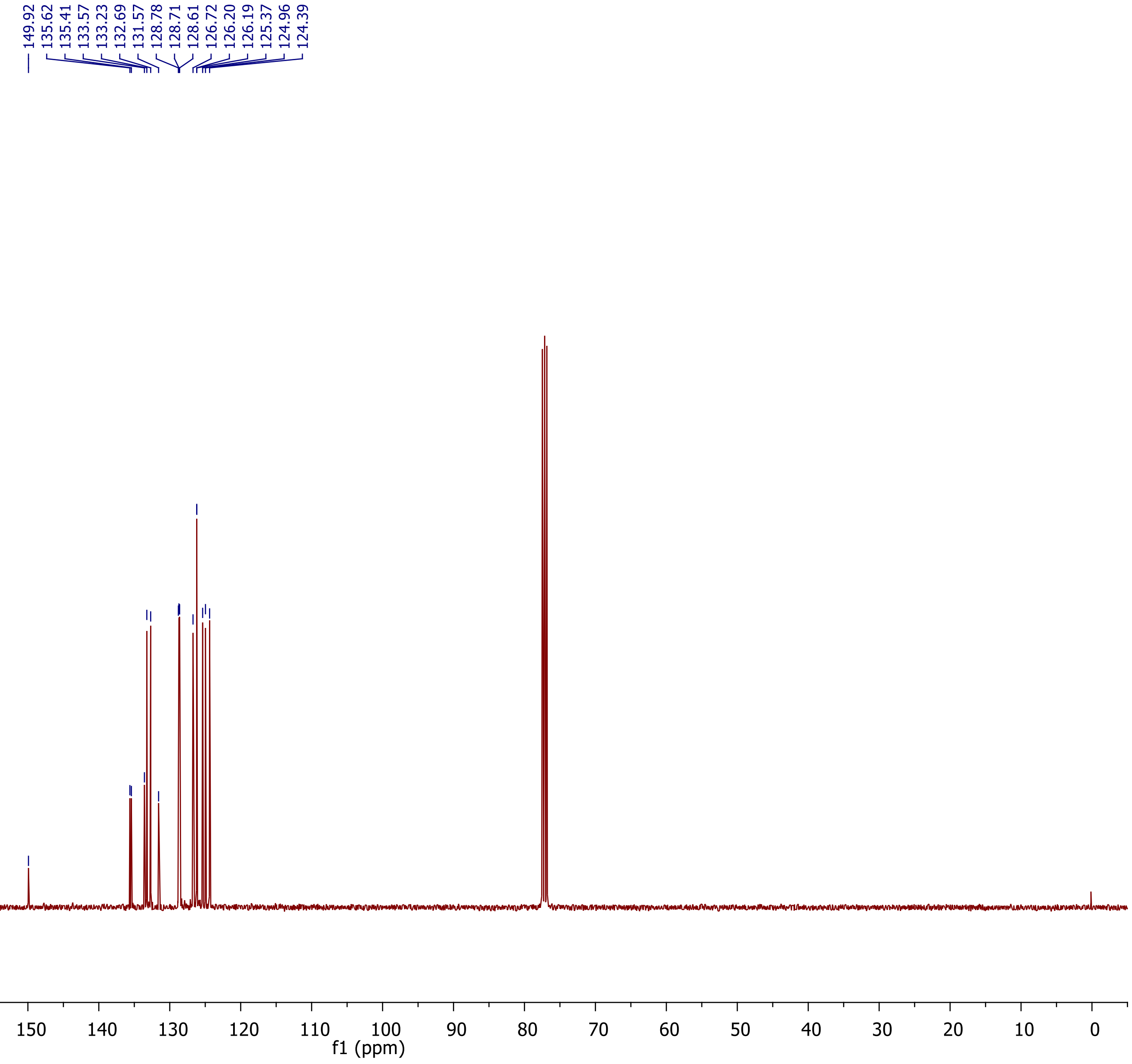

^{1}H NMR (400 MHz, $CDCl_3$)

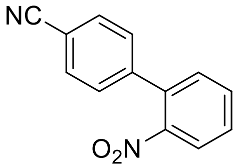

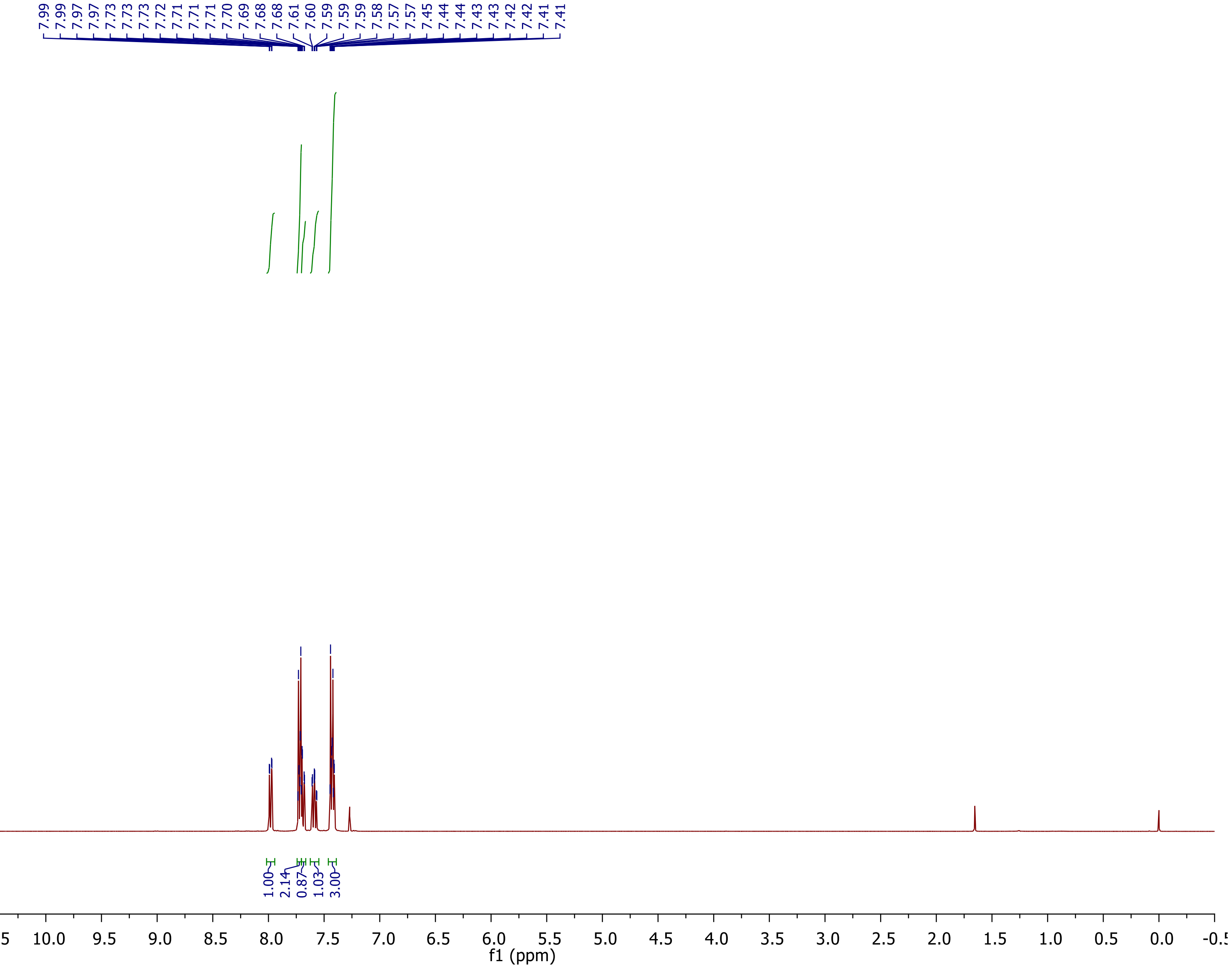

$^{13}C\{^1H\}$ NMR (101 MHz, $CDCl_3$)

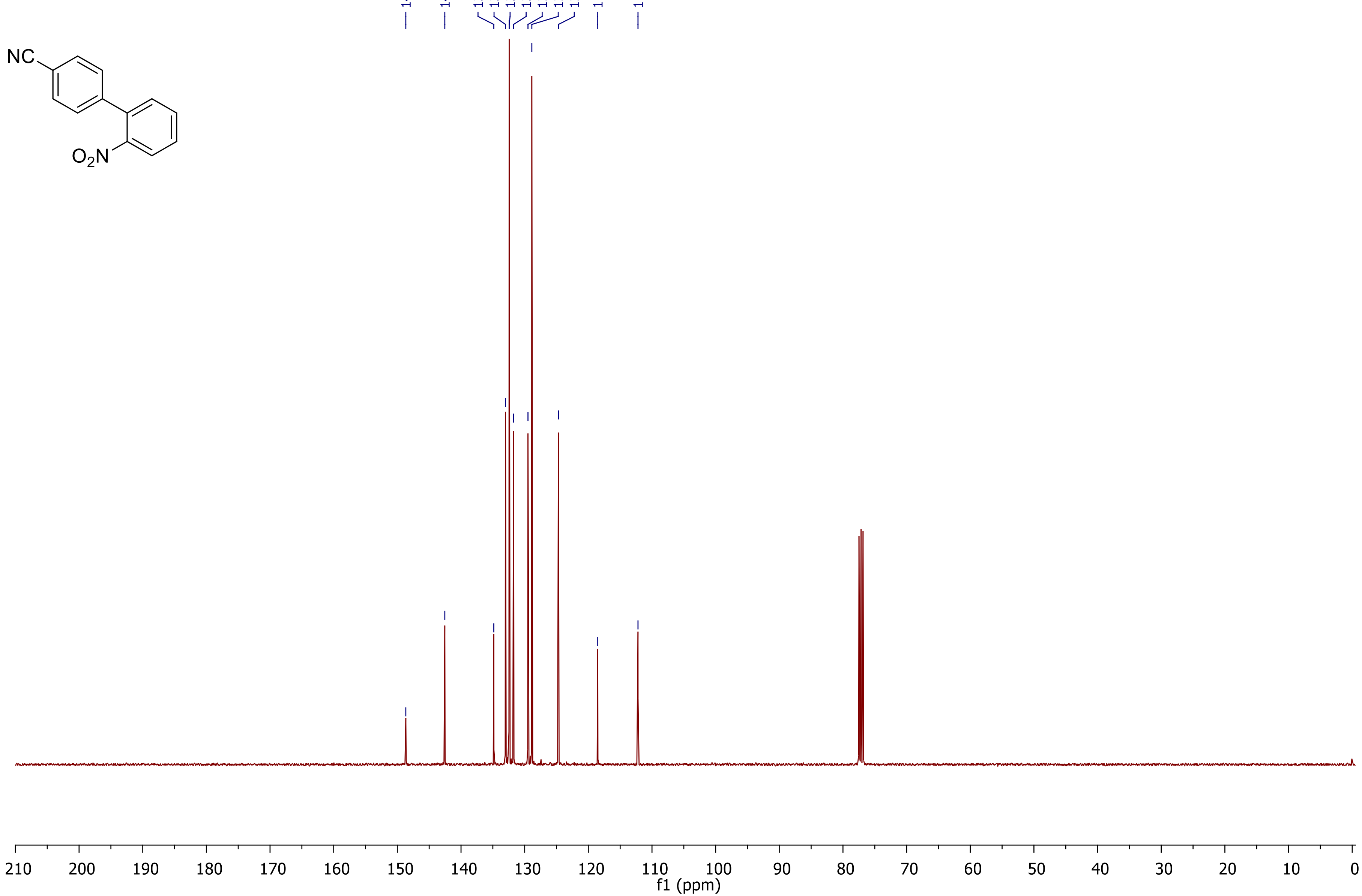

^{1}H NMR (400 MHz, $CDCl_3$)

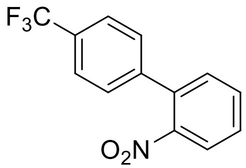

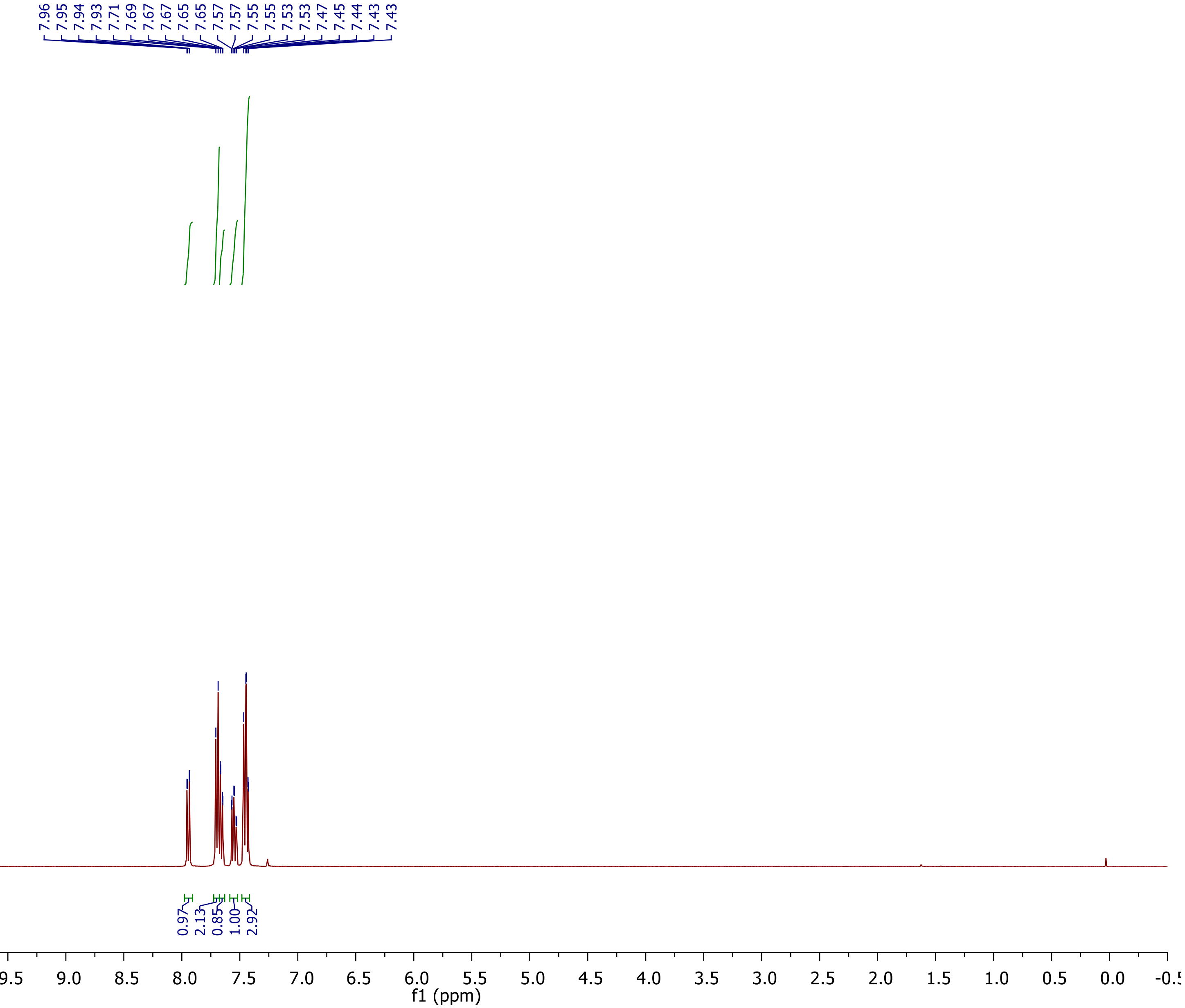

$^{13}C\{^{1}H\}$ NMR (101 MHz, $CDCl_3$)

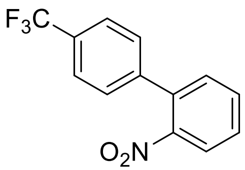

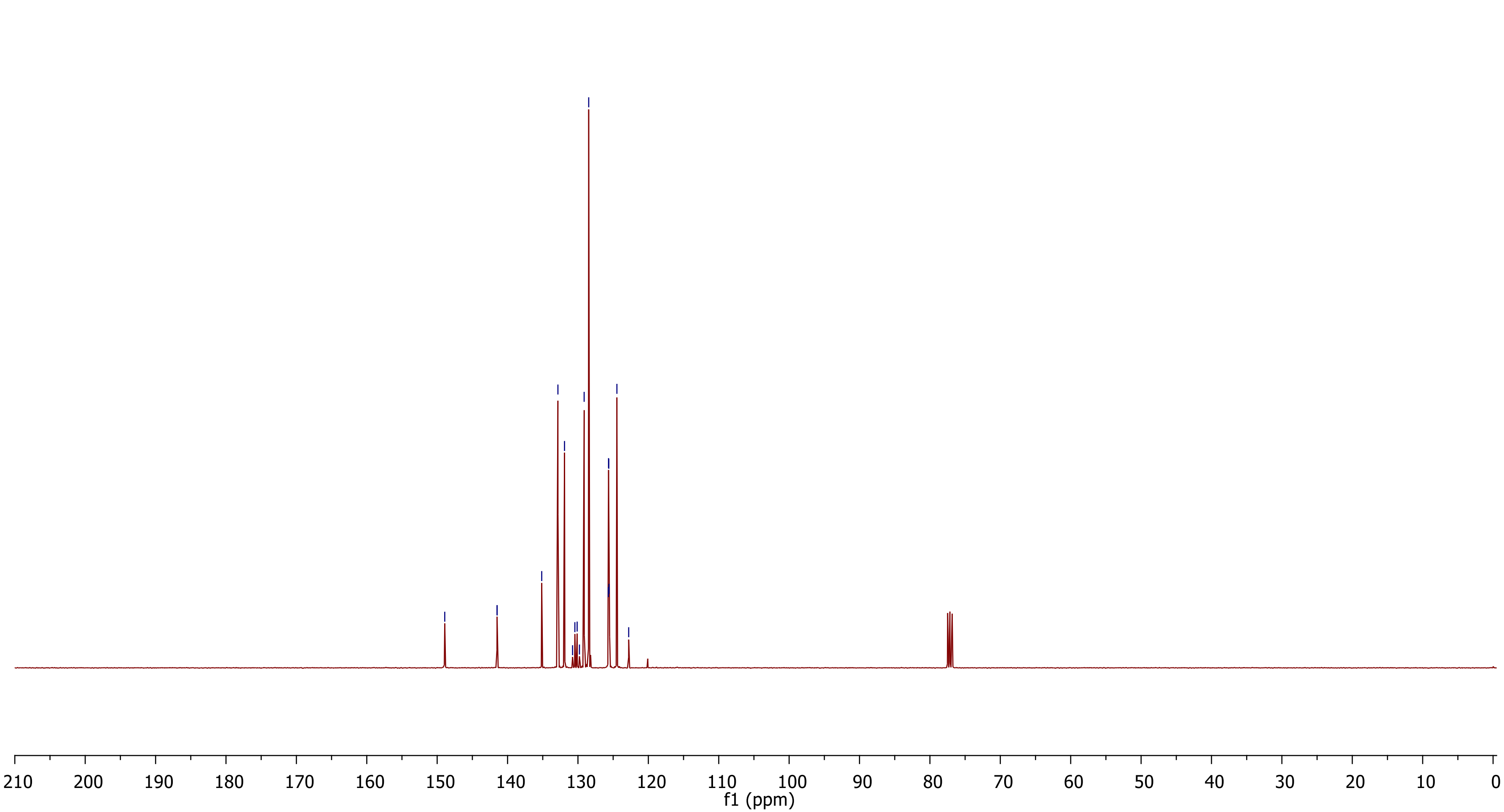

^{1}H NMR (400 MHz, $CDCl_3$)

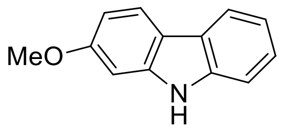

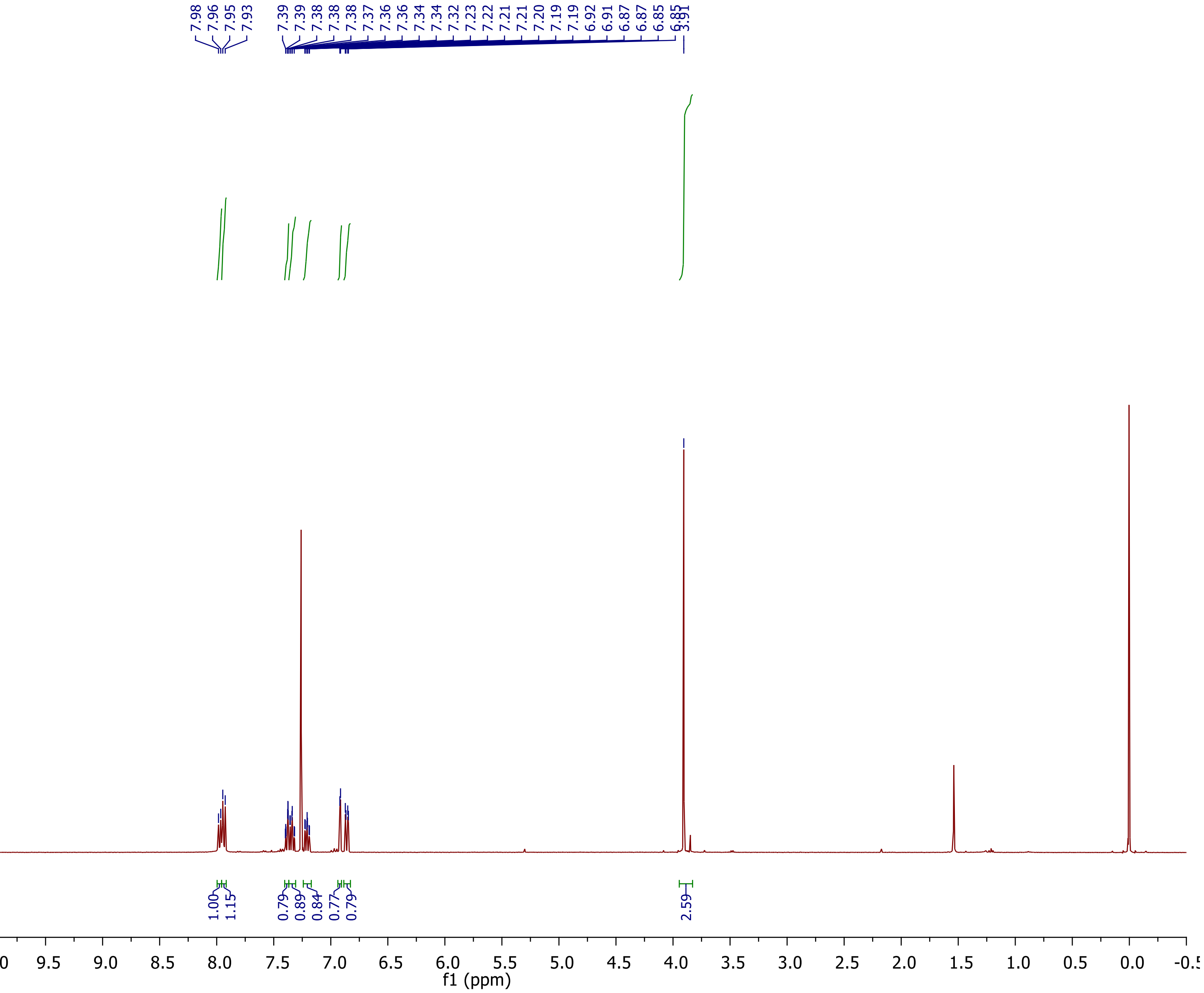

$^{13}C\{^{1}H\}$ NMR (101 MHz, $CDCl_3$)

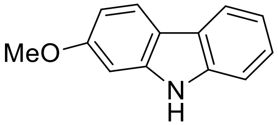

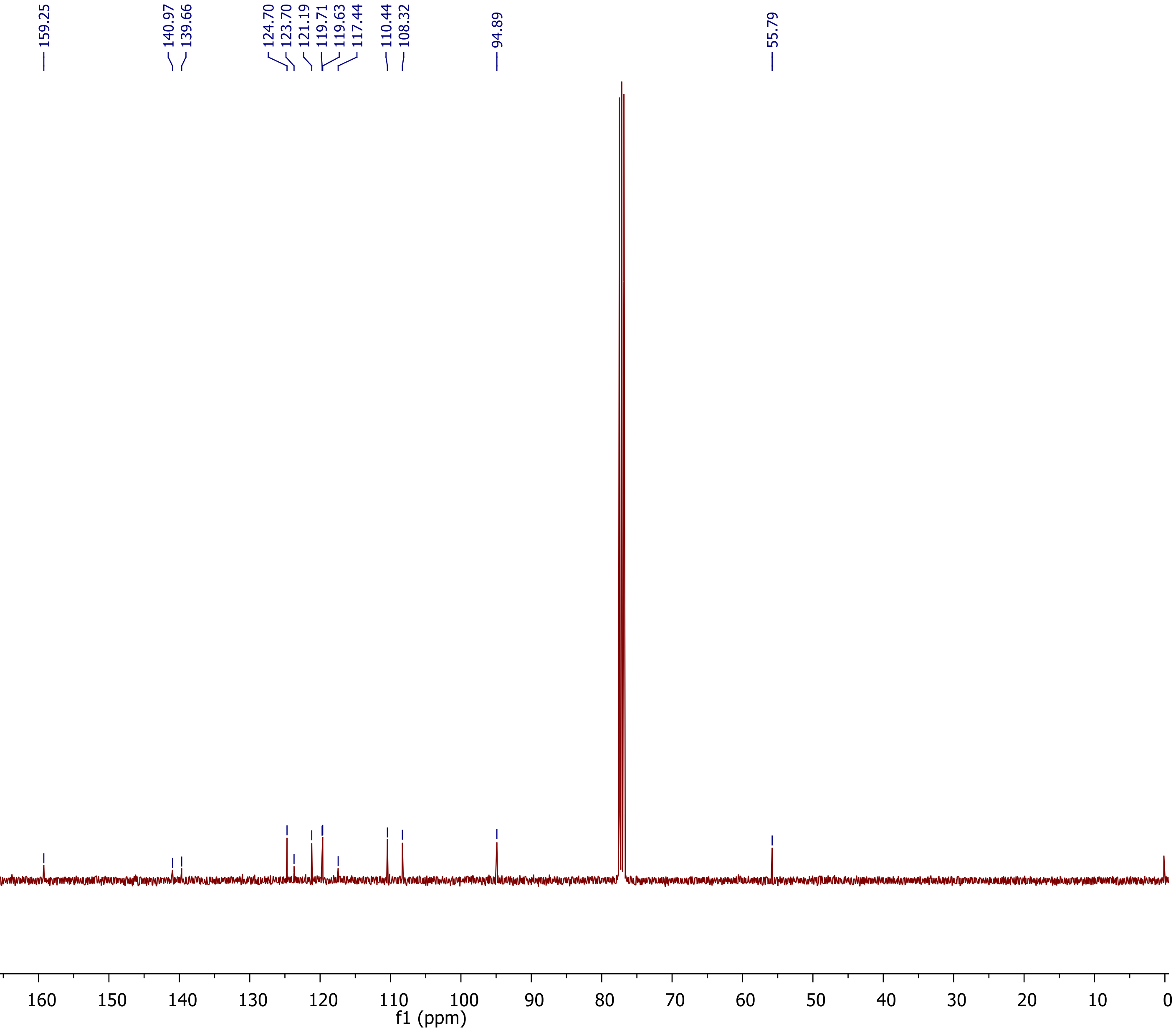

^{1}H NMR (400 MHz, $CDCl_3$)

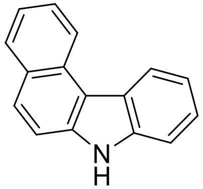

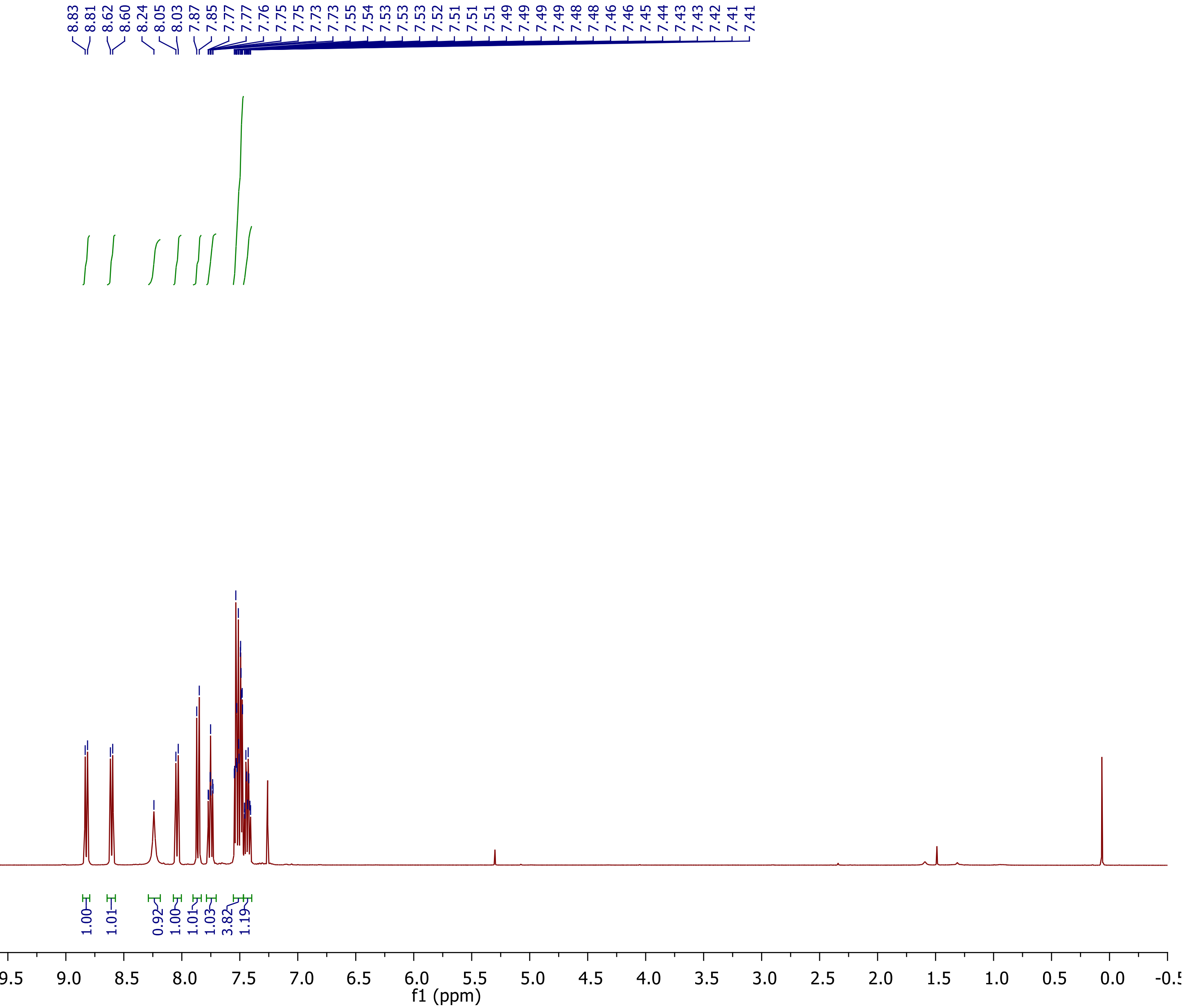

$^{13}C\{^{1}H\}$ NMR (101 MHz, $CDCl_3$)

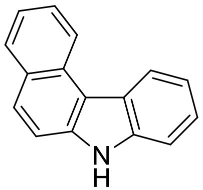

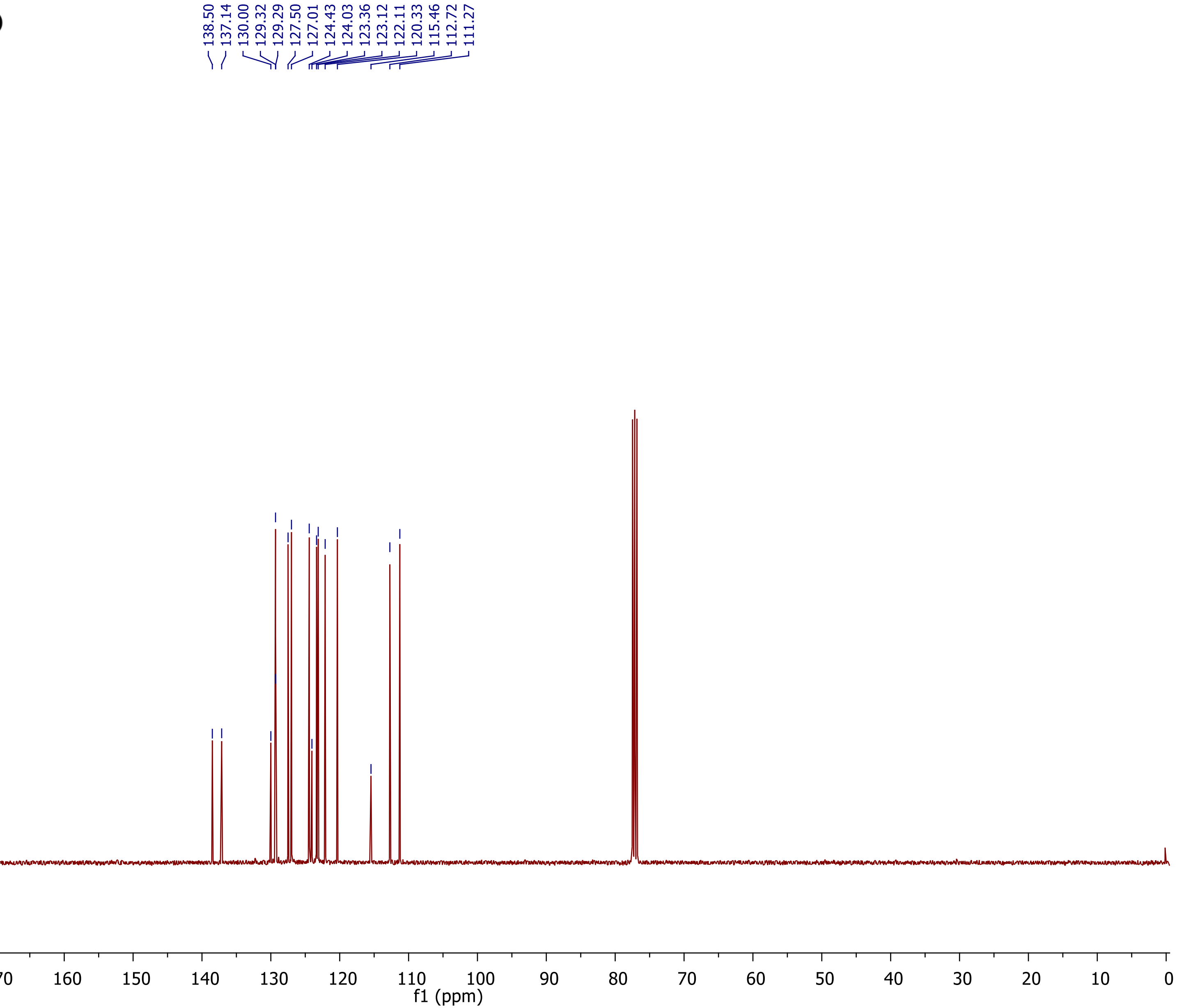

^{1}H NMR (400 MHz, $CDCl_3$)

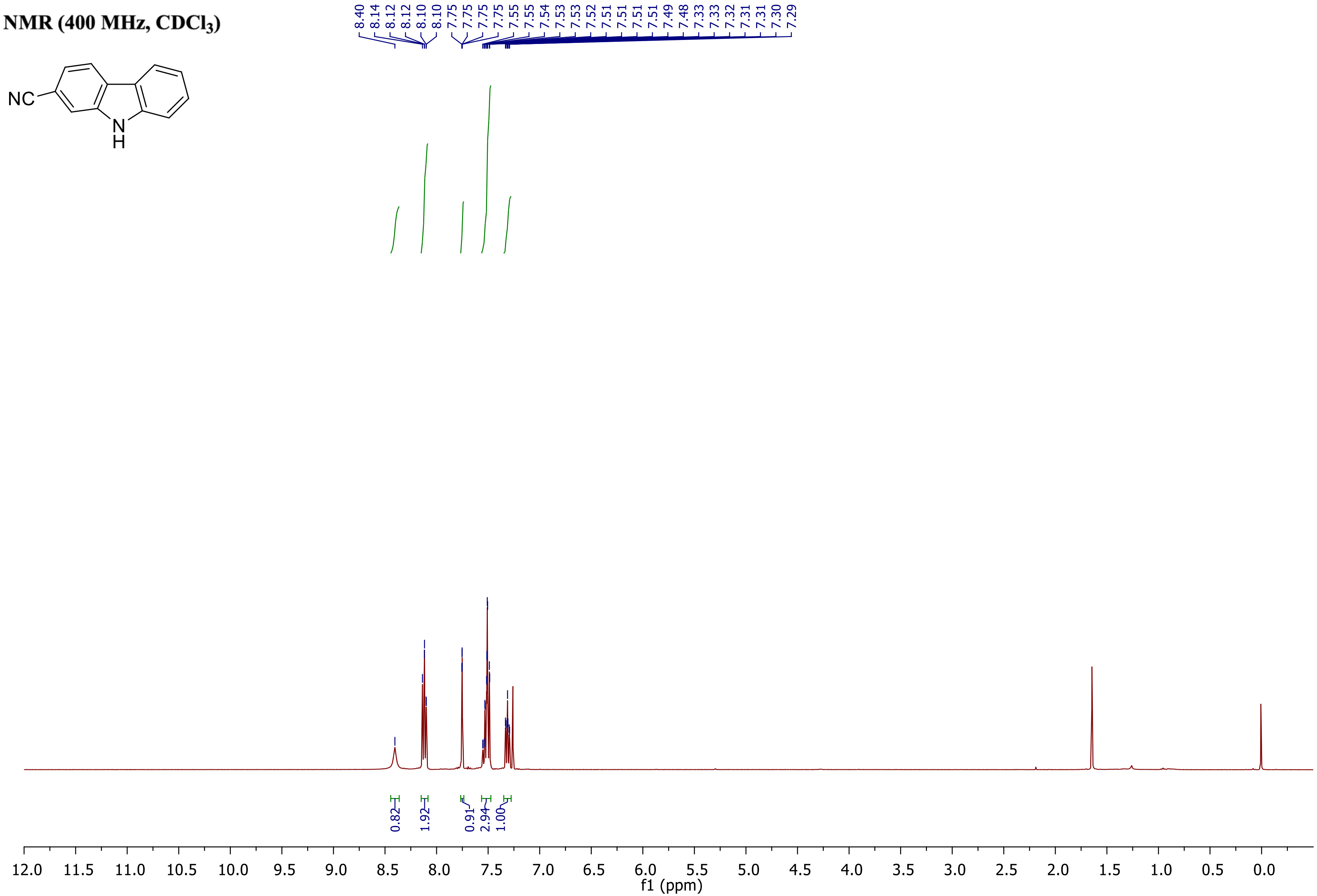

$^{13}C\{^1H\}$ NMR (101 MHz, $CDCl_3$)

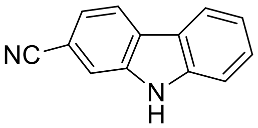

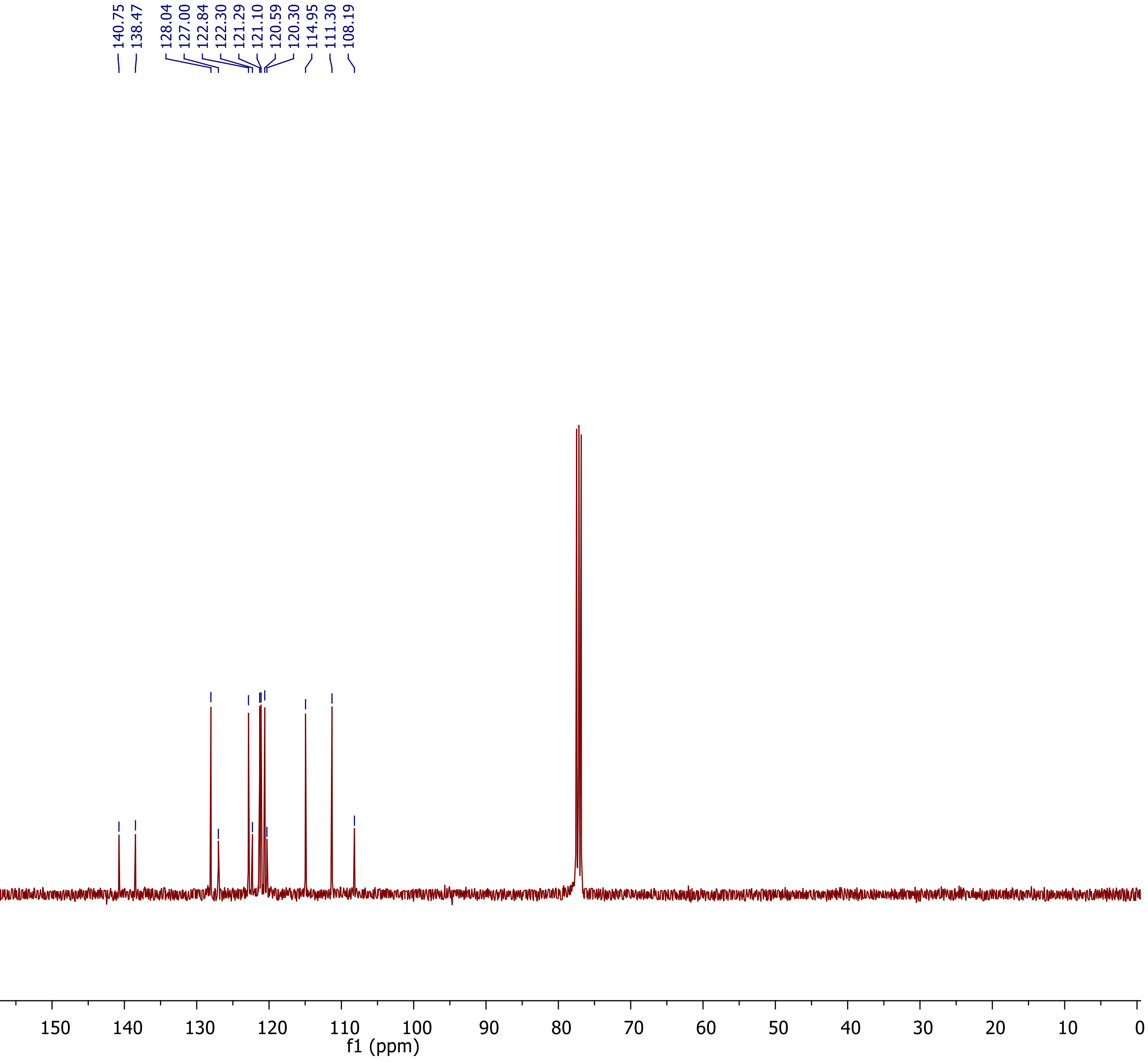

^{1}H NMR (400 MHz, $CDCl_3$)

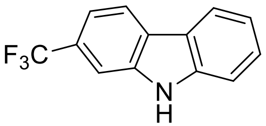

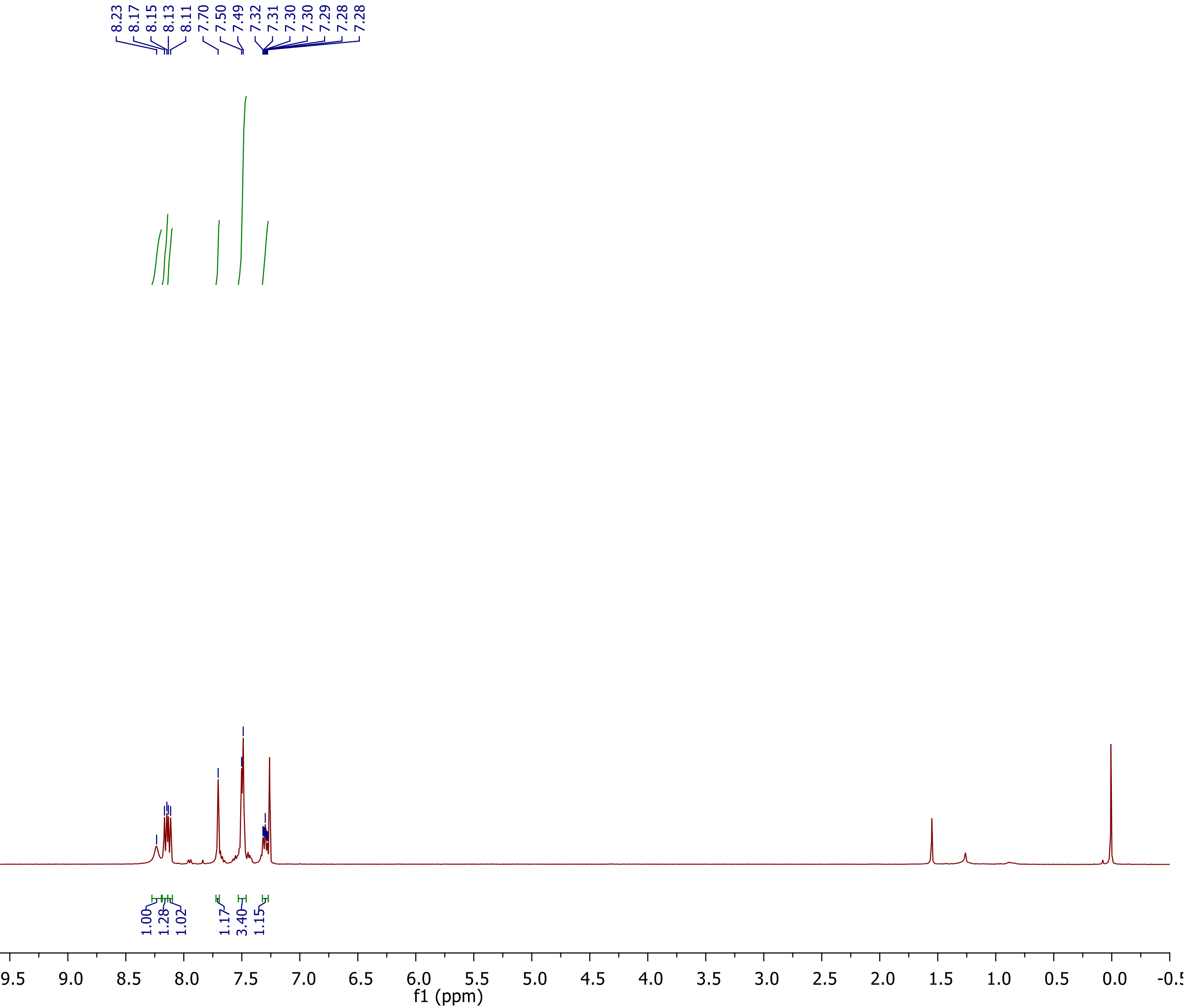

$^{13}C\{^{1}H\}$ NMR (101 MHz, $CDCl_3$)

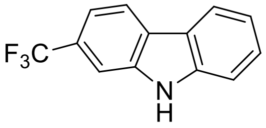

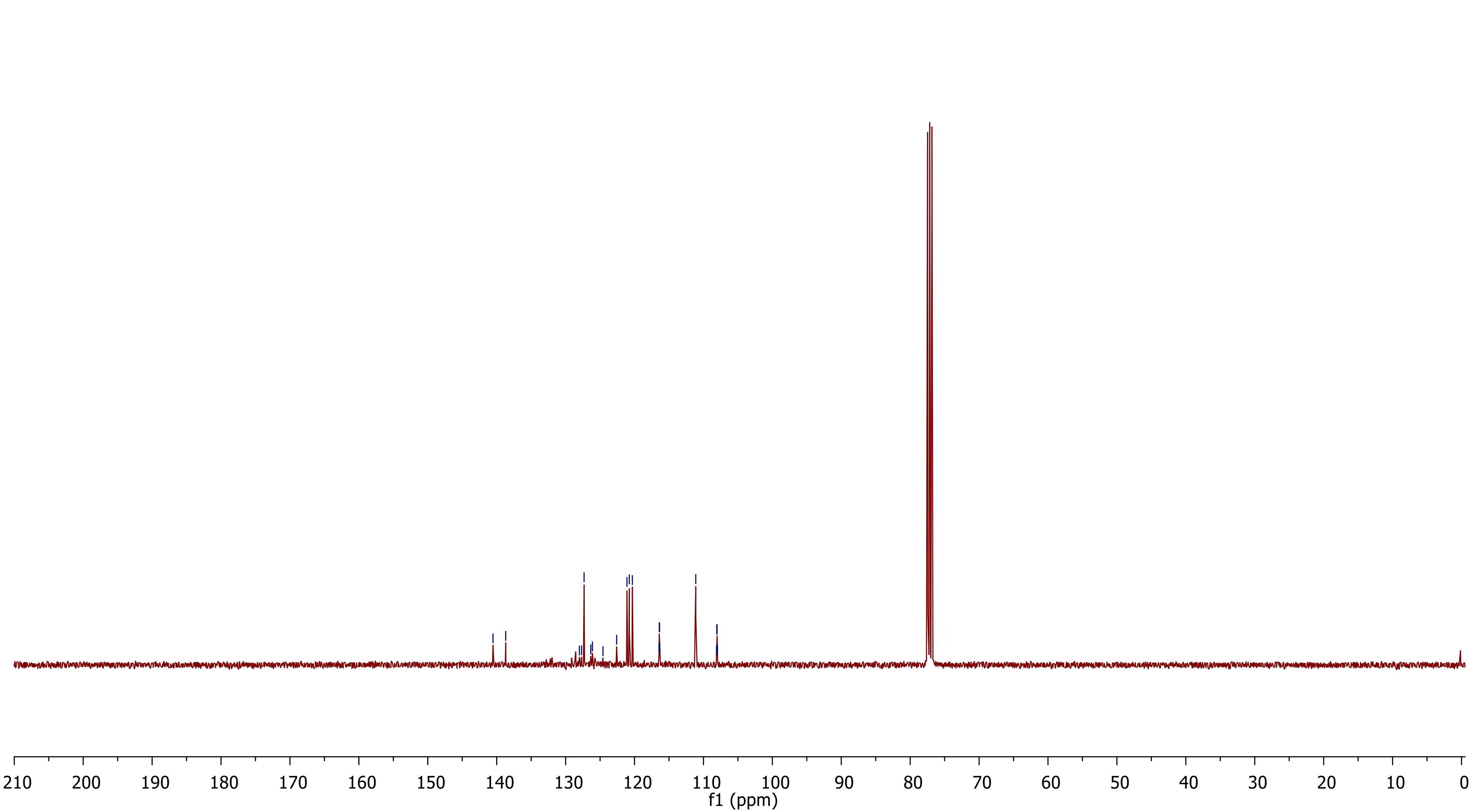

^{1}H NMR (400 MHz, $CDCl_3$)

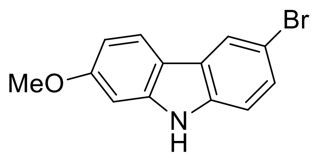

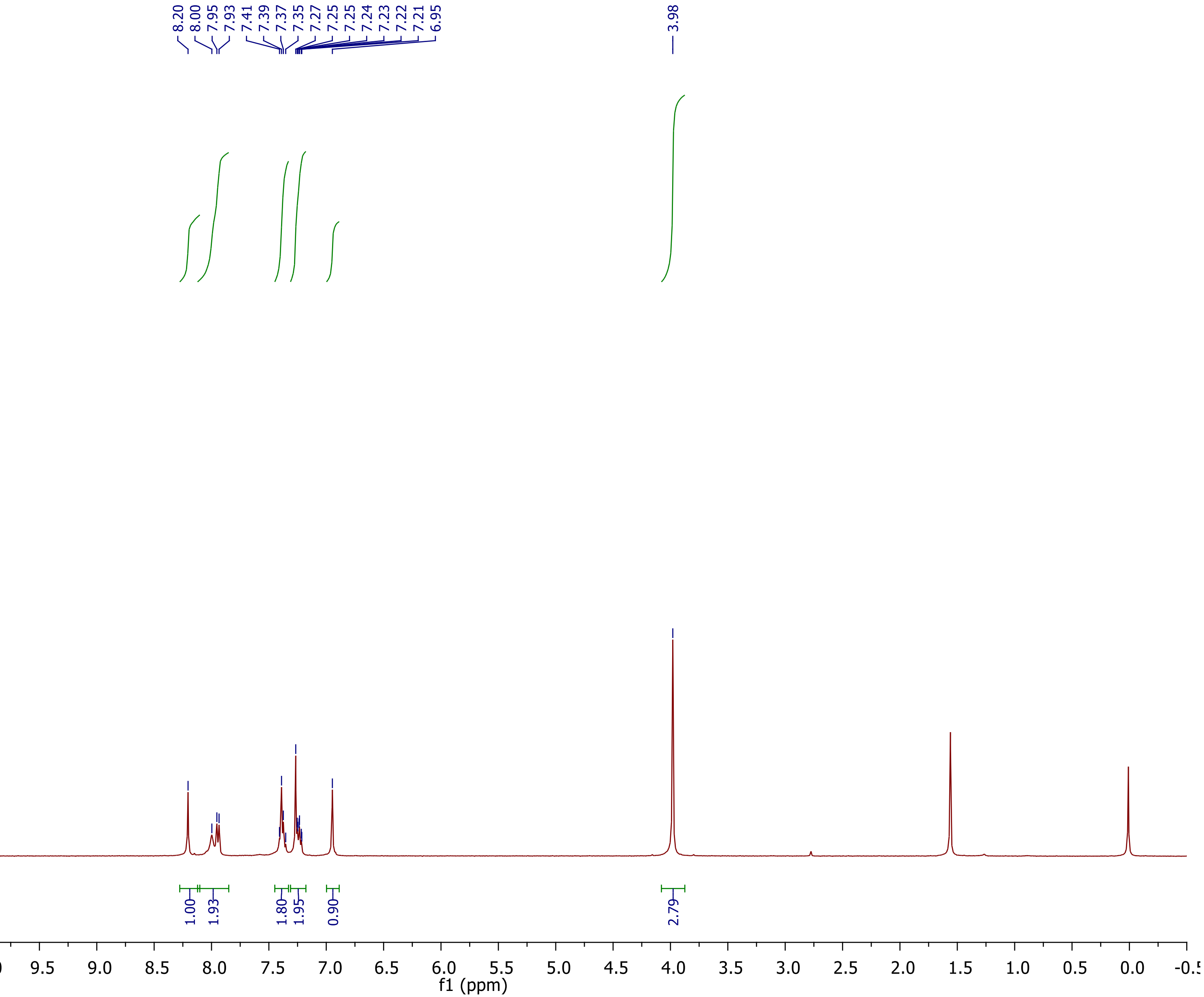

$^{13}C\{^{1}H\}$ NMR (101 MHz, $CDCl_3$)

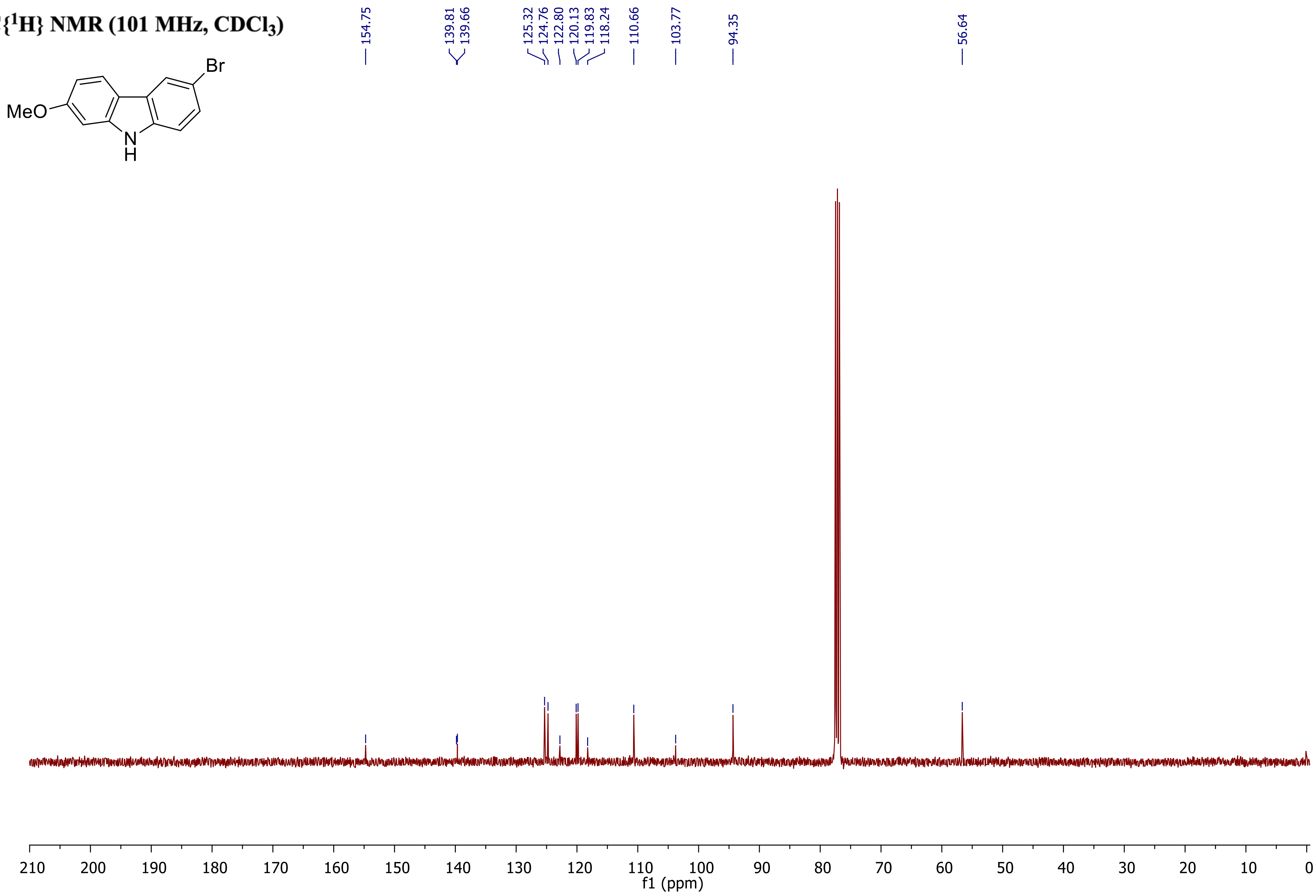

^{1}H NMR (400 MHz, $CDCl_3$)

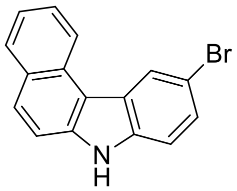

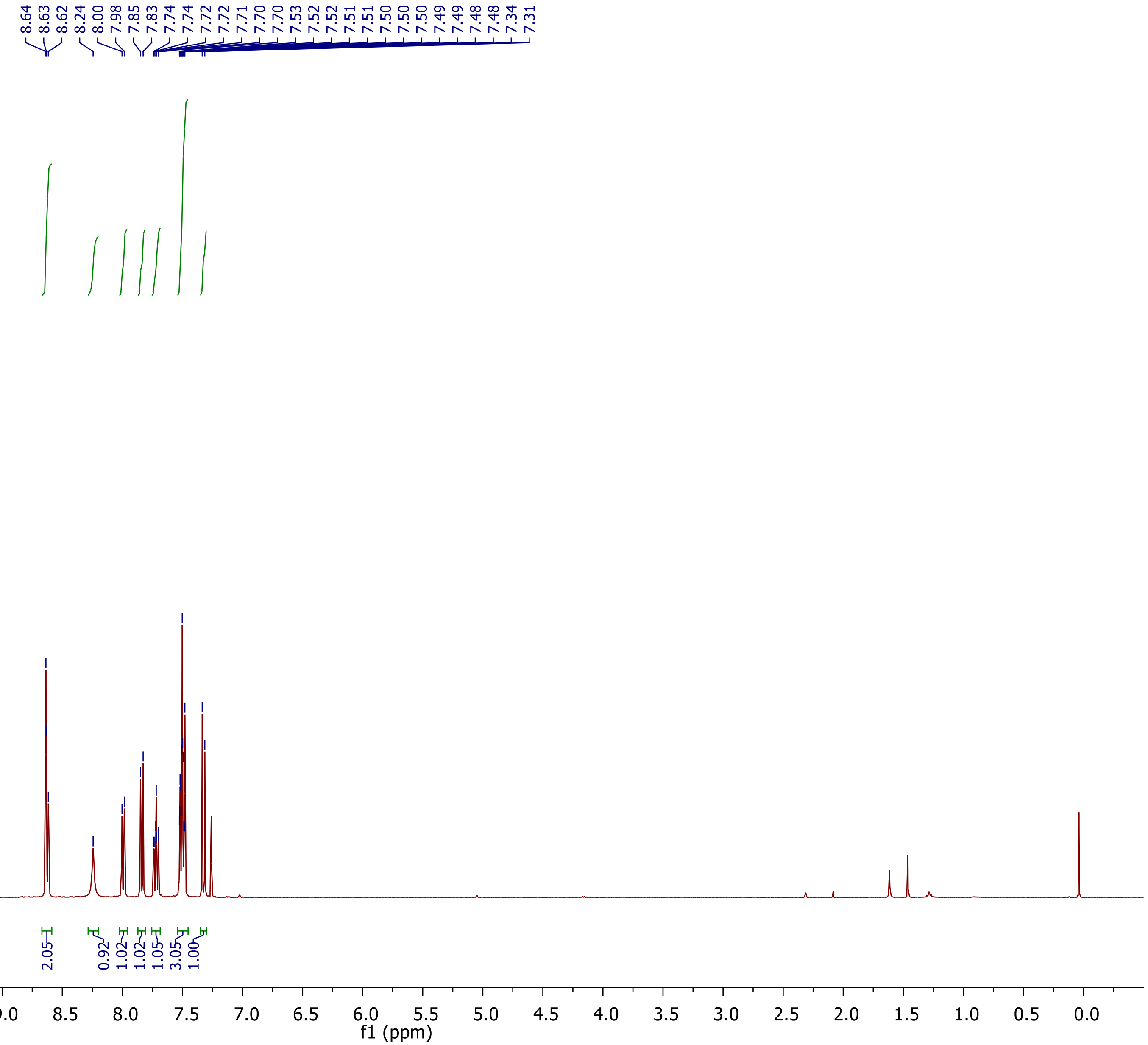

$^{13}C\{^1H\}$ NMR (101 MHz, $CDCl_3$)

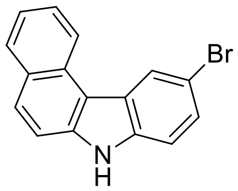

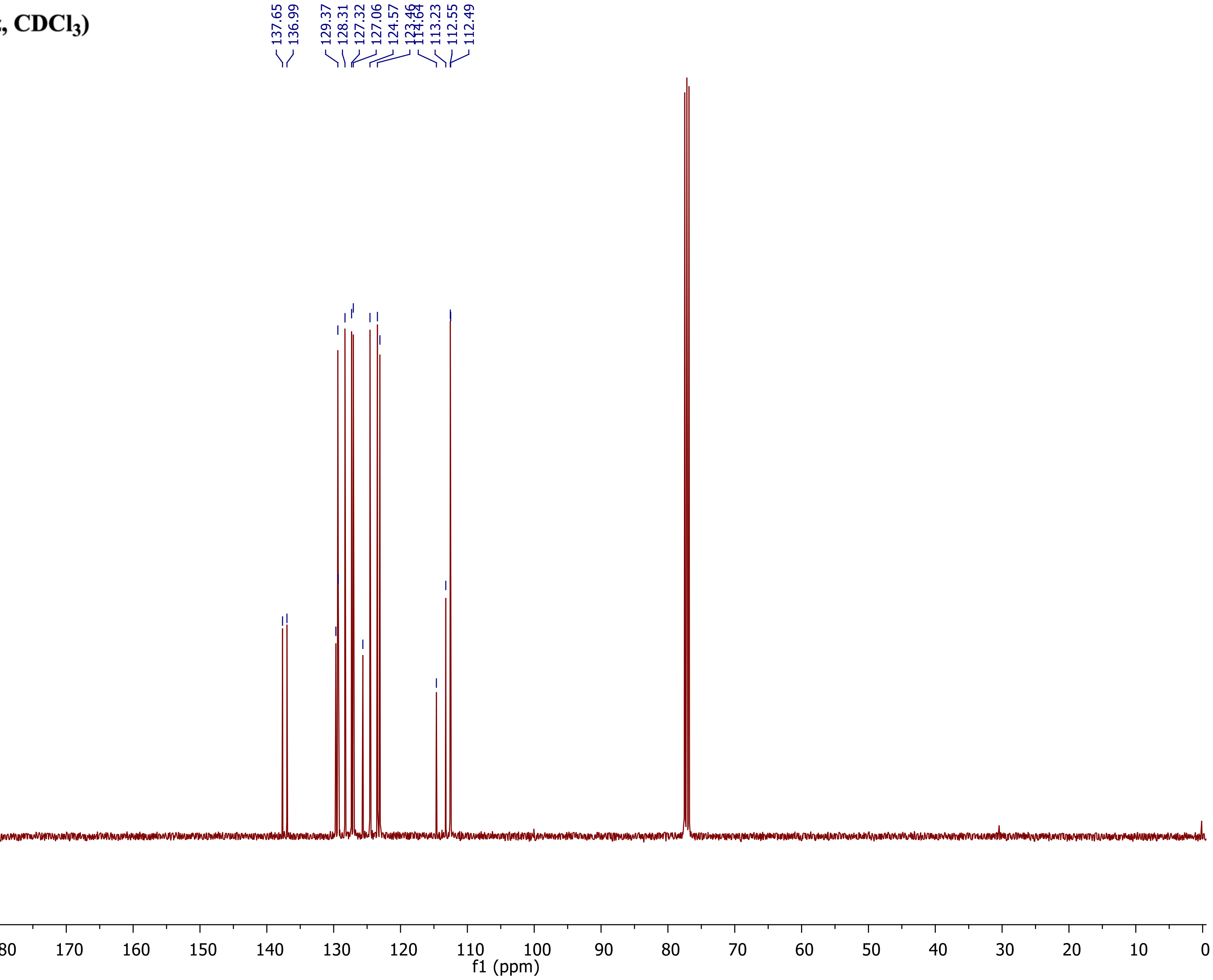

^{1}H NMR (400 MHz, DMSO-d_6)

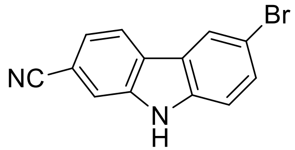

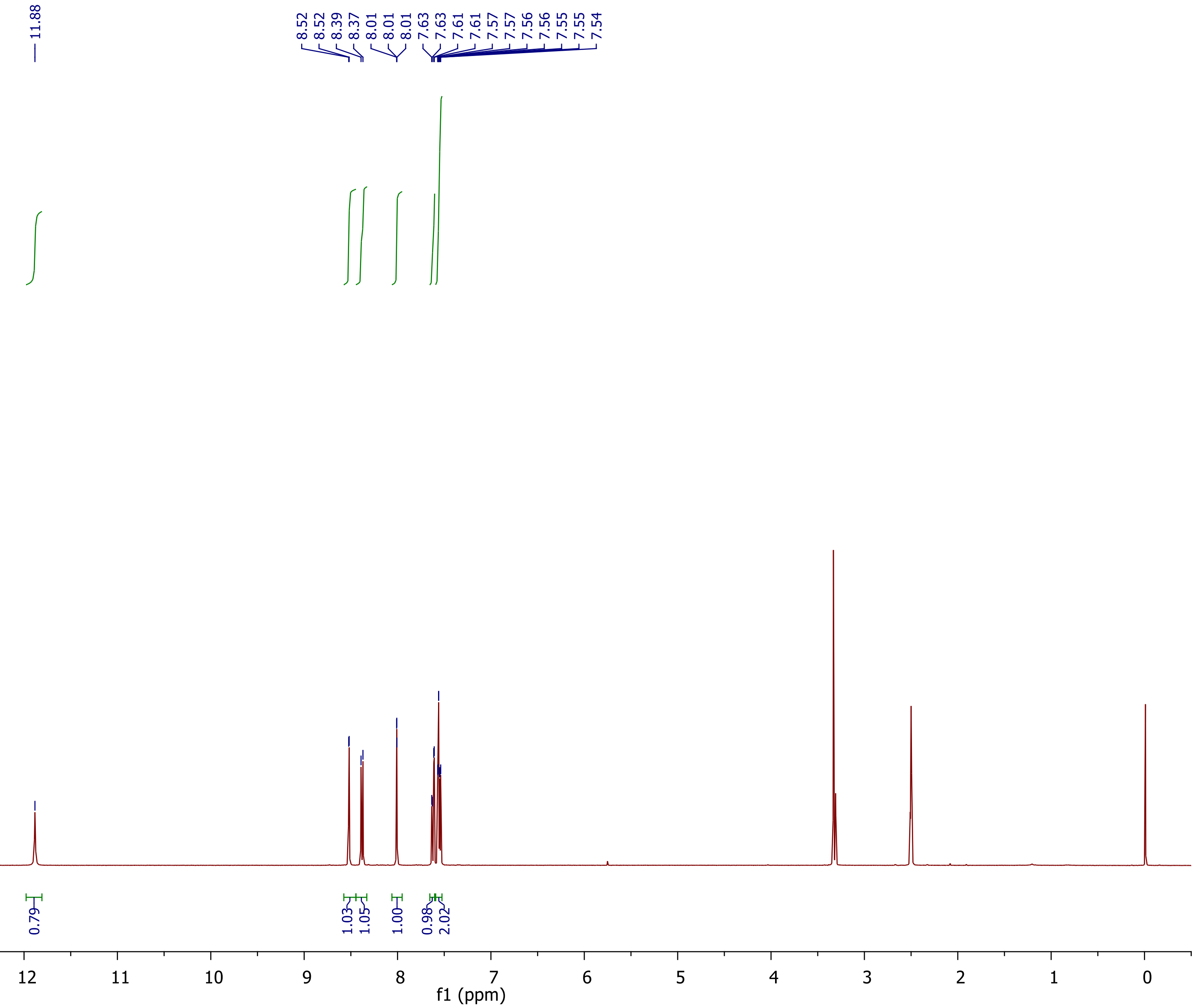

$^{13}C\{^{1}H\}$ NMR (101 MHz, DMSO-d_6)

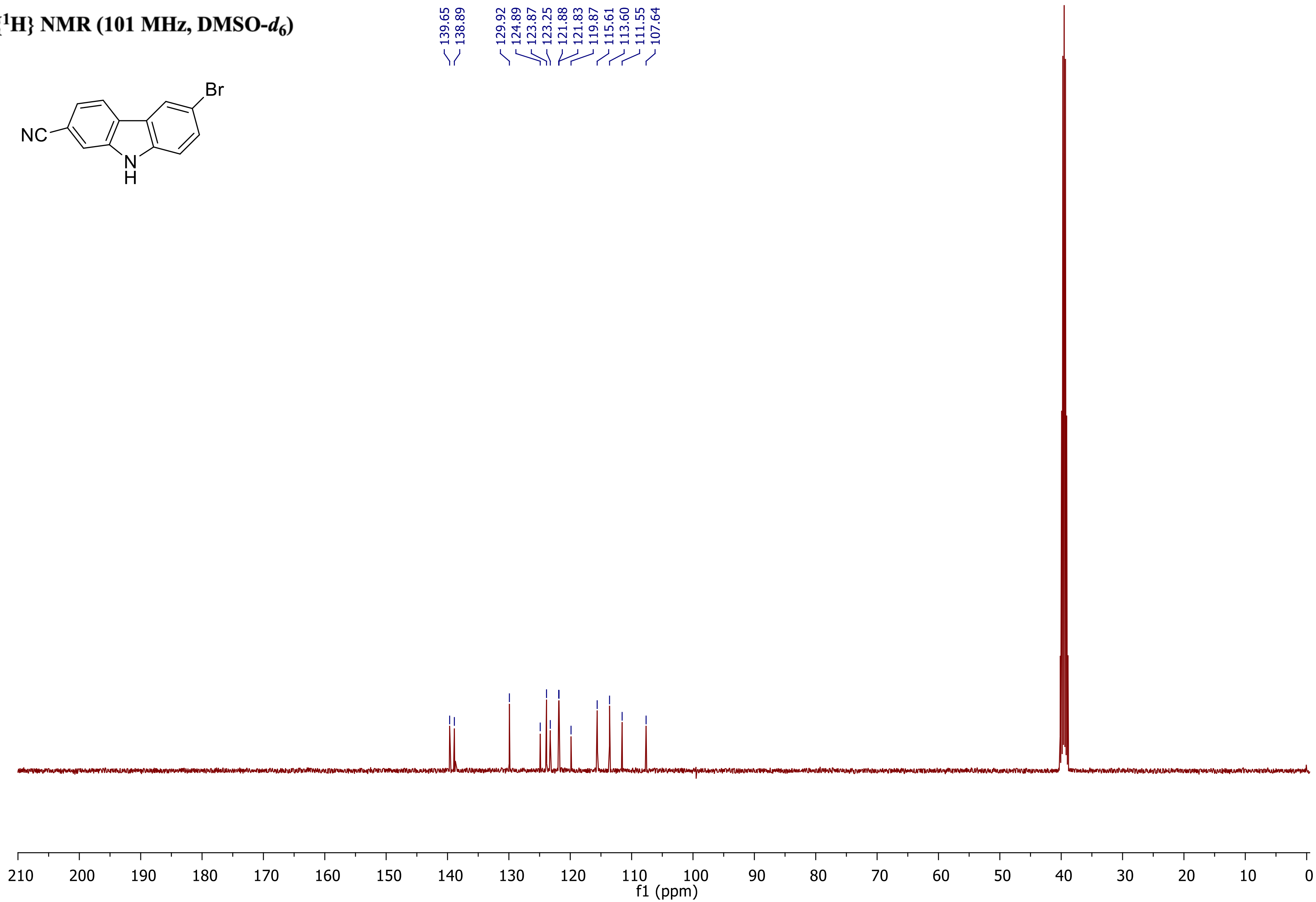

^{1}H NMR (400 MHz, $CDCl_3$)

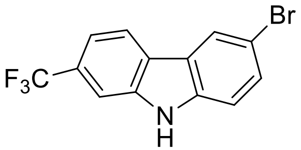

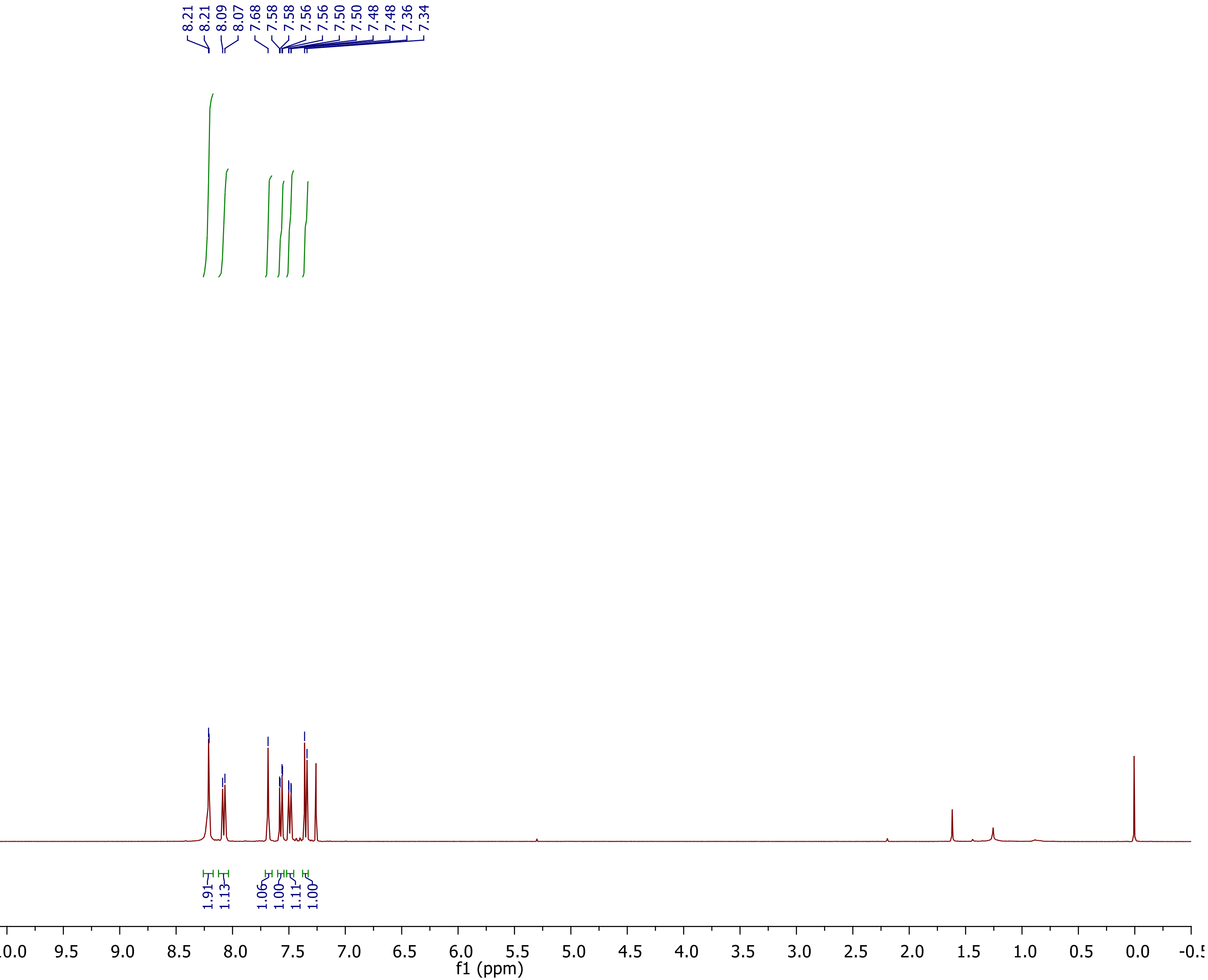

$^{13}C\{^{1}H\}$ NMR (101 MHz, $CDCl_3$)

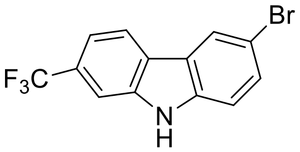

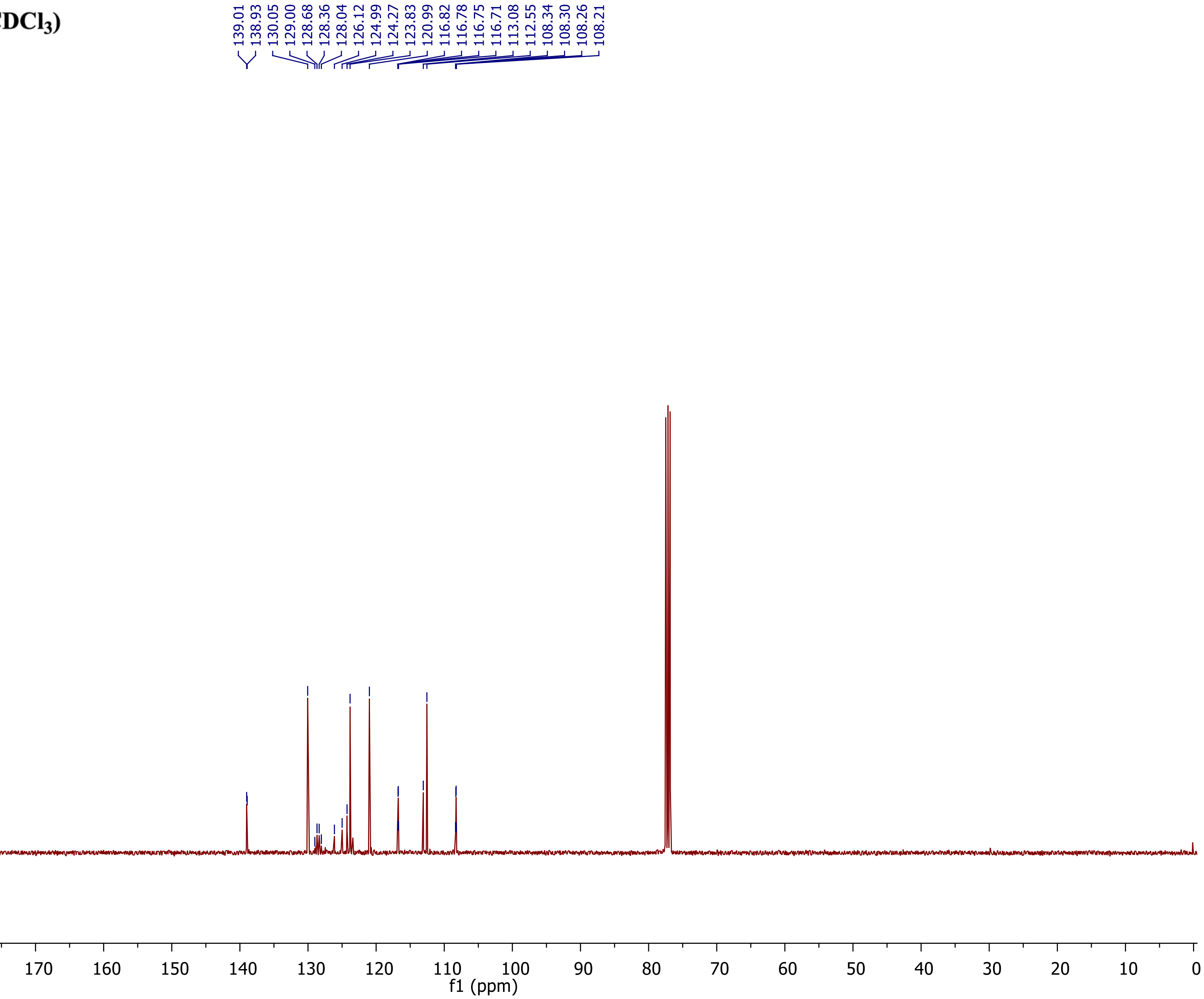

^{1}H NMR (500 MHz, CDCl$_3$)

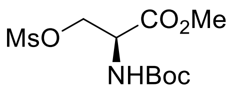

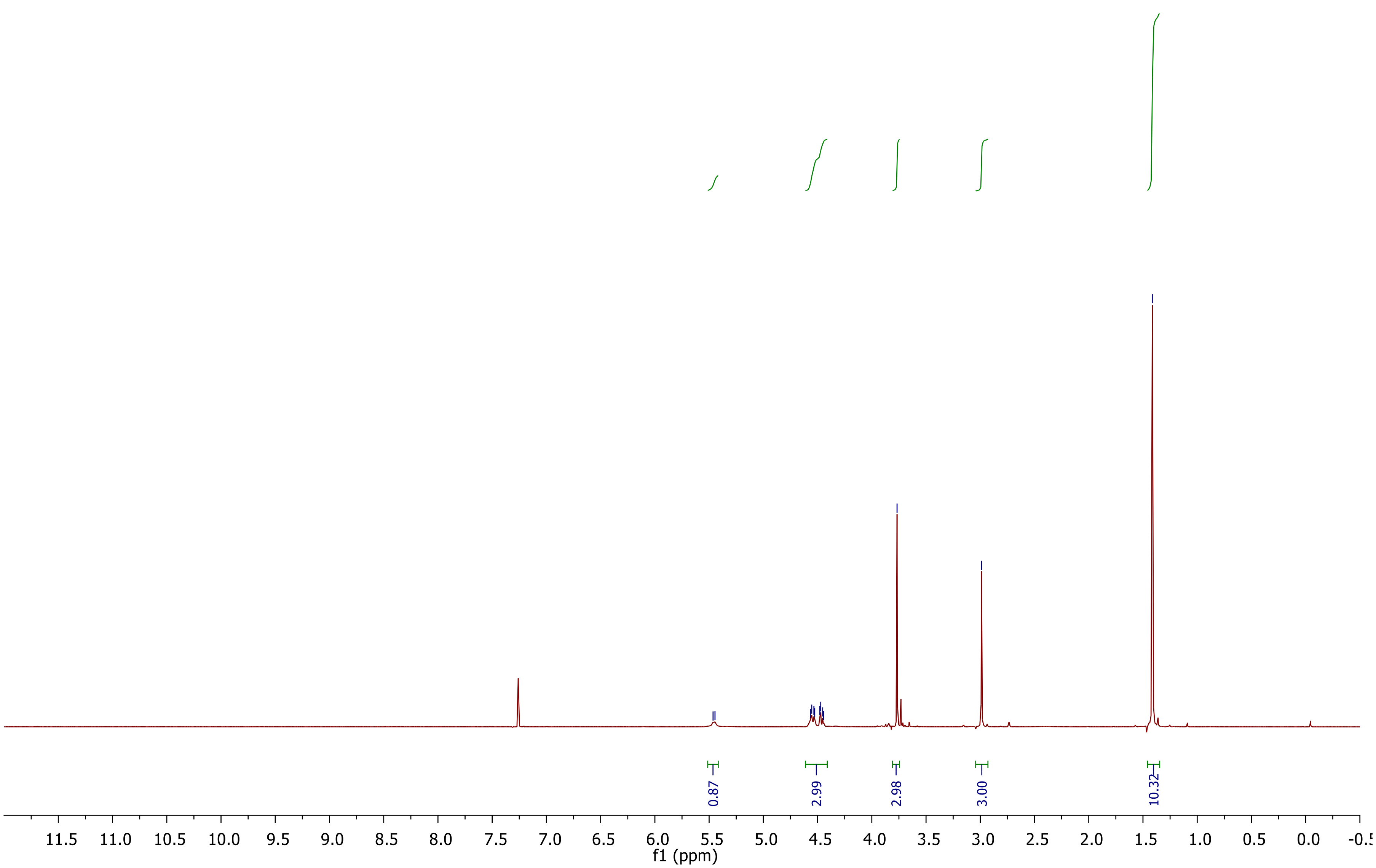

$^{13}C\{^1H\}$ NMR (101 MHz, $CDCl_3$)

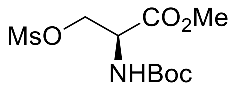

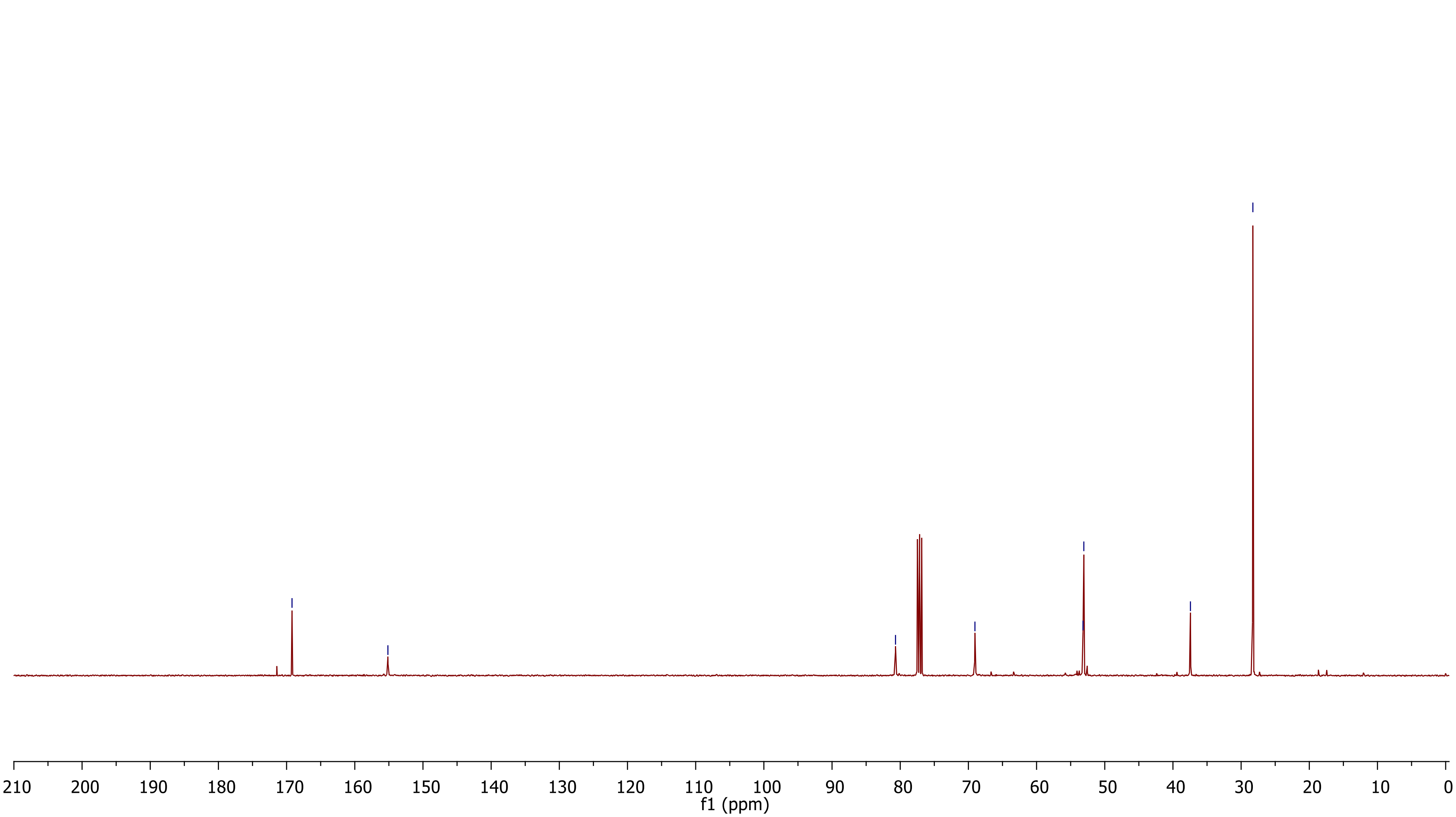

^{1}H NMR (400 MHz, $CDCl_3$)

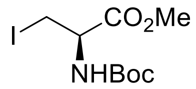

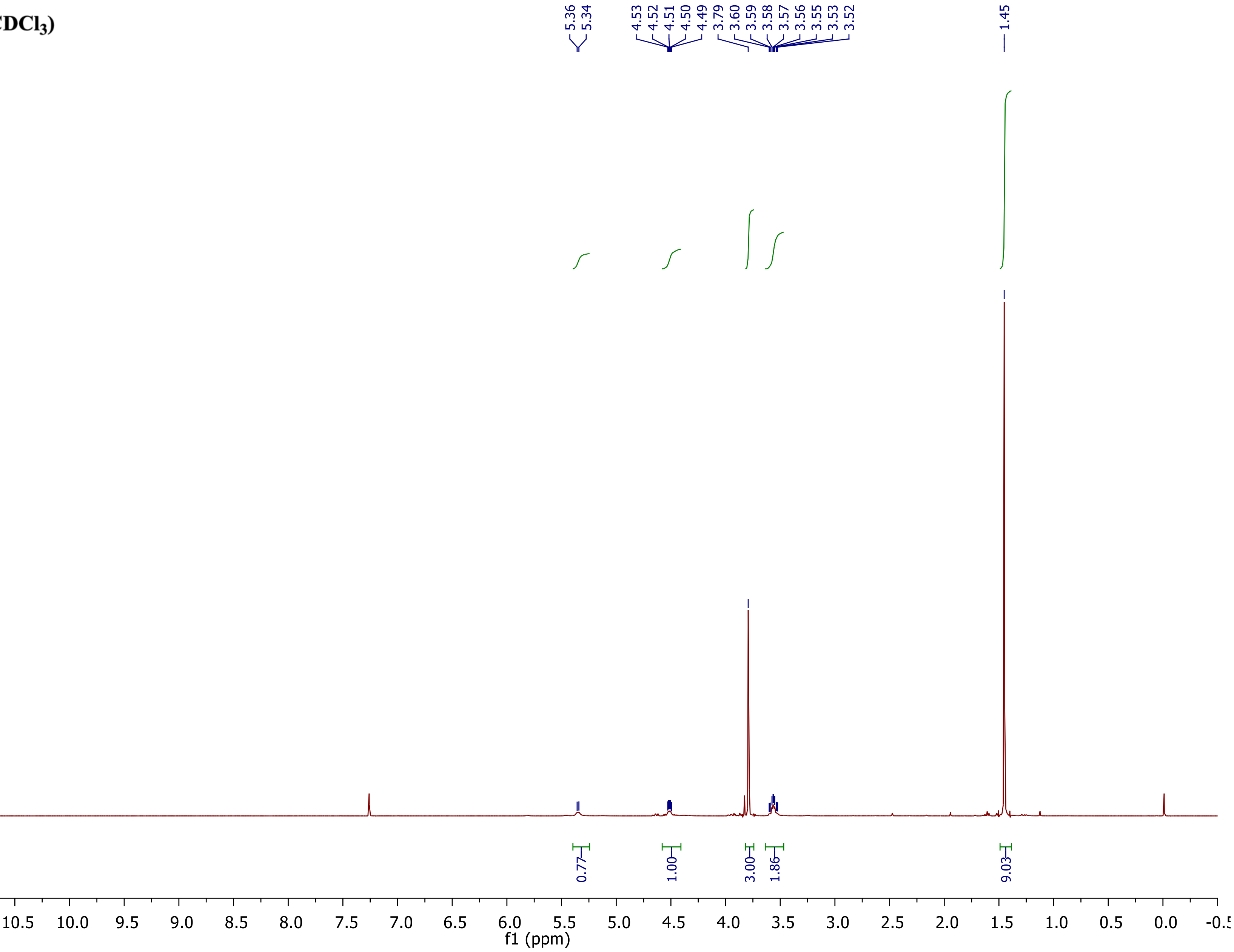

$^{13}C\{^1H\}$ NMR (101 MHz, $CDCl_3$)

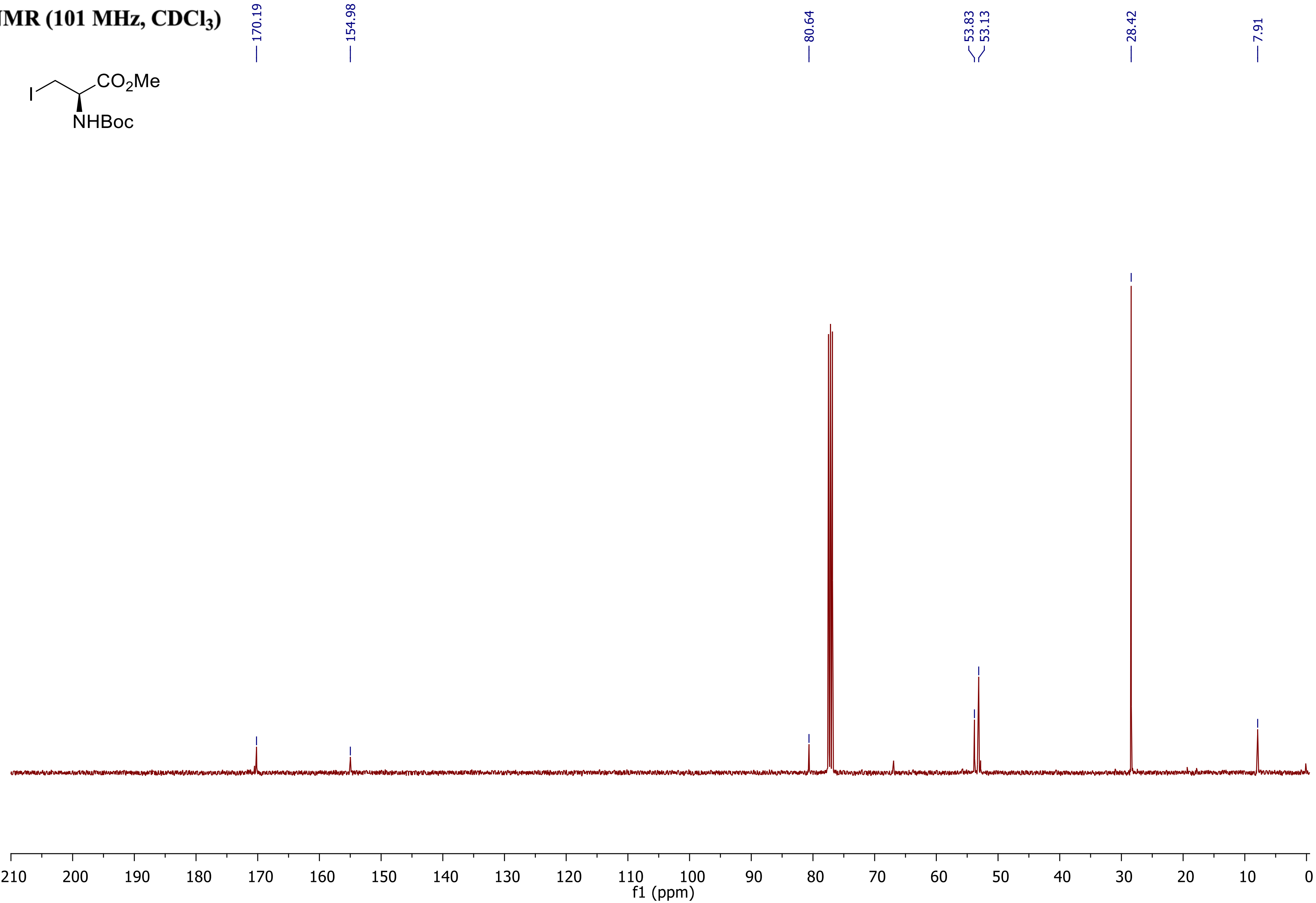

^{1}H NMR (400 MHz, $CDCl_3$)

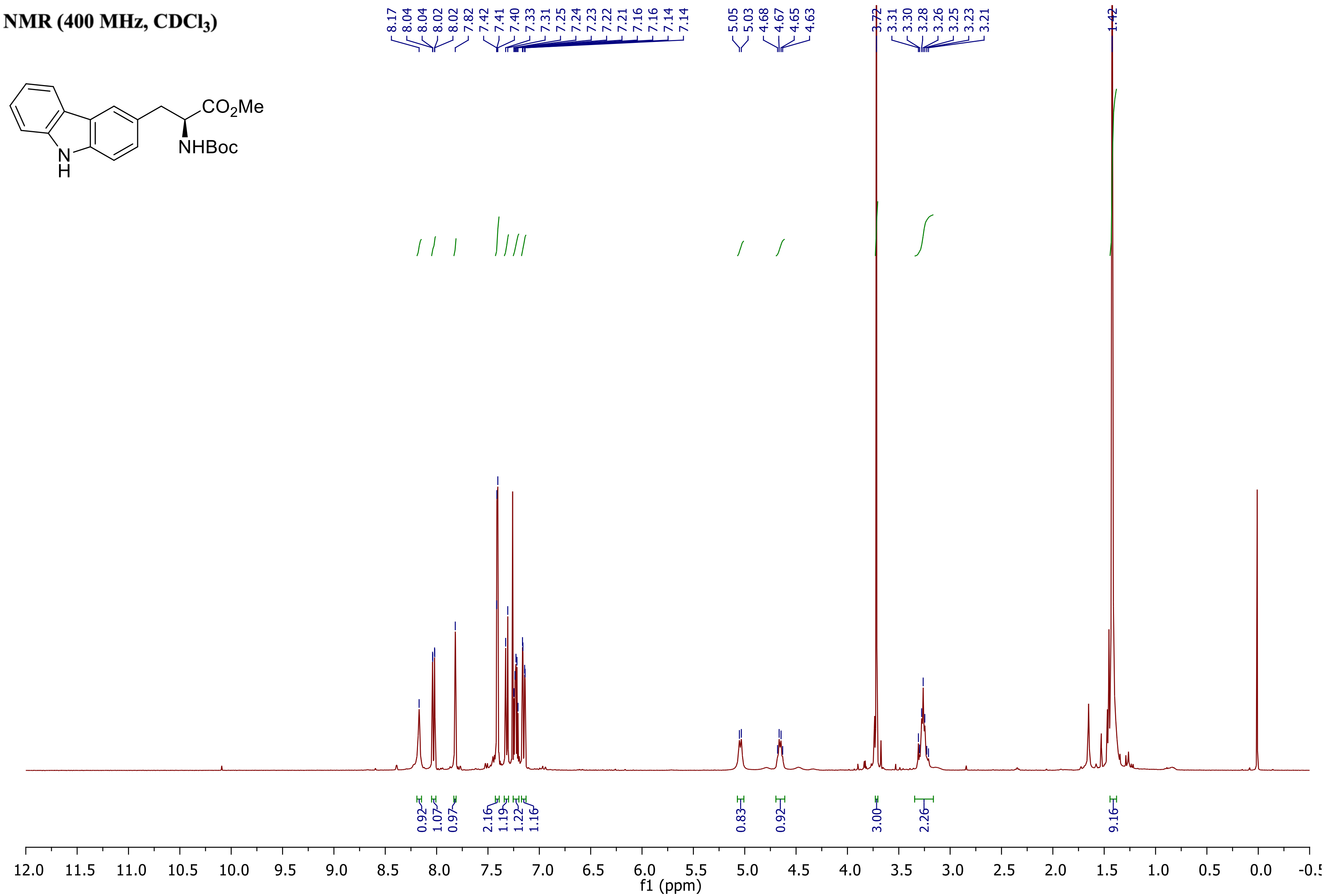

$^{13}C\{^1H\}$ NMR (101 MHz, $CDCl_3$)

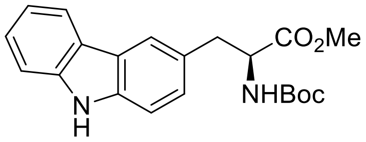

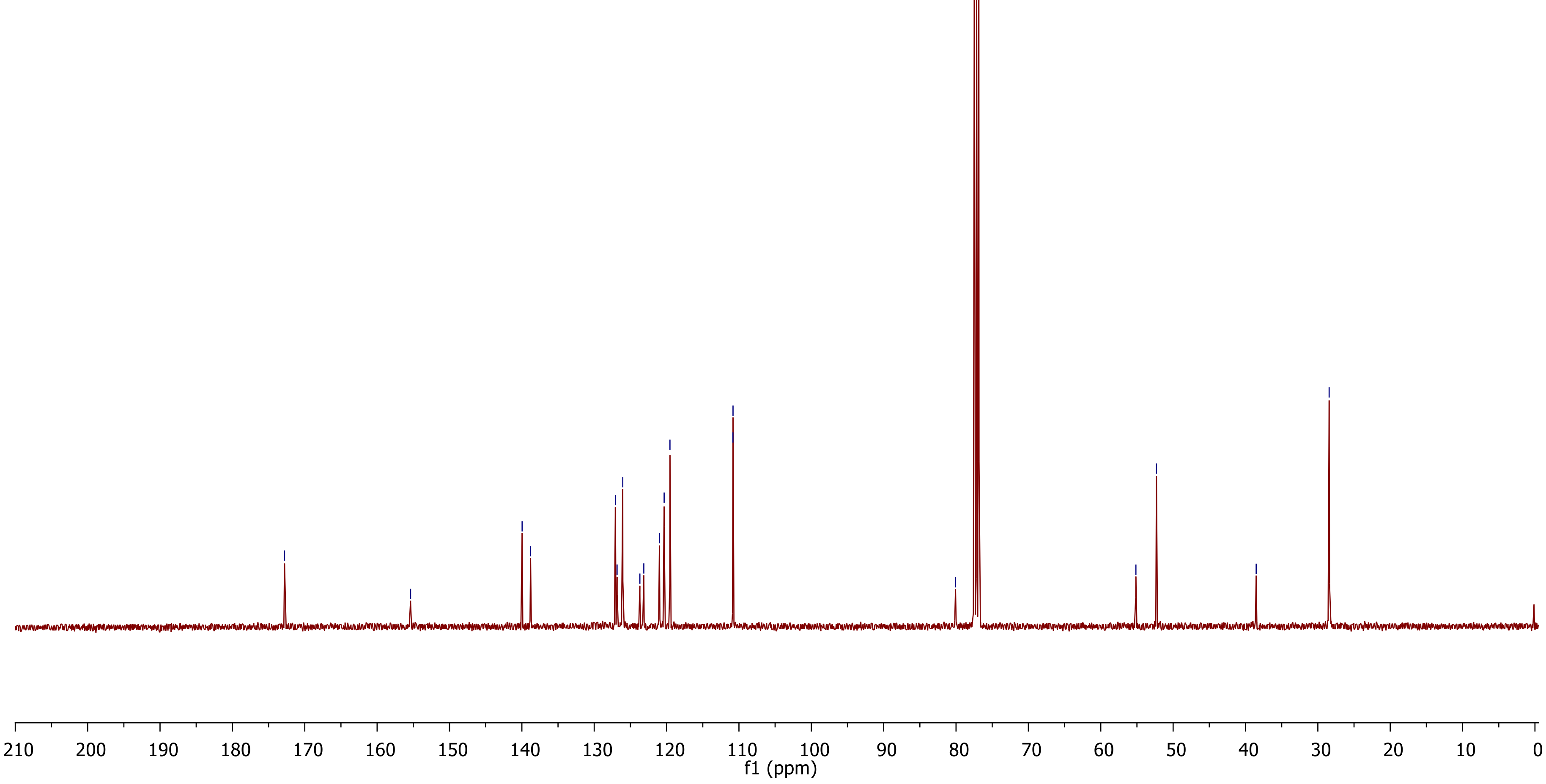

^{1}H NMR (400 MHz, $CDCl_3$)

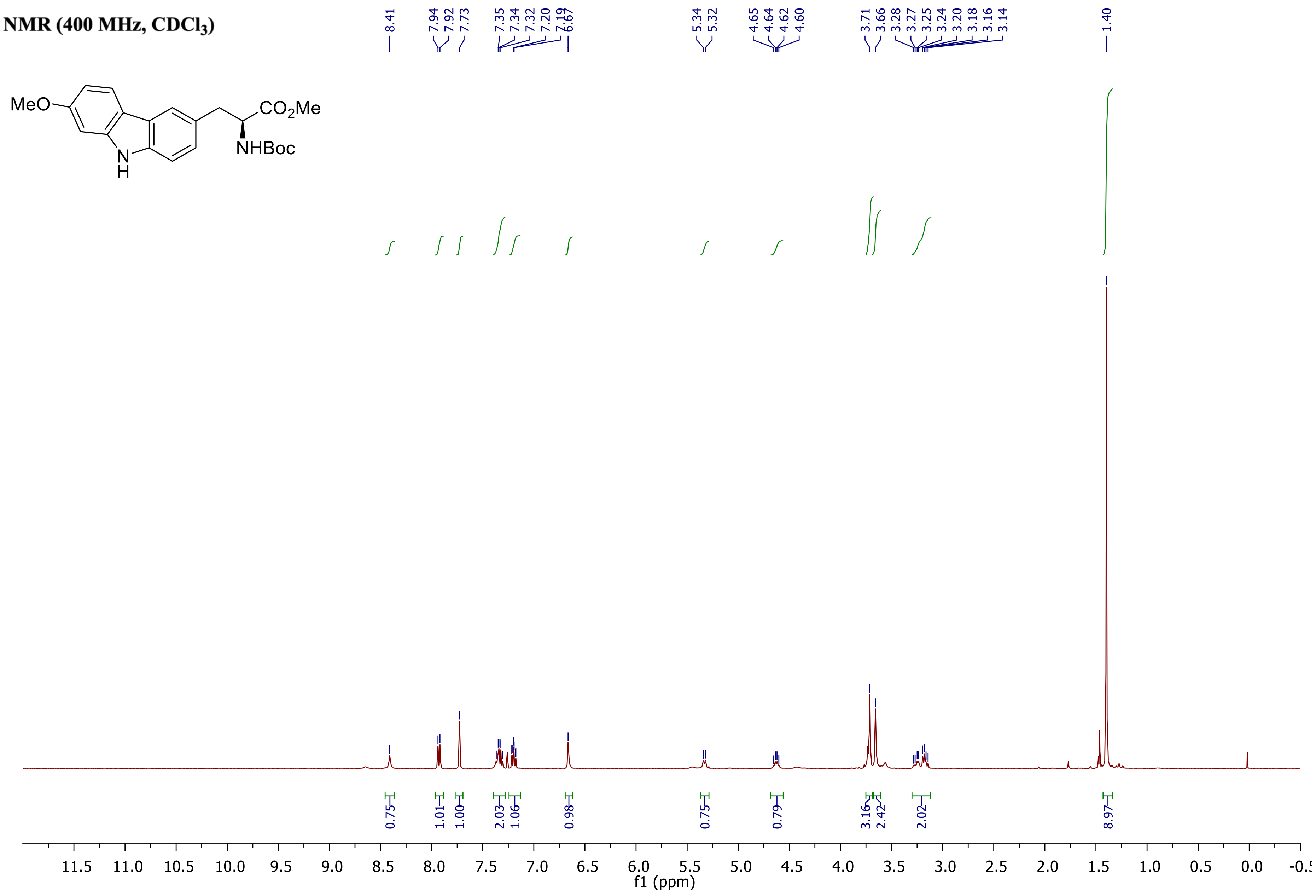

$^{13}C\{^1H\}$ NMR (101 MHz, $CDCl_3$)

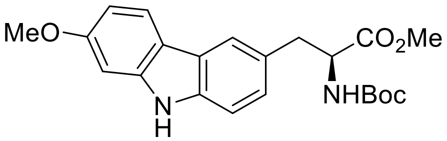

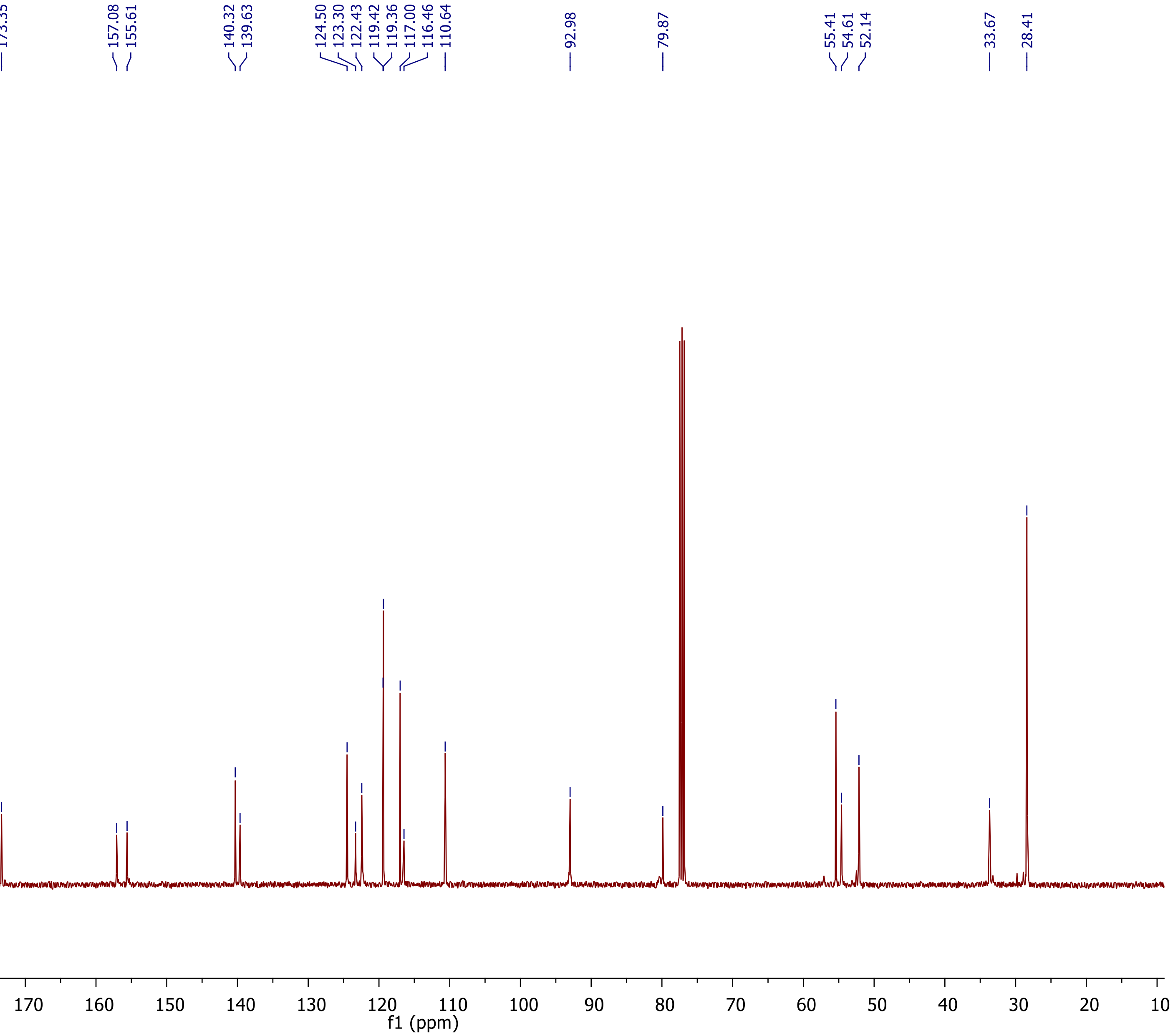

^{1}H NMR (400 MHz, $CDCl_3$)

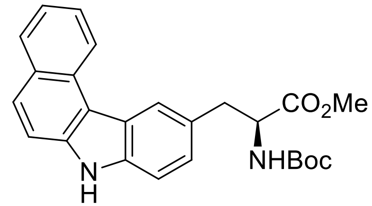

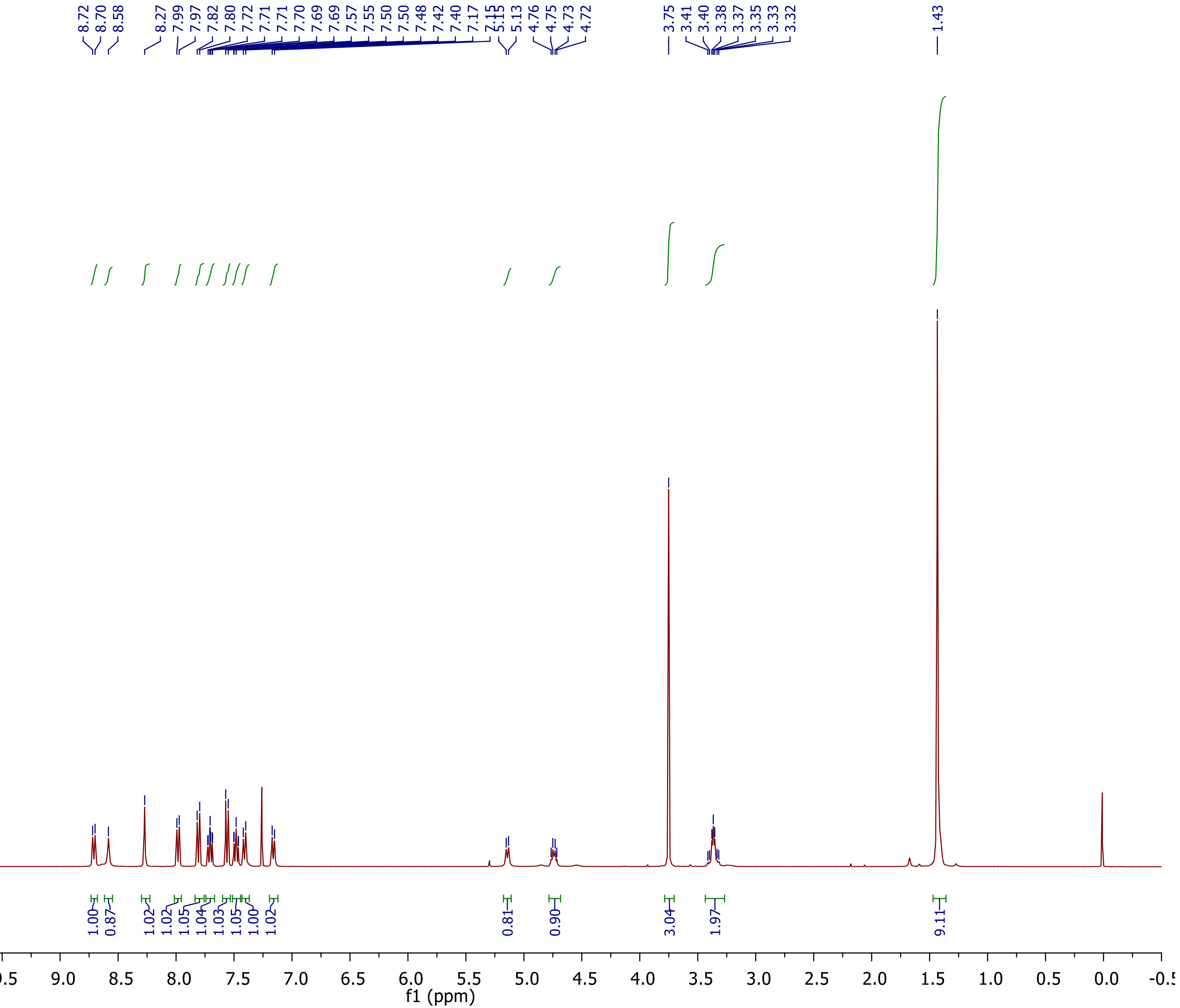

$^{13}C\{^{1}H\}$ NMR (101 MHz, $CDCl_3$)

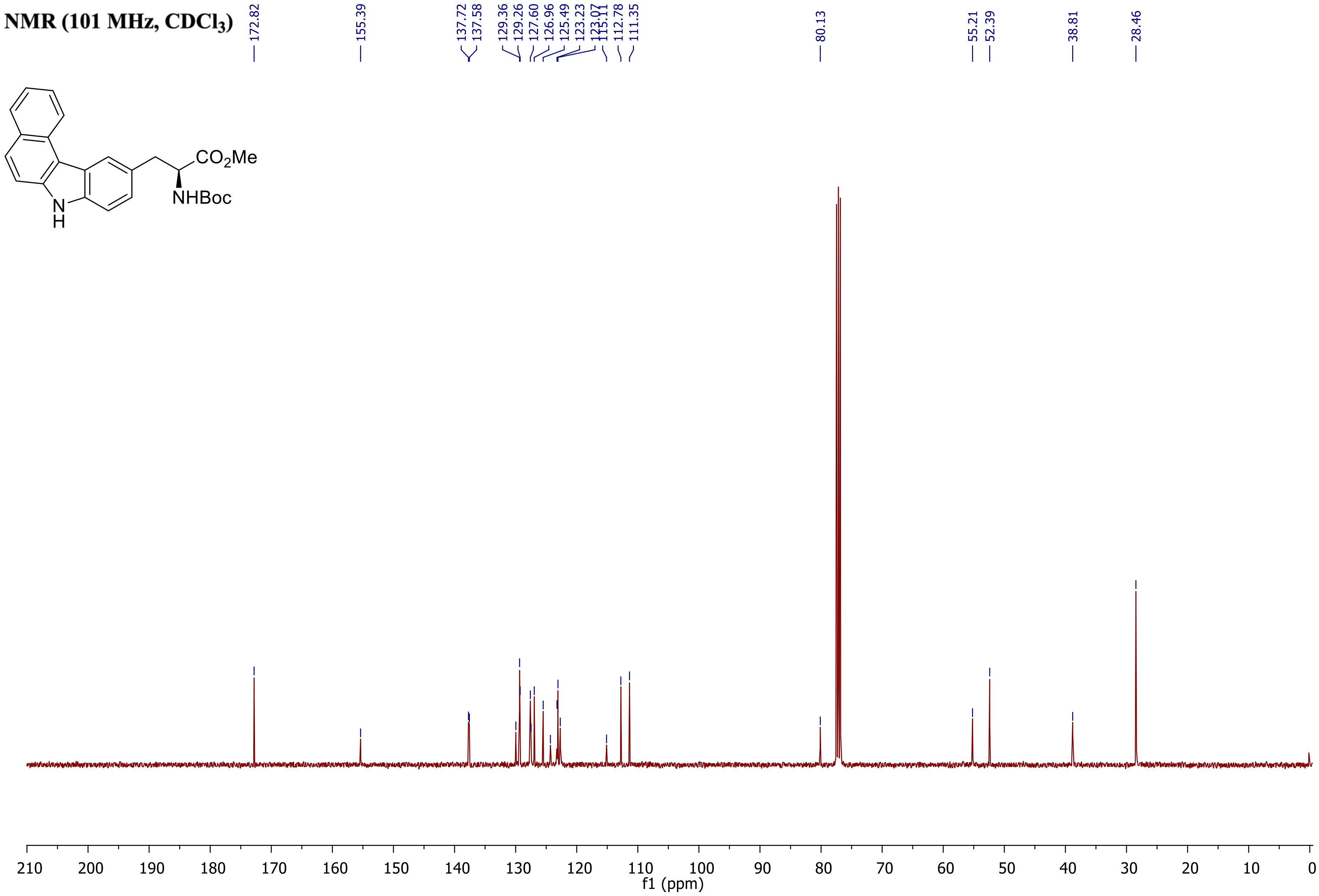

^1H NMR (400 MHz, $CDCl_3$)

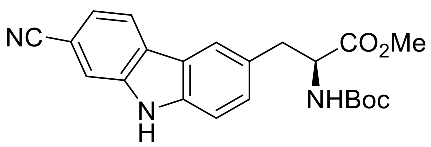

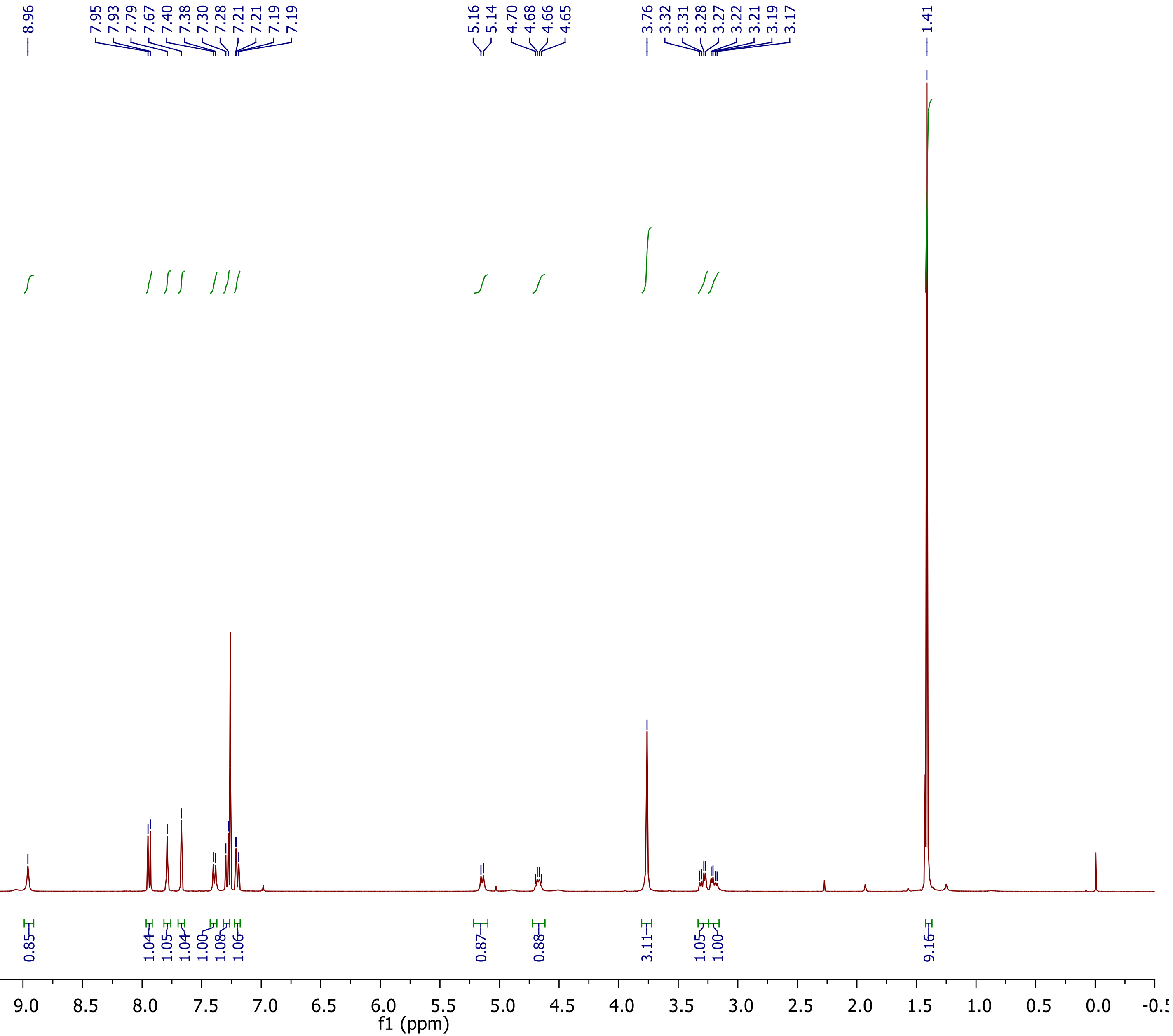

$^{13}C\{^{1}H\}$ **NMR (101 MHz, $CDCl_3$)**

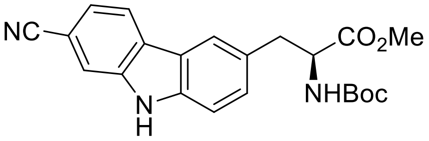

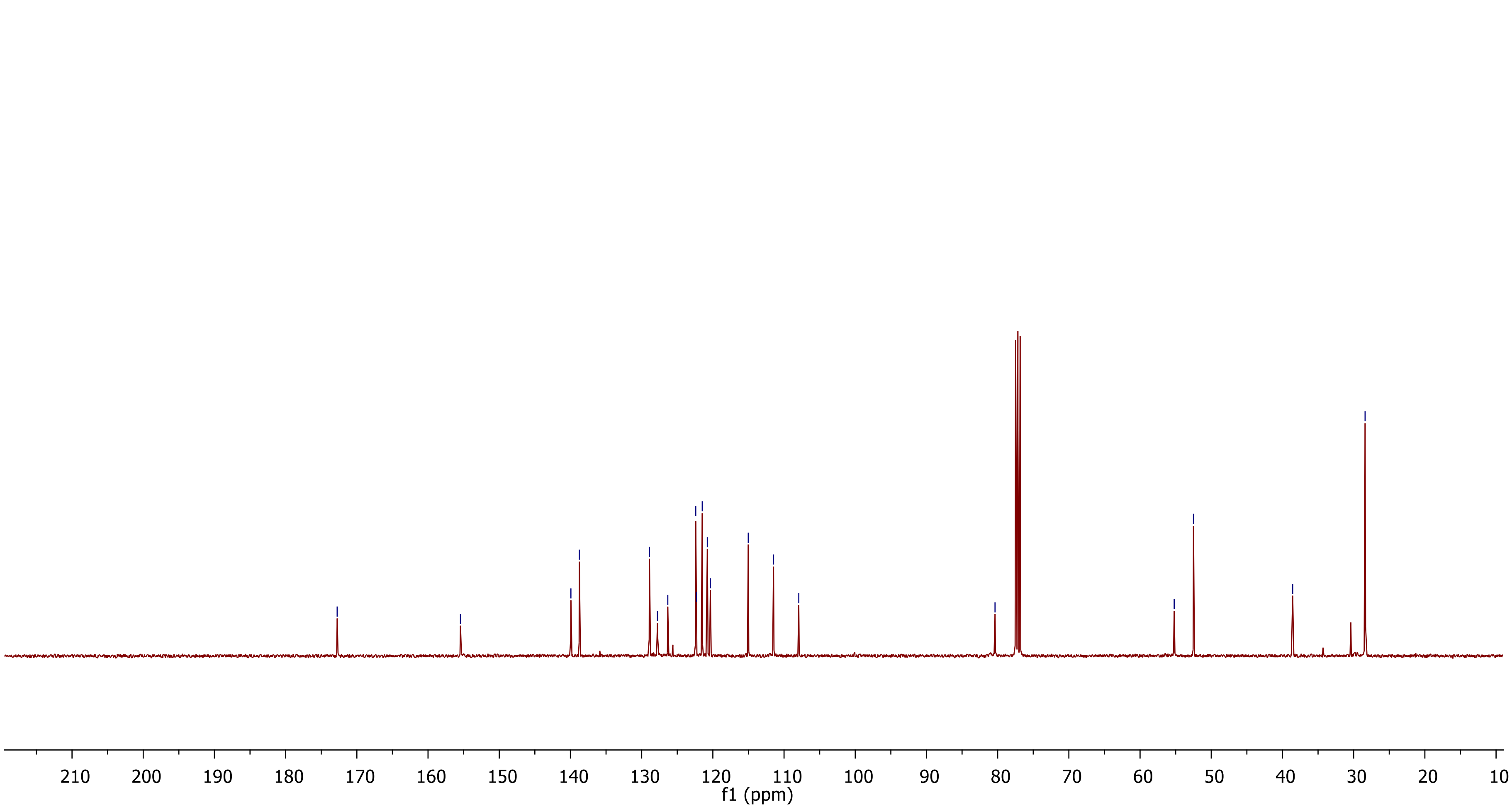

^{1}H NMR (400 MHz, CDCl$_3$)

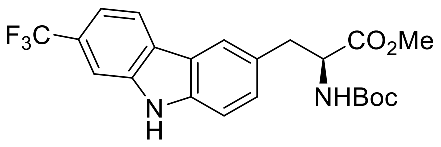

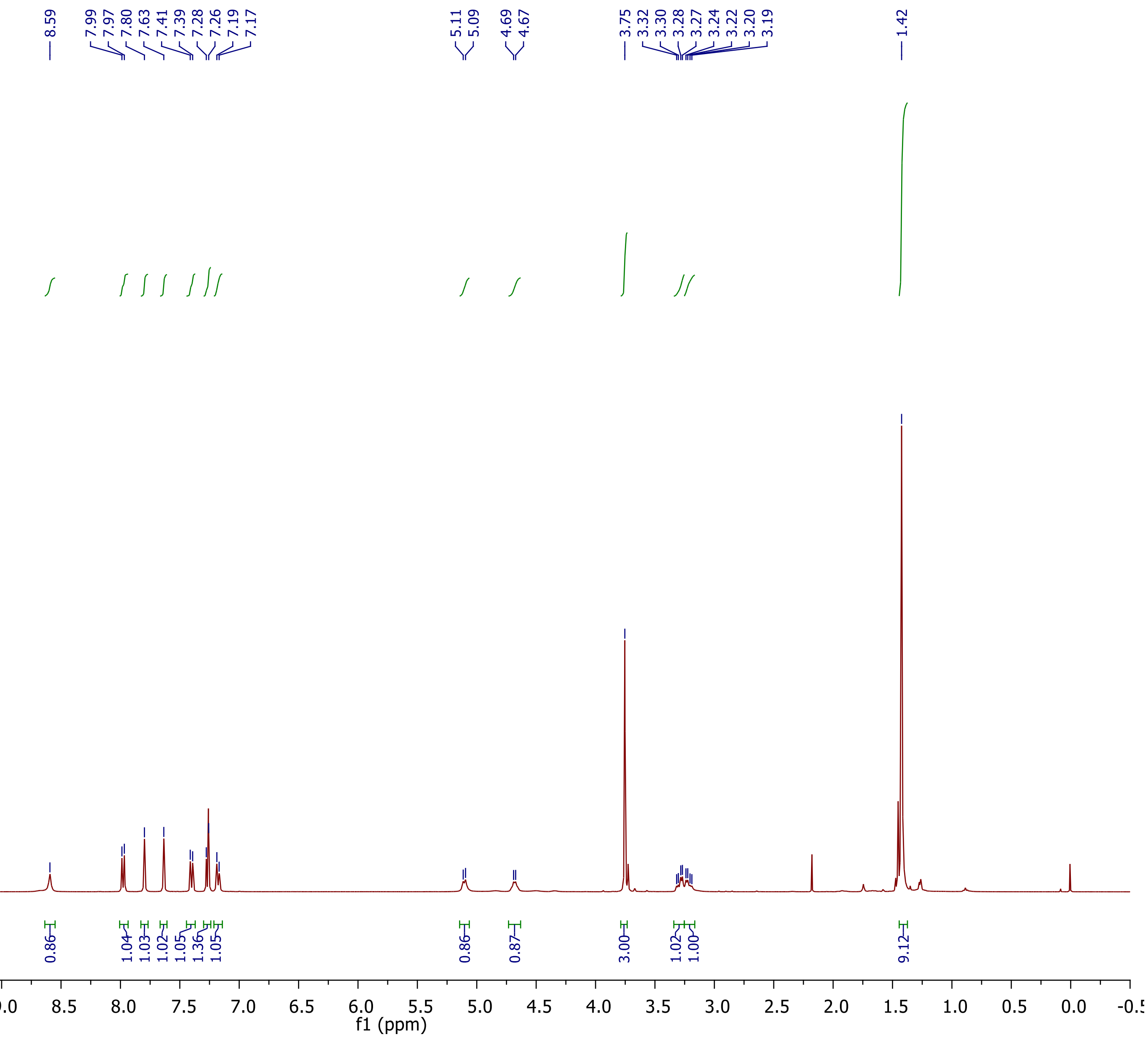

$^{13}C\{^1H\}$ NMR (101 MHz, $CDCl_3$)

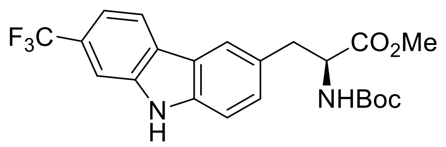

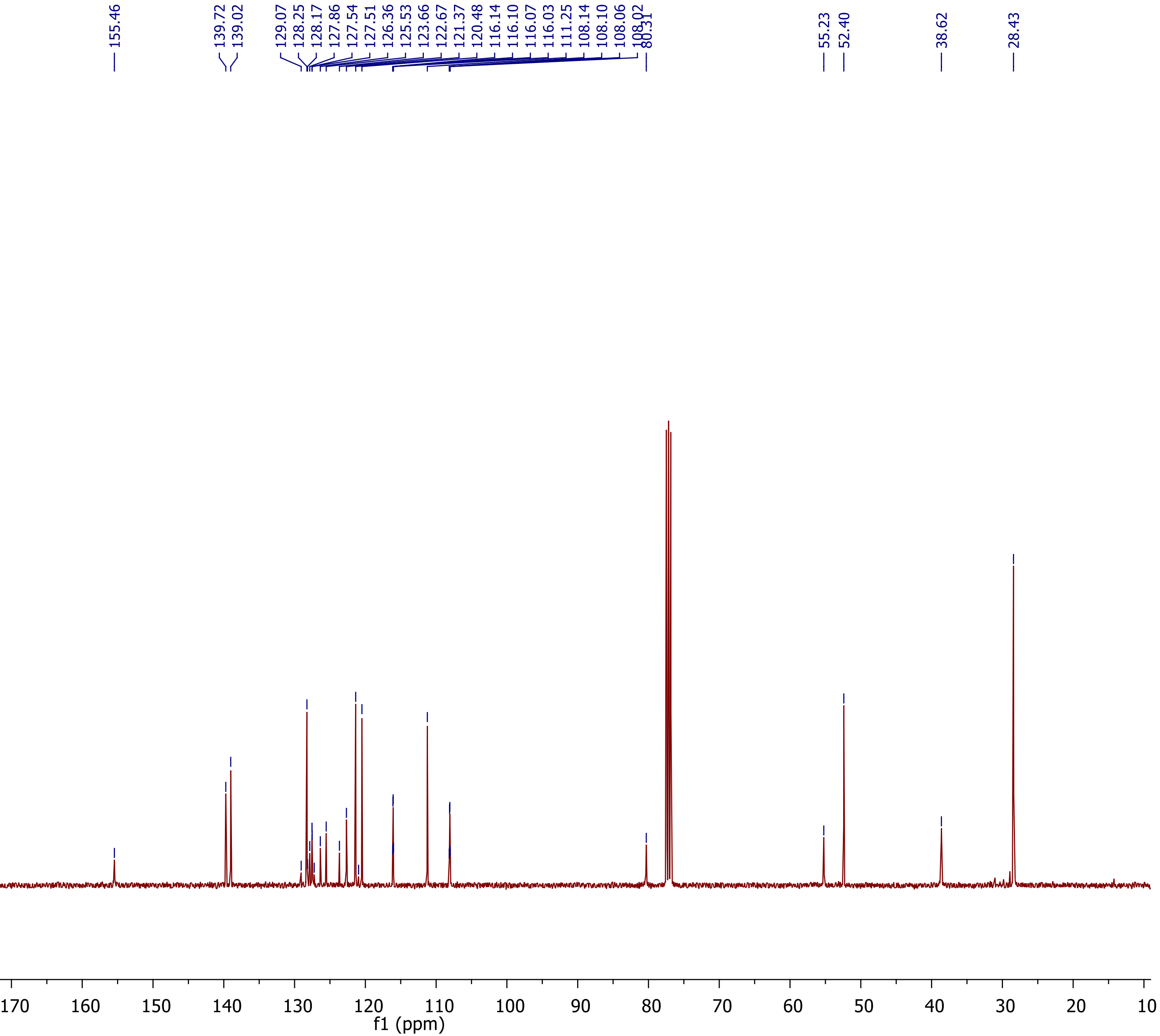

^{1}H NMR (400 MHz, CD_3OD)

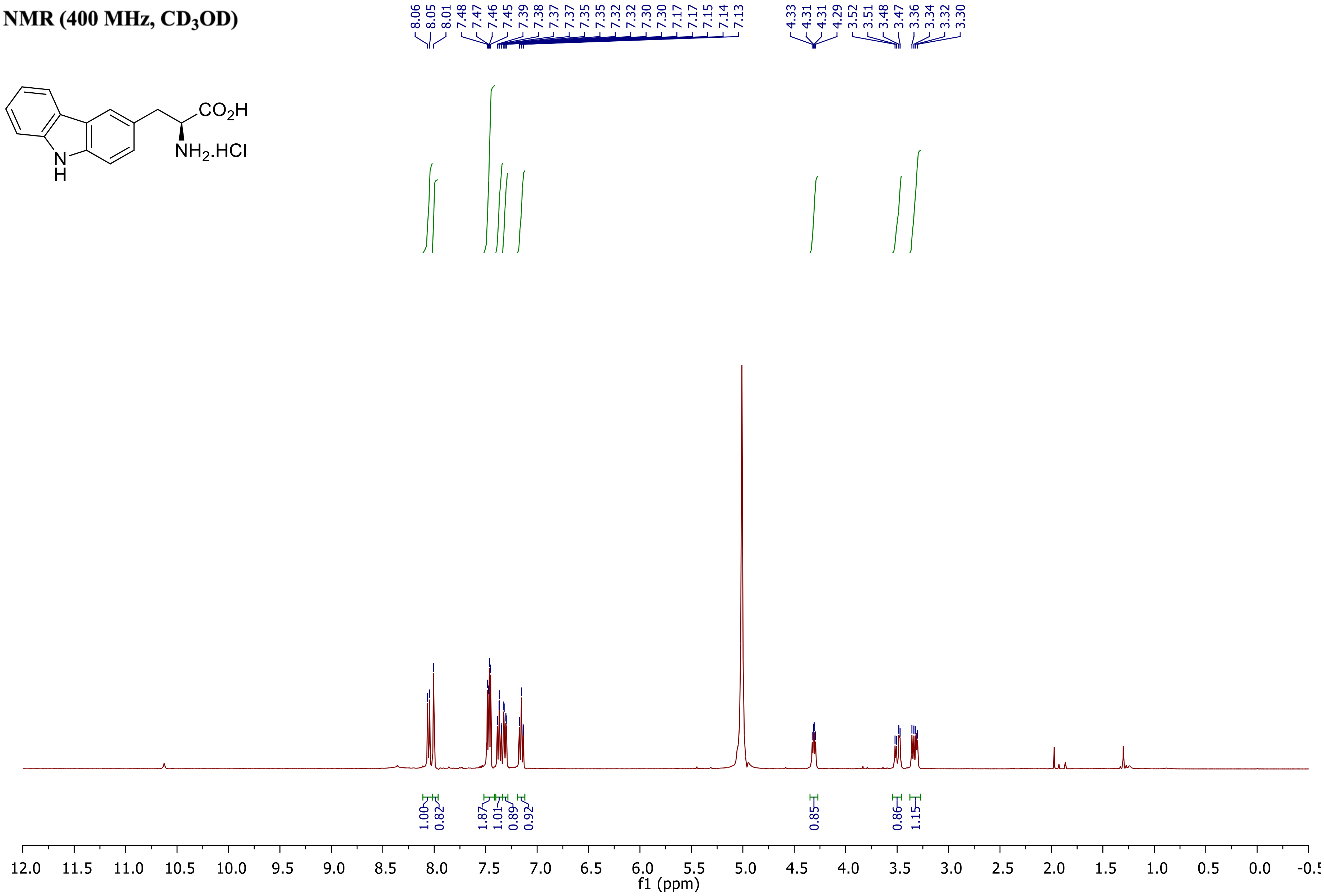

$^{13}C\{^{1}H\}$ NMR (101 MHz, CD_3OD)

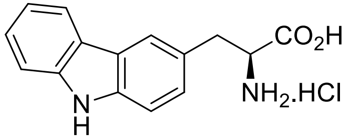

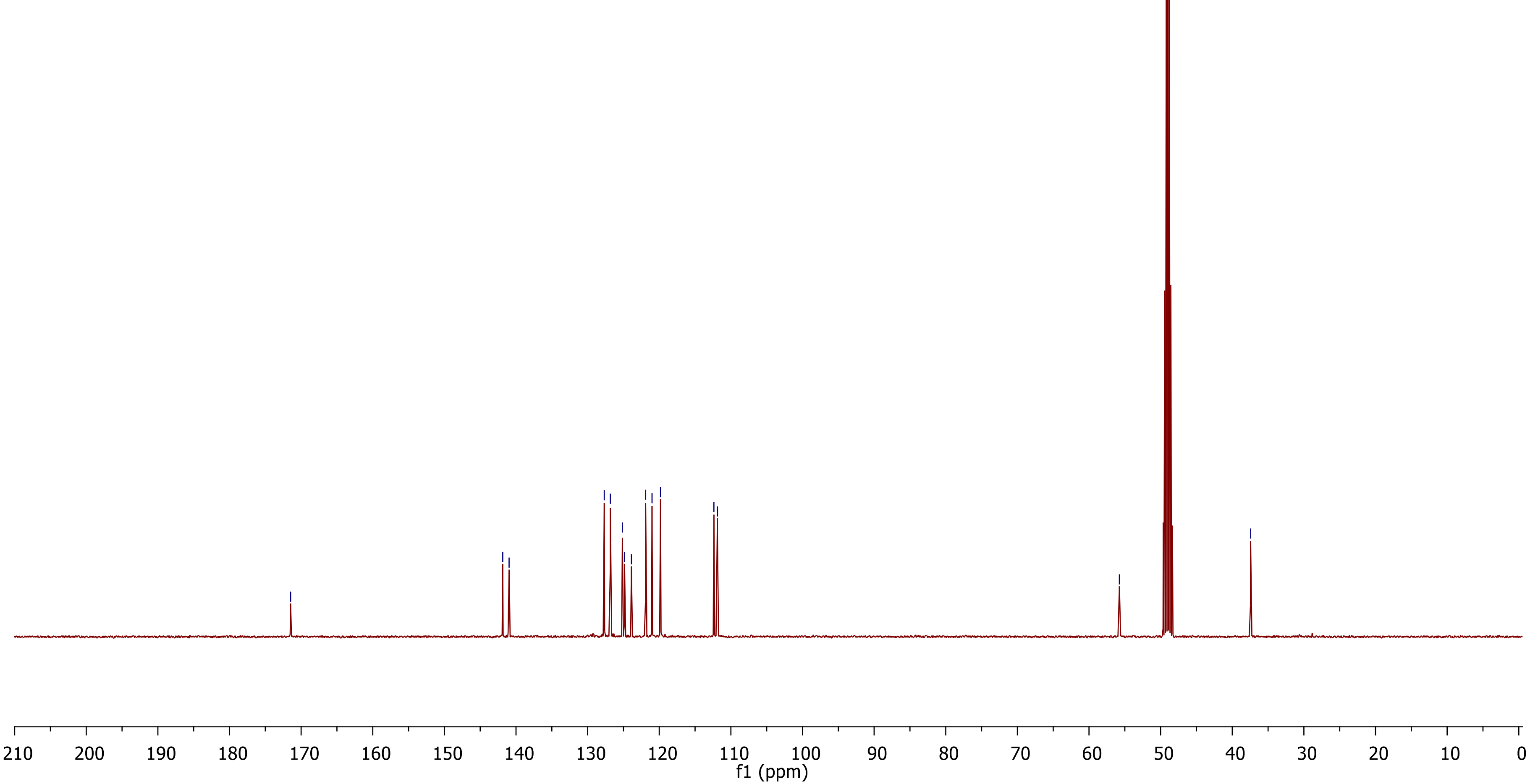

^{1}H NMR (400 MHz, CD_3OD)

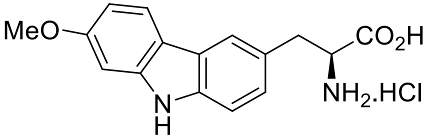

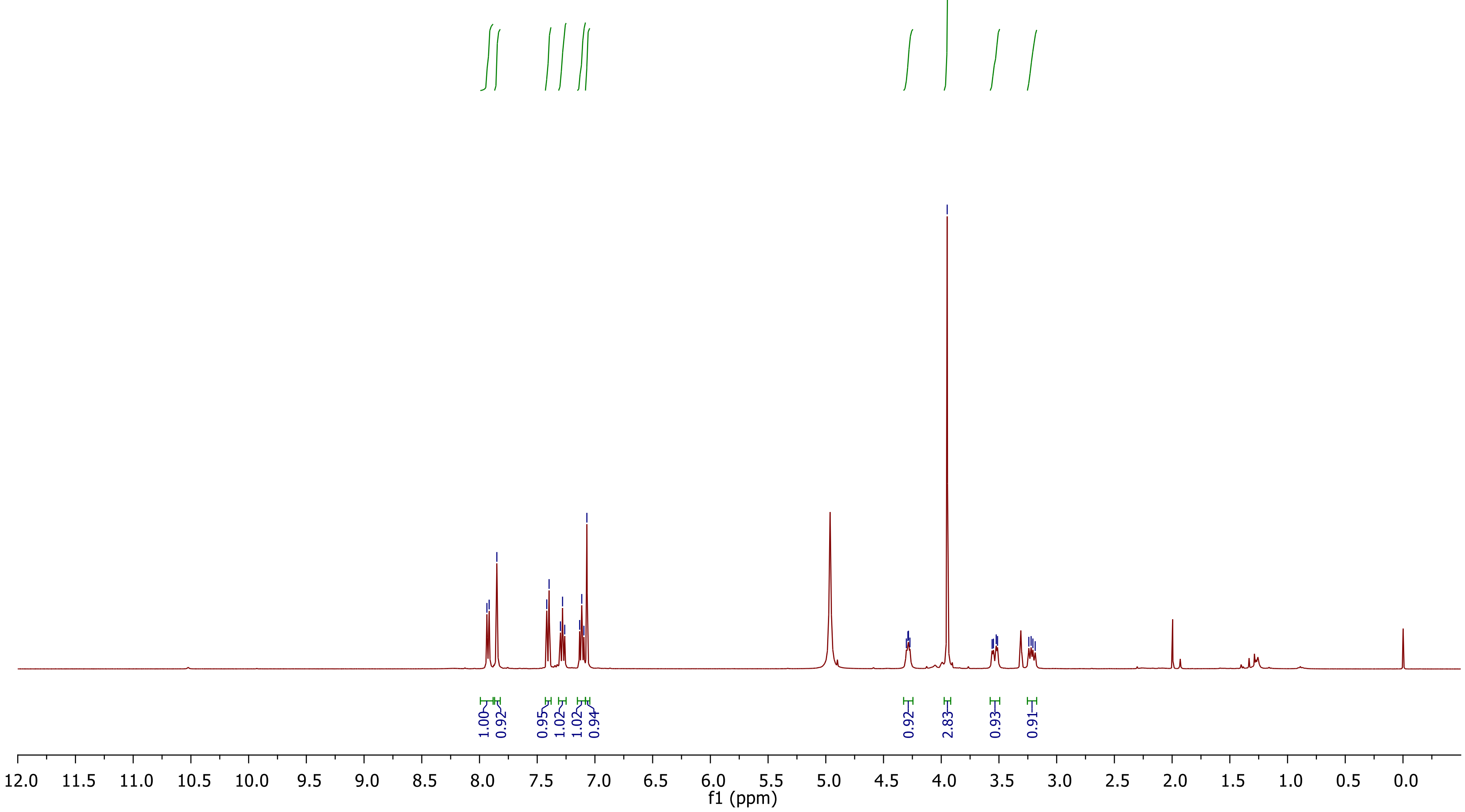

$^{13}C\{^1H\}$ NMR (101 MHz, CD_3OD)

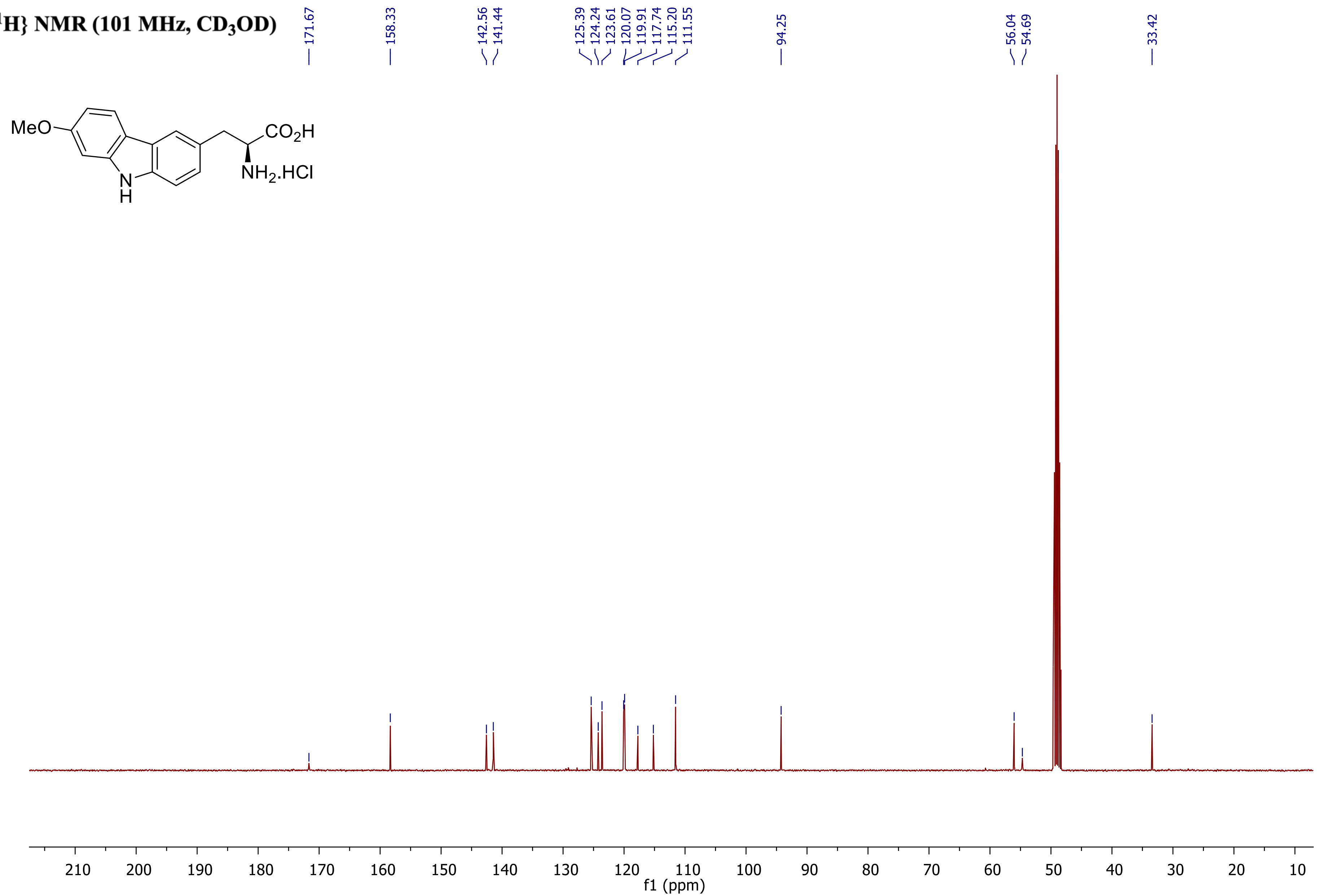

^1H NMR (400 MHz, CD_3OD)

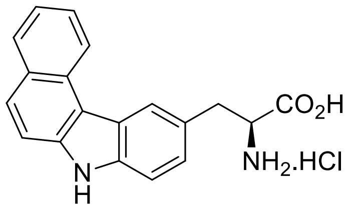

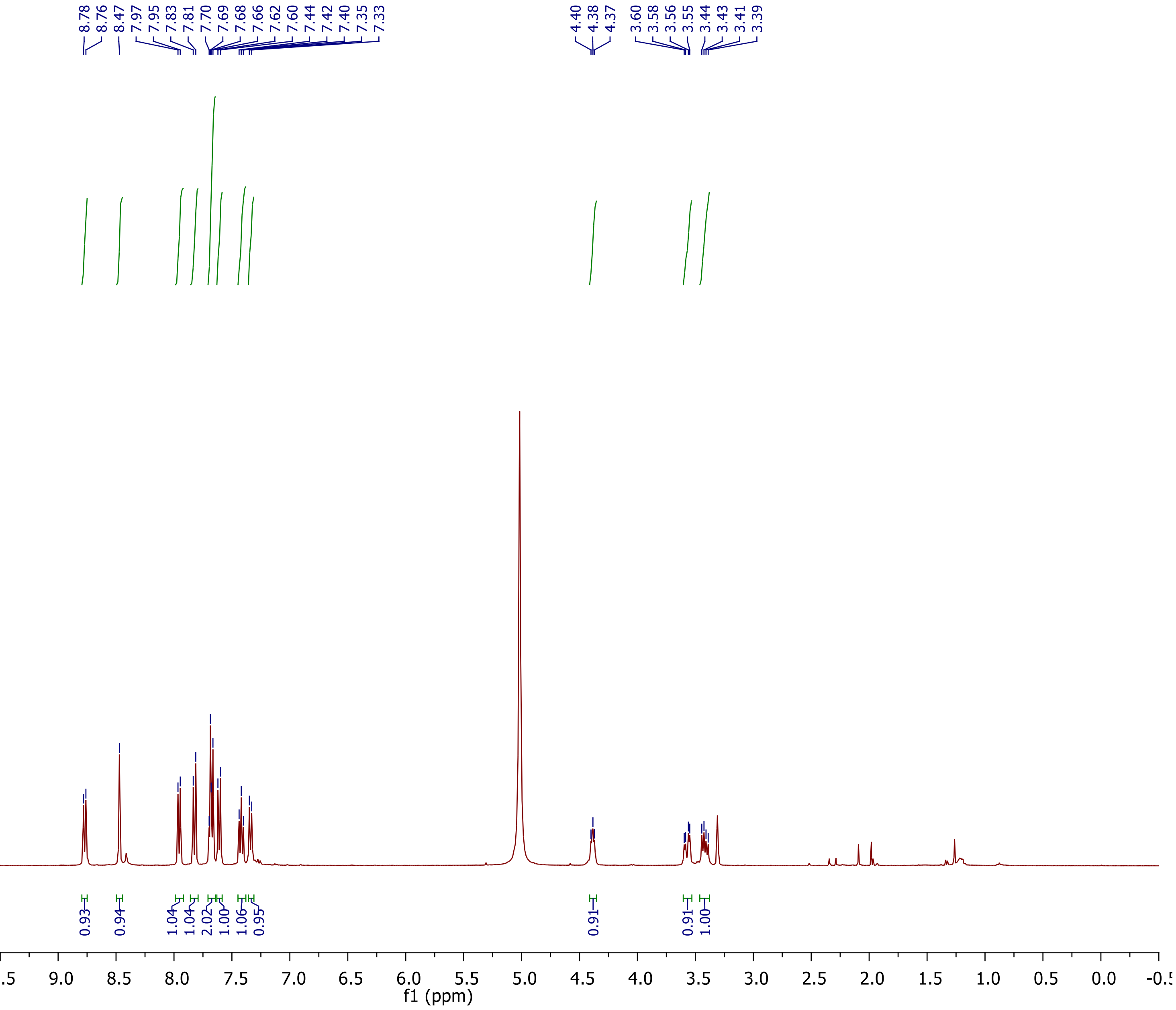

$^{13}C\{^{1}H\}$ NMR (101 MHz, CD_3OD)

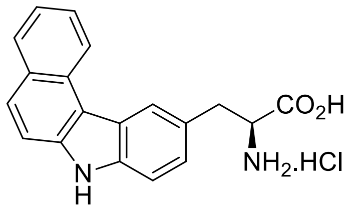

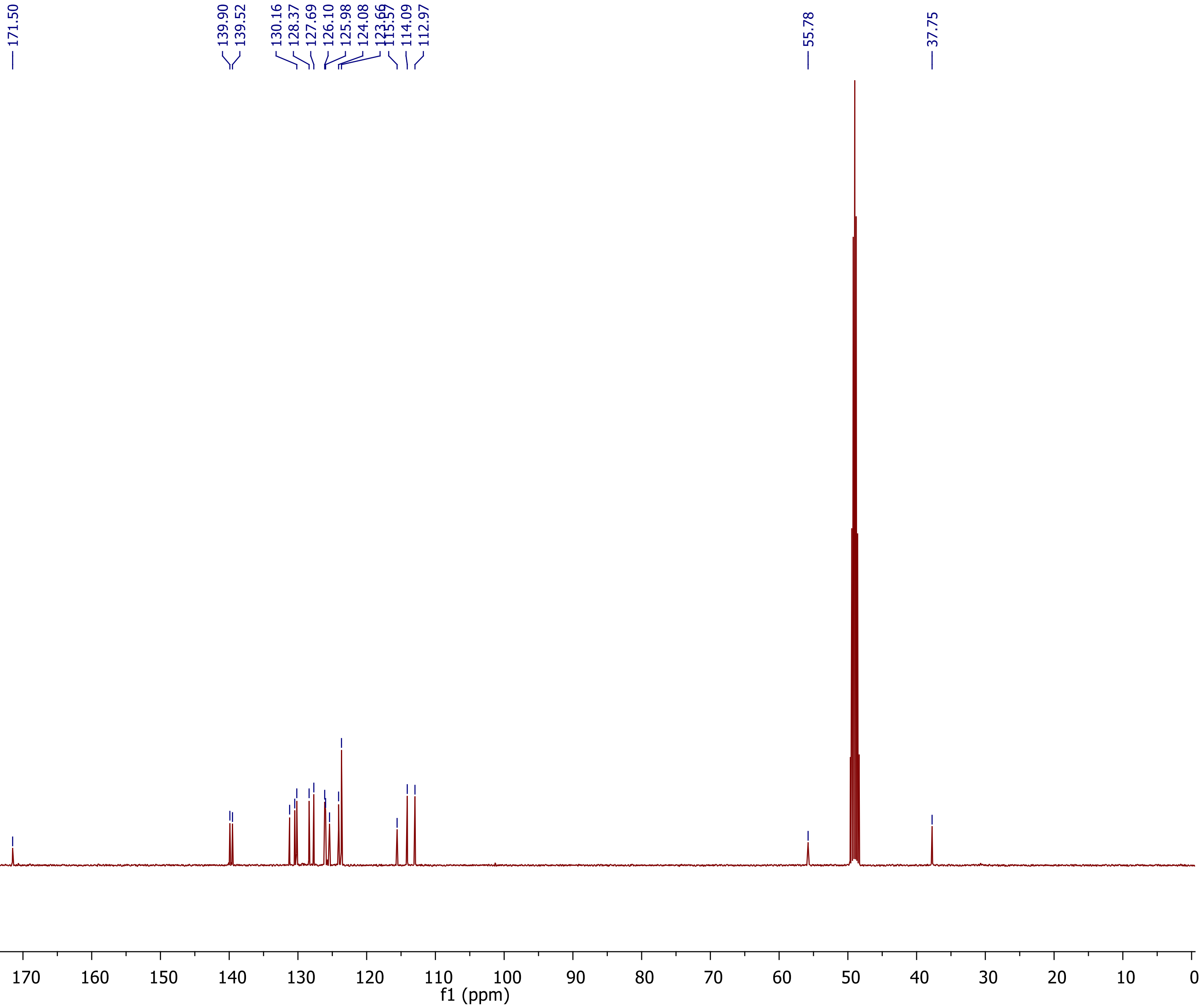

^{1}H NMR (400 MHz, DMSO-d_6)

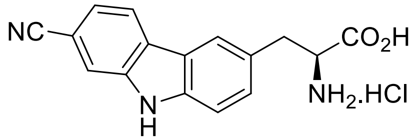

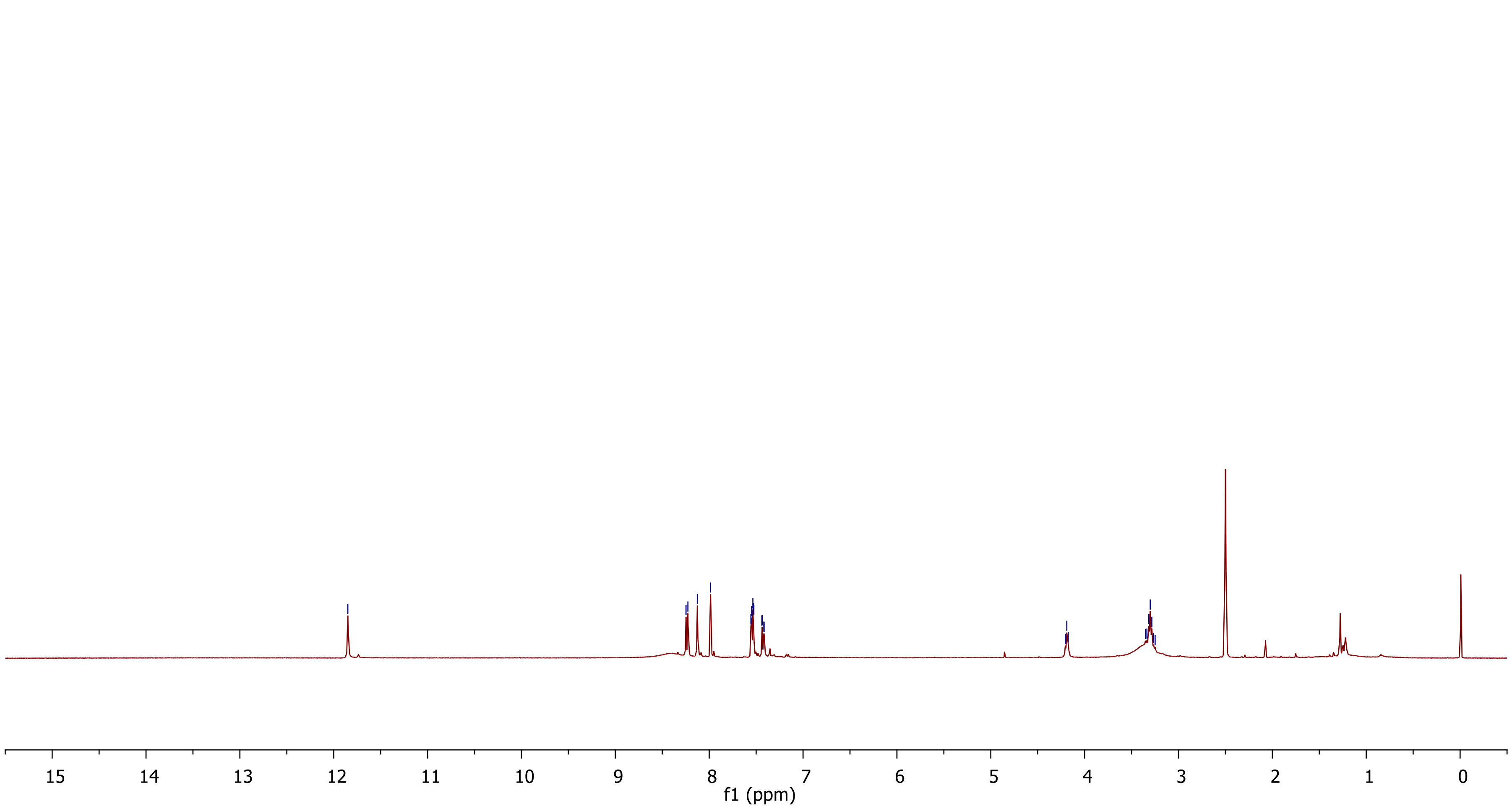

$^{13}C\{^{1}H\}$ NMR (101 MHz, DMSO-d_6)

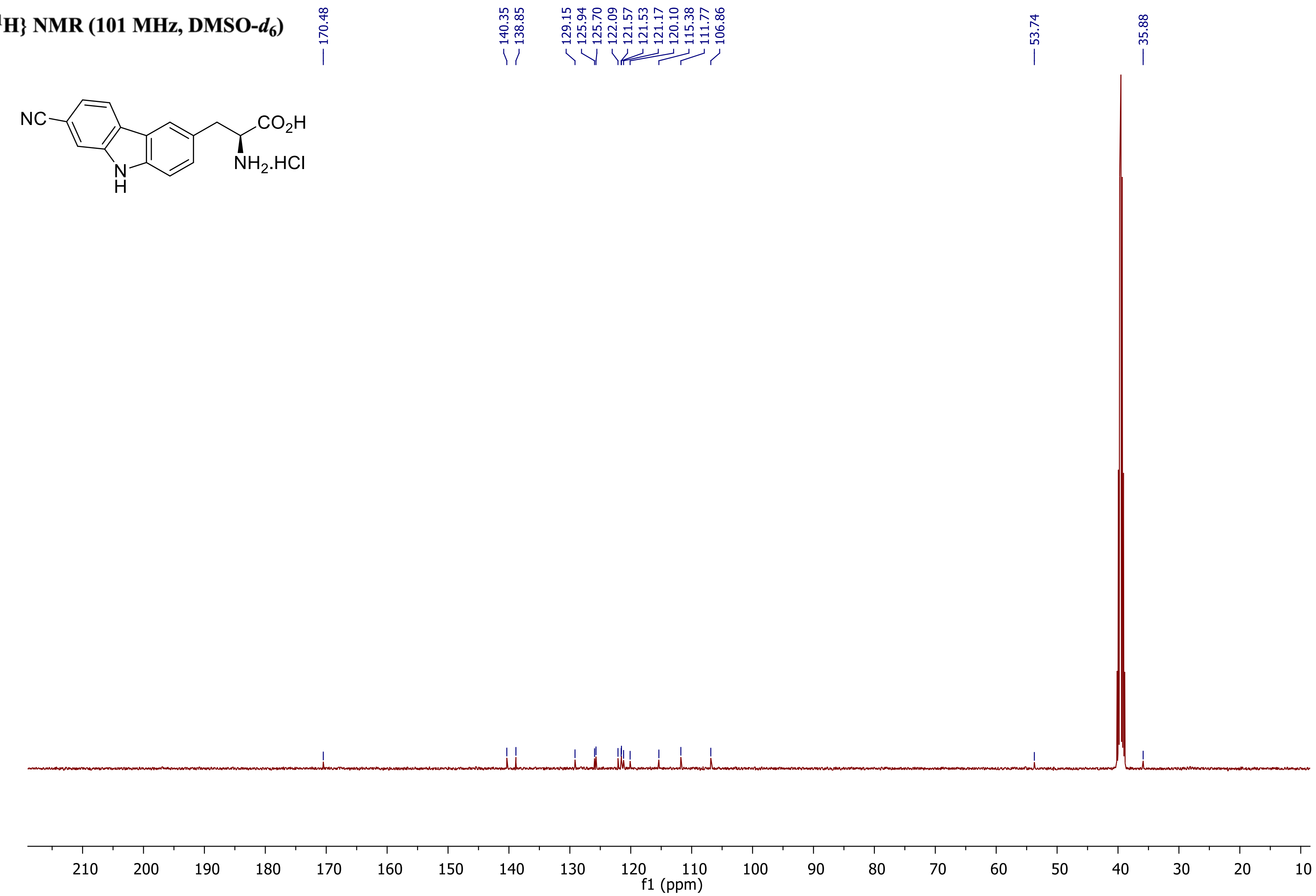

^{1}H NMR (400 MHz, CD_3OD)

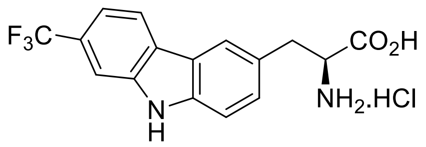

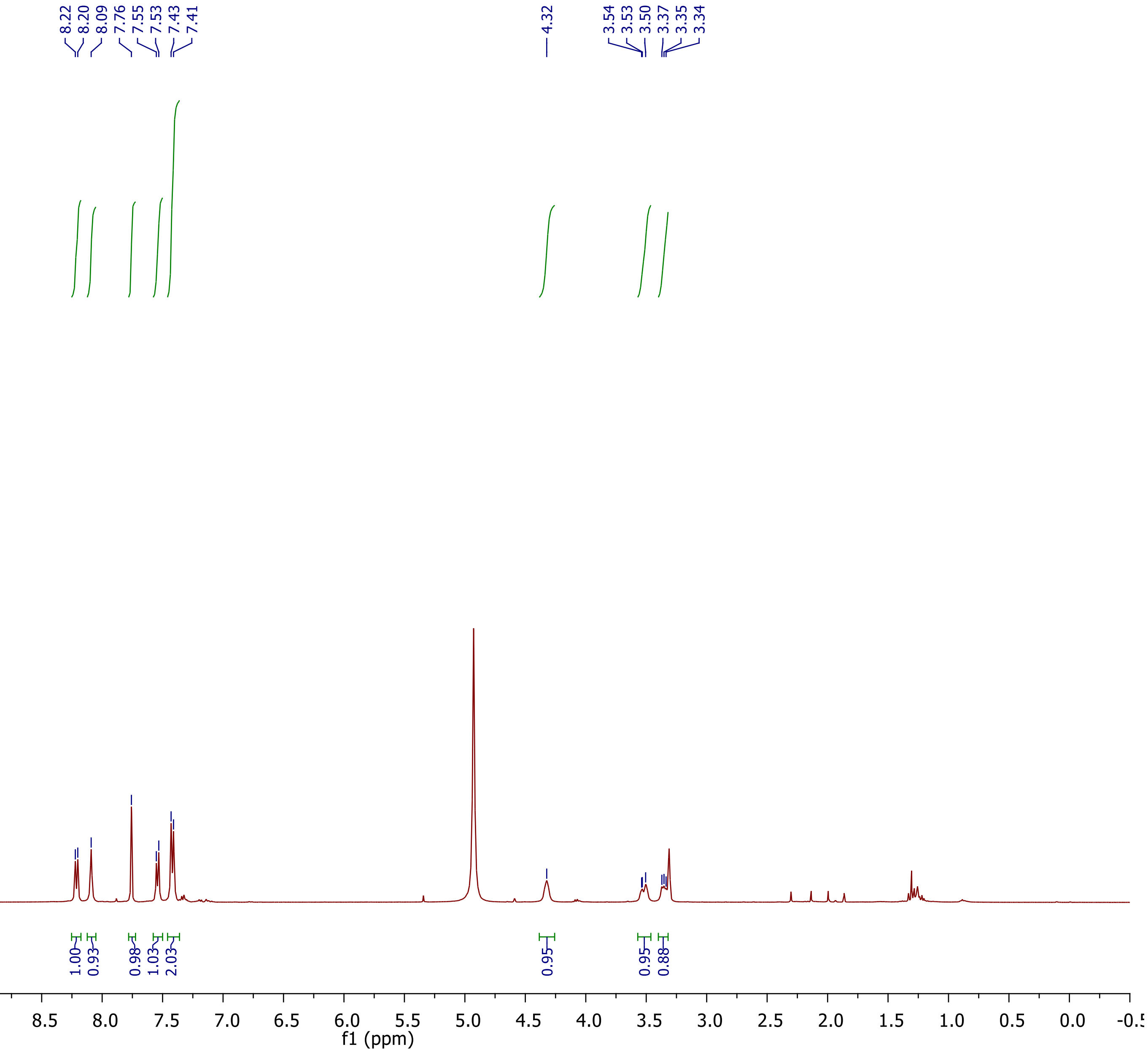

$^{13}C\{^1H\}$ NMR (101 MHz, CD_3OD)

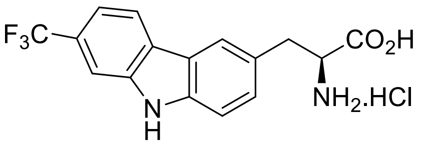

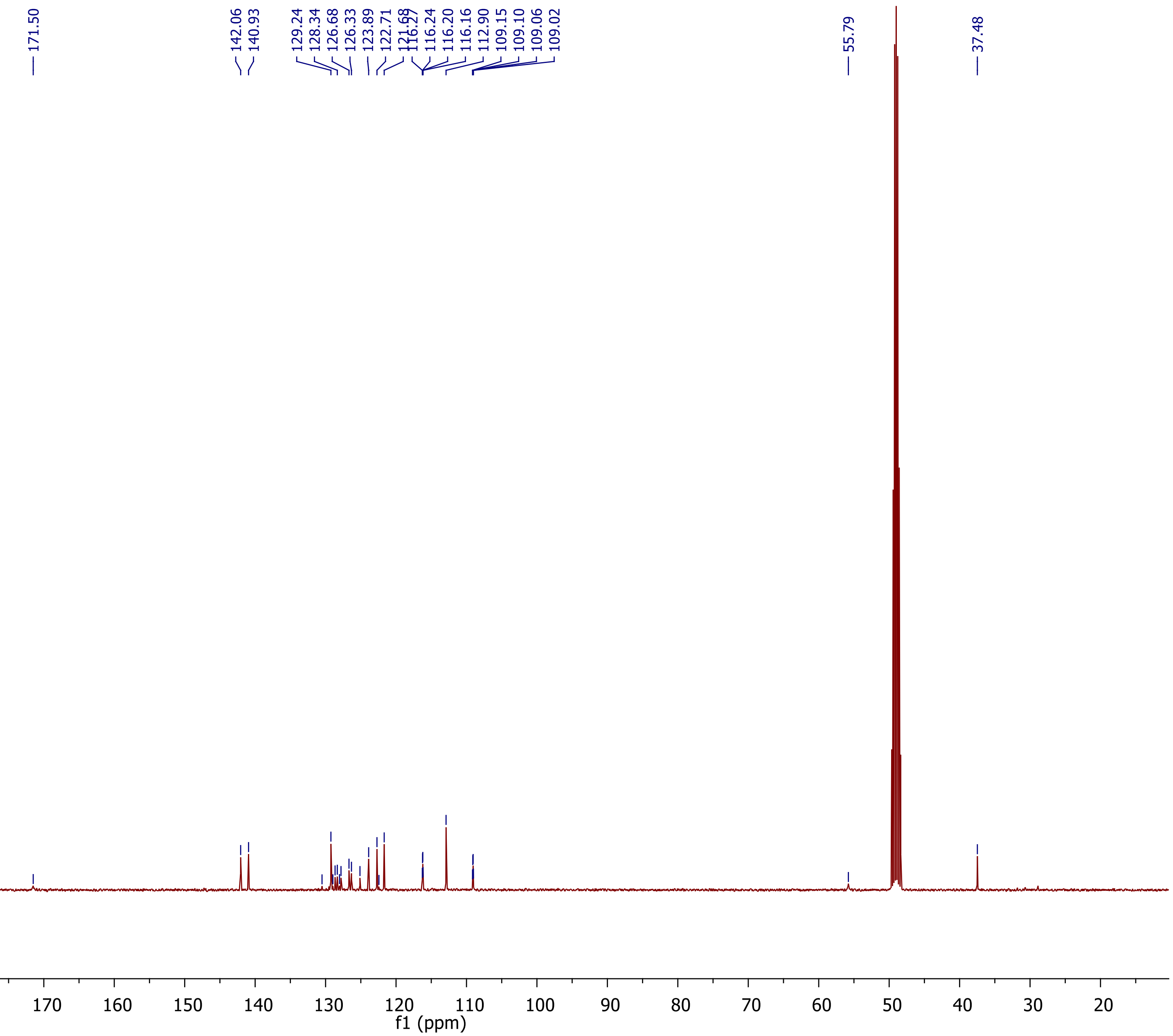

^{1}H NMR (400 MHz, CD_3OD)

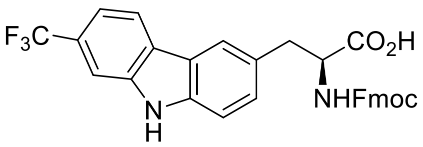

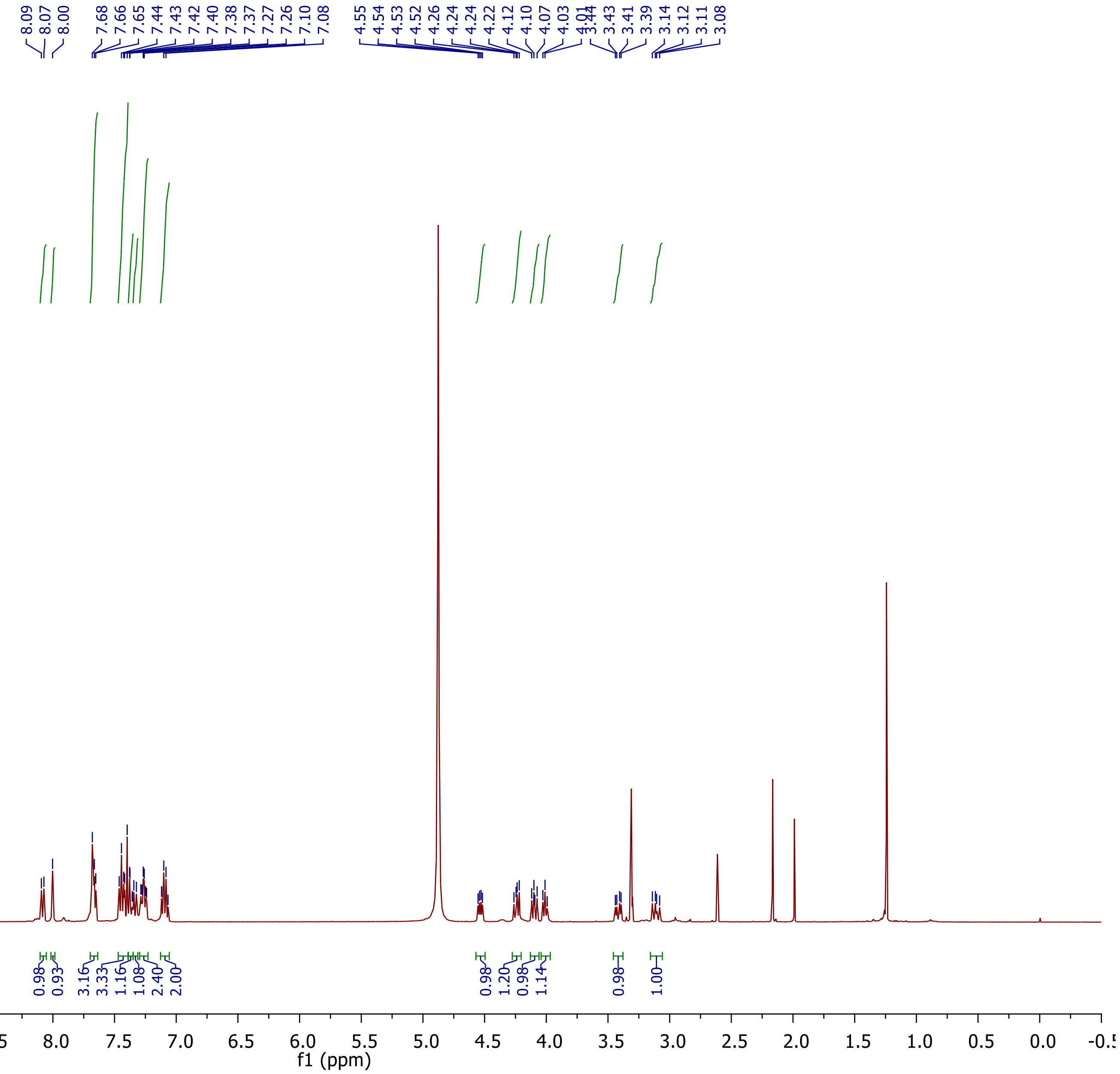

$^{13}C\{^{1}H\}$ NMR (101 MHz, CD_3OD)

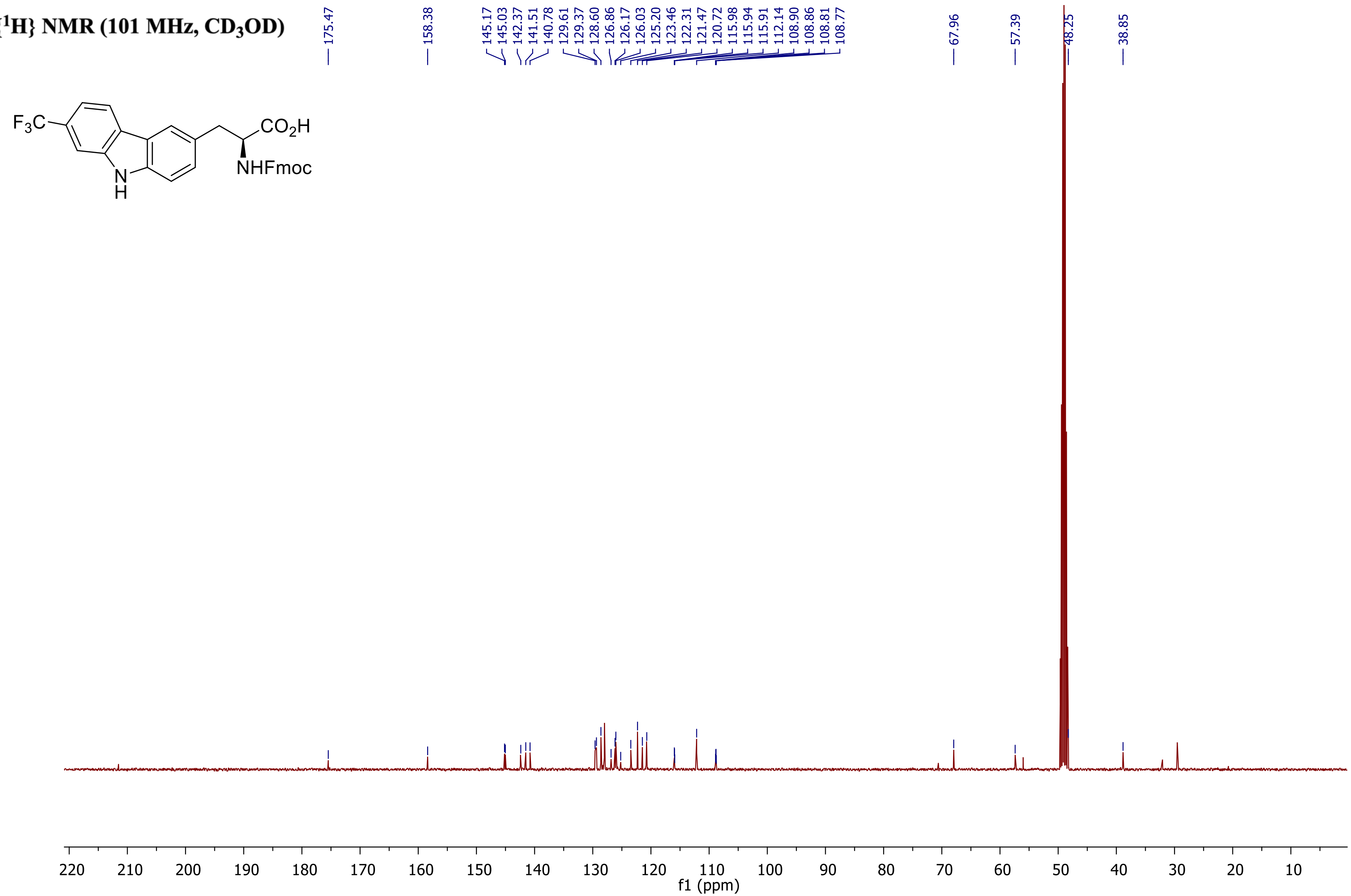

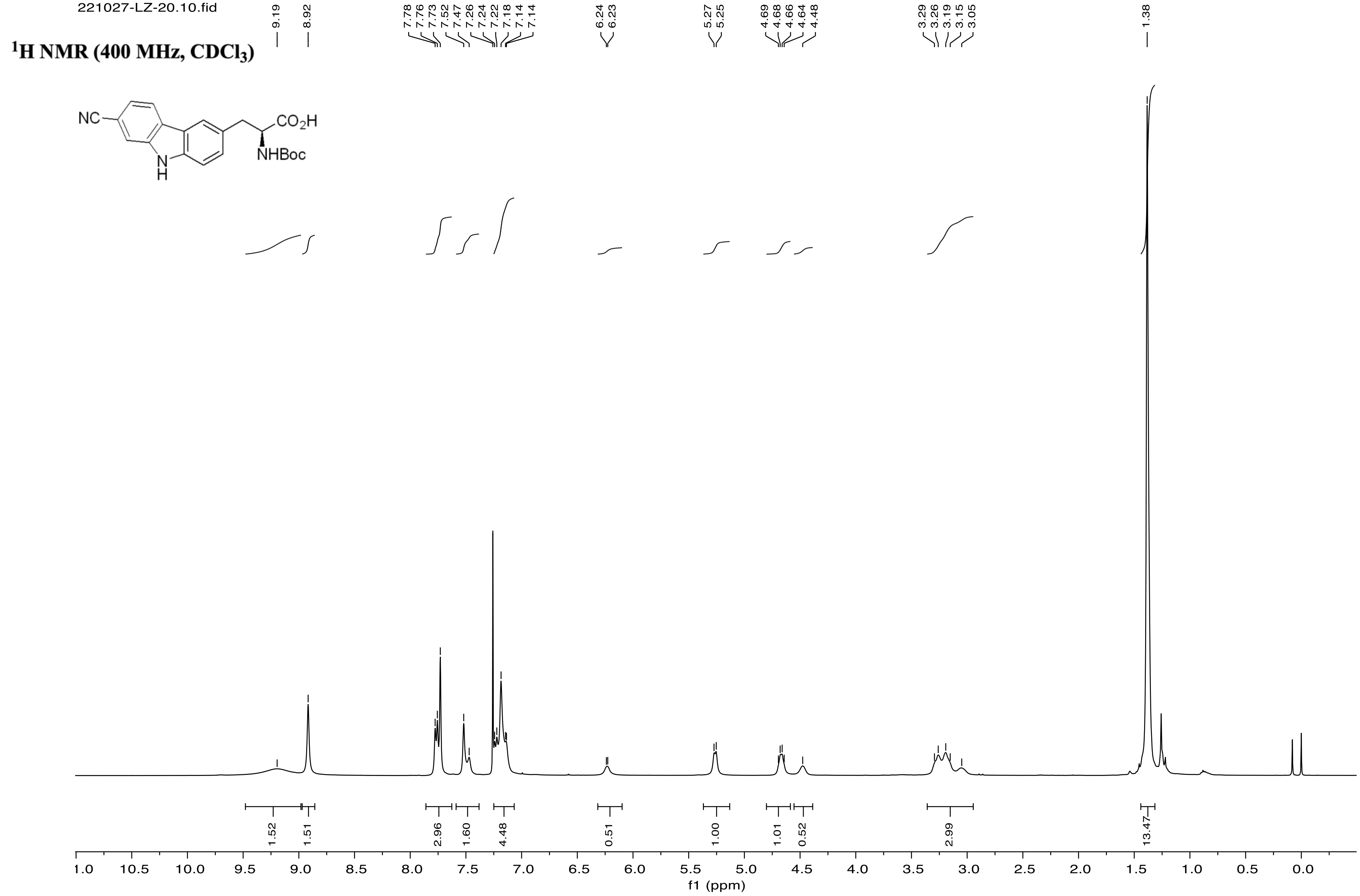
221027-LZ-20.10.fid
¹H NMR (400 MHz, CDCl₃)
NC
CO₂H
NHBoc
N
H
9.19
8.92
7.78
7.76
7.73
7.52
7.47
7.26
7.24
7.22
7.18
7.14
7.14
6.24
6.23
5.27
5.25
4.69
4.68
4.66
4.64
4.48
3.29
3.26
3.19
3.15
3.05
1.38
1.52
1.51
2.96
1.60
4.48
0.51
1.00
1.01
0.52
2.99
13.47
f1 (ppm)

$^{13}C\{^{1}H\}$ NMR (101 MHz, $CDCl_3$)

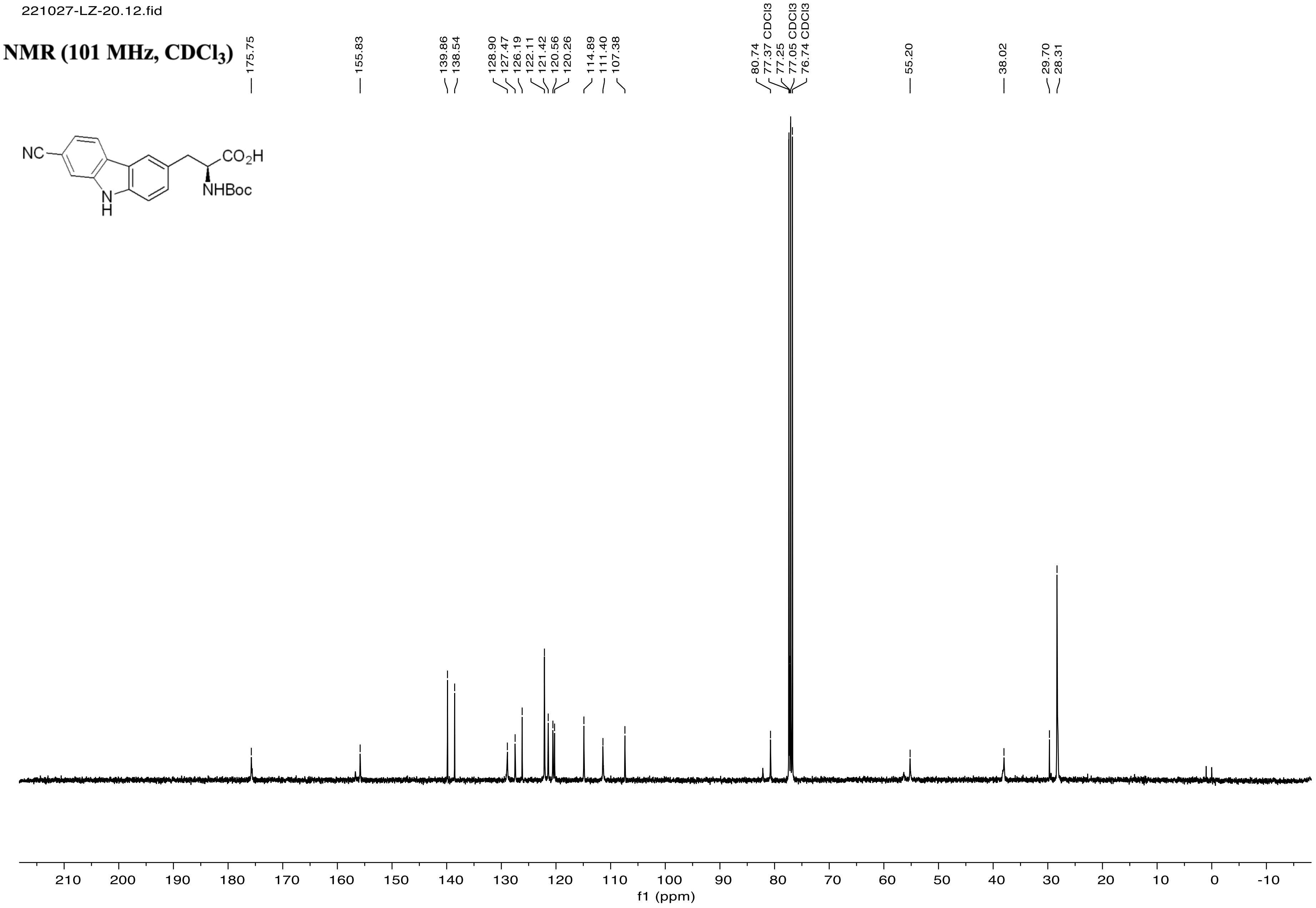

Supplement: SC-015-D4SC01173B-s001 [file SC-015-D4SC01173B-s001.pdf]
